# Nano-to-Submicron Hydroxyapatite Coatings for Magnesium-based Bioresorbable Implants – Deposition, Characterization, Degradation, Mechanical Properties, and Cytocompatibility

**DOI:** 10.1038/s41598-018-37123-3

**Published:** 2019-01-28

**Authors:** Qiaomu Tian, Jiajia Lin, Laura Rivera-Castaneda, Amit Tsanhani, Zachary S. Dunn, Alexis Rodriguez, Arash Aslani, Huinan Liu

**Affiliations:** 1 Department of Bioengineering, University of California, Riverside, CA 92521 USA; 2 Material Science & Engineering Program, University of California, Riverside, CA 92521 USA; 3 Microbiology Program, University of California, Riverside, CA 92521 USA; 4 Neuroscience Program, University of California, Riverside, CA 92521 USA; 5 N2 Biomedical LLC, One Patriots Park, Bedford, MA 01730 USA

## Abstract

Magnesium (Mg) and its alloys have shown attractive biocompatibility and mechanical strength for medical applications, but low corrosion resistance of Mg in physiological environment limits its broad clinical translation. Hydroxyapatite (HA) nanoparticles (nHA) are promising coating materials for decreasing degradation rates and prolonging mechanical strength of Mg-based implants while enhancing bone healing due to their osteoconductivity and osteoinductivity. Conformal HA coatings with nano-to-submicron structures, namely nHA and mHA coatings, were deposited successfully on Mg plates and rods using a transonic particle acceleration (TPA) process under two different conditions, characterized, and investigated for their effects on Mg degradation *in vitro*. The nHA and mHA coatings enhanced corrosion resistance of Mg and retained 86–90% of ultimate compressive strength after *in vitro* immersion in rSBF for 6 weeks, much greater than non-coated Mg that only retained 66% of strength. Mg-based rods with or without coatings showed slower degradation than the respective Mg-based plates in rSBF after 6 weeks, likely because of the greater surface-to-volume ratio of Mg plates than Mg rods. This indicates that Mg-based plate and screw devices may undergo different degradation even when they have the same coatings and are implanted at the same or similar anatomical locations. Therefore, in addition to locations of implantation, the geometry, dimension, surface area, volume, and mass of Mg-based implants and devices should be carefully considered in their design and processing to ensure that they not only provide adequate structural and mechanical stability for bone fixation, but also support the functions of bone cells, as clinically required for craniomaxillofacial (CMF) and orthopedic implants. When the nHA and mHA coated Mg and non-coated Mg plates were cultured with bone marrow derived mesenchymal stem cells (BMSCs) using the *in vitro* direct culture method, greater cell adhesion densities were observed under indirect contact conditions than that under direct contact conditions for the nHA and mHA coated Mg. In comparison with non-coated Mg, the nHA and mHA coated Mg reduced BMSC adhesion densities directly on the surface, but increased the average BMSC adhesion densities under indirect contact. Further long-term studies *in vitro* and *in vivo* are necessary to elucidate the effects of nHA and mHA coatings on cell functions and tissue healing.

## Introduction

Magnesium (Mg) and its alloys are a promising class of biodegradable metals for medical applications in musculoskeletal implants and urological devices^1–4^. Mg and its alloys have an elastic modulus of 41–45 GPa that is closer to that of human cortical bone than currently used titanium alloys, cobalt chromium alloys and stainless steels that are considered non-degradable in the body^5^. Therefore, for musculoskeletal applications, Mg-based implants reduce the effects of stress shielding on the fractured bone during healing process^6,7^. The main degradation product of Mg, i.e. magnesium ion (Mg^2+^), is one of the most abundant ions in human body, and can be naturally metabolized and resorbed^8,9^. Unfortunately, rapid degradation of Mg and its alloys in physiological environments is a major limiting factor for clinical translation of Mg-based implants, especially in the anatomical sites with abundant flow of body fluids and/or high mechanical load^10–12^. It has been reported that both abundant body fluids (e.g. blood) and mechanical stress could further accelerate degradation of Mg-based implants^11,12^. Moreover, rapid degradation of Mg-based implants may lead to local pH increase, hydrogen gas accumulation, and early mechanical failure that are undesirable in clinical applications.

Protective and bioactive coatings applied onto Mg substrates can potentially reduce the degradation rate^1^ and enhance healing, thus leading to a more functionally desirable implant. Hydroxyapatite (HA) is a biodegradable biocompatible ceramic and an inorganic component of human bone with desirable bioactivity for bone healing^13,14^. Furthermore, HA has demonstrated osteoconductive and osteoinductive capabilities, favorable for orthopedic implant applications^15–17^. Even though HA itself is limited for load-bearing musculoskeletal applications due to its inherent brittleness^18^, using HA as a bioactive coating material on load-bearing Mg provides complementary properties to each other. That is, Mg and its alloys serve as substrates to provide excellent mechanical strength for load-bearing, while HA serves as a coating to reduce the substrate degradation rate and simultaneously promote osteointegration at the implant interface^14,19–21^. In comparison with conventional micro-sized HA (mHA), nano-sized HA (nHA) could provide several advantages such as better bioresorbability and less brittleness^22–24^, and enhance osteoblast adhesion and proliferation^10,25,26^. However, it is challenging to produce conformal and consistent coatings of nHA on Mg-based substrates. The effects of the HA particle size coupled with transonic particles acceleration (TPA) deposition process on coating characteristics, degradation properties, mechanical properties, and cytocompatibility have never been studied yet on Mg-based fixation devices such as plates and screws.

The objective of this study was to deposit and characterize conformal HA coatings on Mg plates (7.5 mm × 1 mm) and rods (7.5 mm × 15 mm) that mimic the typical dimensions of a plate and a screw used in bone repair, as well as evaluating the *in vitro* degradation, mechanical properties, and cytocompatibility of nHA and mHA coated Mg for craniomaxillofacial (CMF) and orthopedic implant applications, especially for fixation devices used in load-bearing conditions. HA particles with different size distribution, namely nano-sized HA (nHA) and micro-sized HA (mHA), were deposited on Mg plate and rod substrates under two different conditions of a patented TPA process and characterized thoroughly. The nHA and mHA coated Mg plates and rods were immersed in revised simulated body fluid (rSBF) to elucidate their degradation behaviors; specifically, their microstructures, compositions, and phases were examined before and after immersion. To identify the most promising candidate for future *in vivo* studies, the *in vitro* degradation rates, mechanical properties, and cytocompatibility with bone marrow derived mesenchymal stem cells (BMSCs) were studied and compared.

## Materials and Methods

### Prepare nHA and mHA conformal coatings on Mg plates and rods using the patented transonic particle acceleration process

Pure Mg rods with a purity of 99.9% and a diameter of 7.9 mm (as drawn, Goodfellow Corporation, Coraopolis, PA) were machined into Mg plates (7.5 mm × 1 mm) and Mg rods (7.5 mm × 15 mm) respectively to mimic the typical dimensions of a plate and screw used in CMF reconstruction. Pure Mg plates with a diameter of 7.5 mm and a height of 1 mm, and pure Mg rods with a diameter of 7.5 mm and a height of 15 mm were cleaned in ethanol for 10 minutes using an ultrasonic bath before serving as substrates for HA coating deposition. Powders of nano-sized hydroxyapatite (nHA) or micron-sized hydroxyapatite (mHA) [Ca_10_(PO_4_)_6_(OH)_2_] (Himed, Old Bethpage, NY) were respectively deposited on all the surfaces of the Mg plates and rods using the transonic particle acceleration (TPA) deposition process, also known as IonTite^TM^. TPA is a proprietary inhouse coating process at N2 Biomedical (formerly Spire Biomedical Inc.)^27^ for depositing thin films of ceramics and other materials onto various surfaces at low temperature and under precisely controlled conditions. The TPA is capable of producing conformal coatings on three-dimensional (3D) substrates. The high-pressure gas (50 to 400 psi) was introduced into a nozzle and accelerated in the throat region of the nozzle. In the meantime, a process gas was introduced through a gas control module to a powder-metering device that contained nHA or mHA particles. The TPA process accelerated the powders to sub-sonic speeds, and deposited the particles onto Mg substrates at room temperature using the high-pressure gas. In this study, the distance between the nozzle and the Mg substrates was set at 5–8 cm and kept the same for each type of samples; the process gas was pure nitrogen (N_2_) for all samples; and the process temperature was room temperature for all samples. In order to identify an optimal deposition process for nHA and mHA coatings on Mg, two different pressure conditions were applied during the TPA deposition process. Specifically, nHA and mHA deposited on Mg substrates under a low deposition pressure of 100 psi were referred to as nHA_100 and mHA_100, respectively; their counterparts of nHA_400 and mHA_400 were deposited on Mg substrates using a high deposition pressure of 140 psi. Pure Mg substrates without HA coating were included in this study as a control.

### Characterize nHA and mHA coatings on Mg substrates after the TPA process and the corresponding nHA and mHA powders used for the TPA process

The nHA_100, nHA_400, mHA_100, mHA_400 coated Mg rods, and non-coated Mg rod control, nHA powder, and mHA powder were mounted on a flat SEM holder (Ted Pella) using double-sided copper tape and sputter coated with platinum/palladium at 20 mA for 60 seconds. The microstructures of nHA_100, nHA_400, mHA_100, mHA_400, and Mg control, as well as the nHA and mHA powders used for coating deposition, were examined under a scanning electron microscope (SEM; Nova NanoSEM 450, FEI Inc.) at a high vacuum mode. The size distributions of HA in the coatings after deposition were determined based on SEM images using the ImageJ analysis tools. Specifically, individual particles were manually outlined using ImageJ to measure the size and calculate the distribution on the different coatings. The surface elemental compositions of the samples were determined using energy dispersive x-ray spectroscopy (EDS; Aztec, Oxford instrument) at an acceleration voltage of 20 kV. The top circular surfaces of the cylindrical rod samples were polished to remove the coating and expose the edges; and each sample was then mounted onto a flat SEM holder using the double-sided copper tape to visualize the cross-sections of the coatings and the coating/Mg interface at the edge of the circular surface. The thickness of the coatings was determined based on the SEM images of cross-sections using ImageJ analysis tools. The phase and crystal structure of the coatings and the source powders were analyzed using X-ray diffraction (XRD; Empyrean, PANalytical) at 45 KV and 40 mA with 2θ angles from 10° to 80° at a step size of 0.002°. The diffraction peaks were identified based on the international center for diffraction data (ICDD) database using HighScore software (PANAlytical).

### Immersion degradation of nHA and mHA coated Mg plates and rods in rSBF *in vitro*

The degradation behaviors of Mg plates (7.5 mm × 1 mm) and Mg rods (7.5 mm × 15 mm), i.e., nHA_100, nHA_400, mHA_100, mHA_400, and Mg control, were investigated *in vitro* via immersion in rSBF. Before immersion, all samples were weighed and disinfected under ultraviolet (UV) radiation for 4 hours. The Mg plates were placed vertically with the circular base on the bottom of culture well and Mg rods were placed horizontally with the cylindrical side on the bottom of culture well in 12-well tissue culture plates and 3 mL of rSBF was added to each well. The height of Mg rod was 15 mm, greater than the depth of a 12-well plate, and, thus, the rod is placed horizontally in the well so that the environmental conditions can be kept the same for all the rod and plate samples. The volume of rSBF as immersion media was kept the same at 3 mL for all the plate and rod samples. The samples were incubated in rSBF under a standard cell culture condition (a sterile, 37 °C, 5% CO_2_/95% air, and humidified environment) until the prescribed time points. The prescribed time points were 12 hrs, 24 hrs, 48 hrs, 72 hrs, 1 week, 2 weeks, 4 weeks and 6 weeks.

After each time point, rSBF was collected from the wells, and the samples were dried in an oven at 37 °C. To ensure each sample is completely dried, Mg plates were dried for 24 hours and Mg rods were dried for 72 hours due to their larger size. The dried samples were then weighed and photographed. The mass change of each samples before and after immersion was calculated. Specifically, the mass of the samples (M_i_) after each incubation time was divided by its initial mass (M_0_) to obtain the mass ratio (M_i_/M_0_). The pH of the media was measured using a pre-calibrated pH meter (SB70Pm, SympHony, VWR). The pH increase of rSBF cultured with Mg-based samples, i.e., pH_(+)_, was obtained by subtracting the baseline pH_(rSBF)_ (i.e., the pH of rSBF) from the pH of the media cultured with each sample, i.e., pH_(sample)_. That is, pH_(+)_ = pH_(sample)_ − pH_(rSBF)_. The Mg^2+^ and Ca^2+^ ion concentrations were quantified using inductive coupled plasma - optical emission spectrometry (ICP-OES; Optima 8000, PerkinElmer, Waltham, MA, USA). Briefly, the collected solutions from each well were diluted with DI water by a factor of 1:100 into a total volume of 10 mL, and then fed into ICP-OES together with the respective standards to measure Mg^2+^ and Ca^2+^ ion concentrations. Mg^2+^ ion concentrations were calcualted based on the calibration curve generated using Mg^2+^ standards (PerkinElmer) serially diluted to a concentration of 0.5, 1, 2, and 5 mg/L. Similarly, Ca^2+^ ion concentrations were calculated based on the calibration curve generated using Ca^2+^ standards (PerkinElmer) serially diluted to a concentration of 0.05, 0.5, and 5 mg/L. After these characterizations, the samples were then placed back into 3 mL of fresh rSBF solution and incubated until the next prescribed time point. Similar testing was repeated for each prescribed time cycle. Each sample type was tested in triplicate for the degradation studies.

After 6 weeks of immersion, all the samples, including Mg plates (7.5 mm × 1 mm) and Mg rods (7.5 mm × 15 mm), were dried and placed on a conductive copper tape and sputter coated for SEM imaging and EDS analyses, as described previously. The phases and crystal structures of the degradation products on the Mg plates (7.5 mm × 1 mm) and rods (7.5 mm × 15 mm) were analyzed using X-ray diffraction (XRD; Empyrean, PANalytical) at 45 KV and 40 mA with 2θ angles from 10° to 80° at a step size of 0.002°. Specifically, the diffraction peaks of degradation products were identified based on the international center for diffraction data (ICDD) database using HighScore software (PANAlytical). The degradation products on the Mg rods (7.5 mm × 15 mm) were scratched off and collected for analyzing chemical bonding structures using Fourier transform infrared spectroscopy (FTIR, Optical 8000, Bruker) under the reflective mode.

### Calculate the daily release rates of Mg^2+^ ions from the degradation of plate-shaped and rod-shaped samples *in vitro*

The average daily *in vitro* Mg^2+^ release rates of the nHA and mHA coated Mg, as well as non-coated Mg plates or rods, were calculated based on the Mg^2+^ ion concentrations at each time point, and normalized by initial surface area, initial volume, and initial mass, respectively, following the equations 1a, 1b, and 1c^12^:

1a $$\frac{{\rm{avg}}.\,{\rm{daily}}\,{{\rm{Mg}}}^{2+}\,{\rm{release}}\,{\rm{rate}}}{{\rm{unit}}\,{\rm{initial}}\,{\rm{surf}}.\,{\rm{area}}}=\frac{[{\sum }_{{\rm{i}}=1}^{42}({[{{\rm{Mg}}}^{2+}]}_{{\rm{i}}})-{[{{\rm{Mg}}}^{2+}]}_{{\rm{rSBF}},{\rm{tot}}}]\times 0.003{\rm{L}}}{42\,{\rm{day}}\times {{\rm{SA}}}_{0}}\,$$

1b $$\frac{{\rm{avg}}.\,{\rm{daily}}\,{{\rm{Mg}}}^{2+}\,{\rm{release}}\,{\rm{rate}}}{{\rm{unit}}\,{\rm{initial}}\,{\rm{vol}}.}=\frac{[{\sum }_{{\rm{i}}=1}^{42}({[{{\rm{Mg}}}^{2+}]}_{{\rm{i}}})-{[{{\rm{Mg}}}^{2+}]}_{{\rm{rSBF}},{\rm{tot}}}]\times 0.003{\rm{L}}}{42\,{\rm{day}}\times {{\rm{V}}}_{0}}$$

1c $$\frac{{\rm{avg}}.\,{\rm{daily}}\,{{\rm{Mg}}}^{2+}\,{\rm{release}}\,{\rm{rate}}}{{\rm{unit}}\,{\rm{initial}}\,{\rm{mass}}}=\frac{[{\sum }_{{\rm{i}}=1}^{42}({[{{\rm{Mg}}}^{2+}]}_{{\rm{i}}})-{[{{\rm{Mg}}}^{2+}]}_{{\rm{rSBF}},{\rm{tot}}}]\times 0.003{\rm{L}}}{42\,{\rm{day}}\times {{\rm{M}}}_{0}}$$

In Eq. 1a, Eq. 1b, and Eq. 1c, [Mg^2 +^]_i_ was referred to the Mg^2+^ ion concentration at each time point over 6 weeks, measured as mg/L using ICP-OES. [Mg^2 +^]_rSBF, tot_ was referred to the total Mg^2+^ concentration in blank rSBF over 6 weeks that was 310 mg/L for 10 time points. The volume of rSBF used in the immersion study was 3 mL (0.003 L) for every sample. The average daily Mg^2+^ release rate was calculated based on the 6-week immersion because the immersion degradation was carried out in 6 weeks (42 days). In this study, the average daily Mg^2+^ release rate was normalized by either the initial surface area (SA_0_), the initial volume (V_0_), or the initial mass (M_0_) of the plates or rods. The initial surface area of the plates and the rods was 1.12 cm^2^ and 4.42 cm^2^, respectively. The initial volume of the plates and rods was 0.044 cm^3^ and 0.663 cm^3^, respectively. The initial surface area and volume were calculated based on the initial dimensions of the plate and rod samples, respectively. The initial mass of the nHA_100, nHA_400, mHA_100, mHA_400 and non-coated Mg plates was measured to be 88.5 ± 0.7 mg, 88.7 ± 1.3 mg, 89.6 ± 0.9 mg, 90.5 ± 1.4 mg and 87.4 ± 0.3 mg, respectively, whereas the initial mass of the nHA_100, nHA_400, mHA_100, mHA_400 and non-coated Mg rods was measured to be 1188.7 ± 0.6 mg, 1186.3 ± 4.2 mg, 1193.6 ± 6.6 mg, 1193.2 ± 8.4 mg and 1187.6 ± 11.2 mg, respectively.

### Mechanical testing of nHA and mHA coated Mg rods before and after *in vitro* degradation in rSBF

The compressive strength of different Mg-based rods, i.e., nHA_100, nHA_400, mHA_100, mHA_400, and Mg control with a dimension of 7.5 mm × 15 mm, was obtained using a mechanical testing system (Insrton 5969, Norwood, MA). Specifically, a constant strain rate of 1 mm/min was applied on the samples, and loading was stopped at the final strain displacement of 6 mm. The stress and strain were calculated based on the loading force and displacement, and the stress-strain curve was plotted for each sample. The compressive strength of Mg rods (7.5 mm × 15 mm) after 6 weeks of immersion in rSBF was measured using the same Instron 5969 as described above. The ultimate compressive strength and maximum load for each sample was determined and compared before and after 6 weeks of immersion based on the respective stress-strain curves.

### *In vitro* direct culture of nHA and mHA coated Mg and non-coated Mg plates with bone marrow derived mesenchymal stem cells (BMSCs)

Bone marrow derived mesenchymal stem cells (BMSCs) were extracted from the femur and tibia of juvenile Sprague Dawley rats according to the established protocol^10^, which was approved by the Institutional Animal Care and Use Committee (IACUC) at the University of California, Riverside. The methods for BMSC harvesting were carried out in accordance with the recommendations in the Guide for the Care and Use of Laboratory Animal of the National Institutes of Health (NIH).

BMSCs were cultured in Dulbecco’s modified Eagle medium (DMEM; Corning) supplemented with 10 vol. % fetal bovine serum (FBS; Hyclone) and 1 vol. % penicillin/streptomycin (P/S; Hyclone). Hereafter, DMEM refers to DMEM with 10% FBS and 1% P/S. BMSCs at the second or third passages were used for *in vitro* experiments with nHA_400, mHA_400 and non-coated Mg plates. The nHA_400 and mHA_400 samples were selected for this *in vitro* cell study because they showed better coating preservation than the respective nHA_100 and mHA_100 samples after immersion degradation. Non-coated Mg served as the control. The nHA_400, mHA_400, and non-coated Mg plates were disinfected by exposing each side under ultraviolet (UV) radiation for an hour. The initial mass of each sample was recorded before the UV disinfection. The disinfected samples were first placed into 12-well tissue culture plates, with one sample per well. Each well was rinsed with 1 mL of DMEM to equilibrate osmotic pressure prior to the *in vitro* culture experiment. BMSCs were collected and seeded directly into each culture well at a density of 10,000 cells/cm^2^. Cells without any sample (BMSCs only) and blank media without any sample and cell (DMEM) were included as the culture controls. After 24 hours of culture, the media were collected from each well for measuring pH, Mg^2+^ ion concentrations, and Ca^2+^ ion concentrations, similarly as described above for the immersion degradation study. BMSCs on the surface of samples (direct contact) and on the well plates surrounding the respective samples (indirect contact) were fixed in 4% paraformaldehyde (10% neutral buffered formalin; VMR, Radnor, PA, USA) for 20 minutes, stained with Alex Fluor 488 Phalloidin (Life Technologies, Carlsbad, CA) for another 20 minutes to visualize F-actin, and finally stained with 4′, 6-diamidino-2-phenylindole (DAPI; Invitrogen) for 5 minutes to visualize cell nuclei. After fixation and staining, BMSCs were observed and imaged using a fluorescence microscope (Eclipse T*i* with NIS 241 software, Nikon, Melville, NY, USA) for analyses of cell morphology and cell adhesion density. Five images were taken on random areas of each sample for direct contact, and five images were taken on random areas of each well plate for indirect contract. The number of cells in each image was quantified using ImageJ. The cell adhesion density under direct contact and indirect contact conditions was calculated as the number of cells per unit area. Each type of sample was run in triplicate in this *in vitro* cell study for repeatability and statistical analyses.

### Statistical analysis

One-way analysis of variance (ANOVA) was applied to the numerical data to determine the statistical differences among the groups of interest. Tukey post-hoc test was used for detecting statistical differences when comparing two different groups. Statistical differences were considered at *p < 0.05, **p < 0.01, ***p < 0.001, ****p < 0.0001.

## Results

### Characterization of nHA and mHA coatings on Mg substrates after the TPA process and the corresponding nHA and mHA powders used for the TPA process

**Figure 1:**
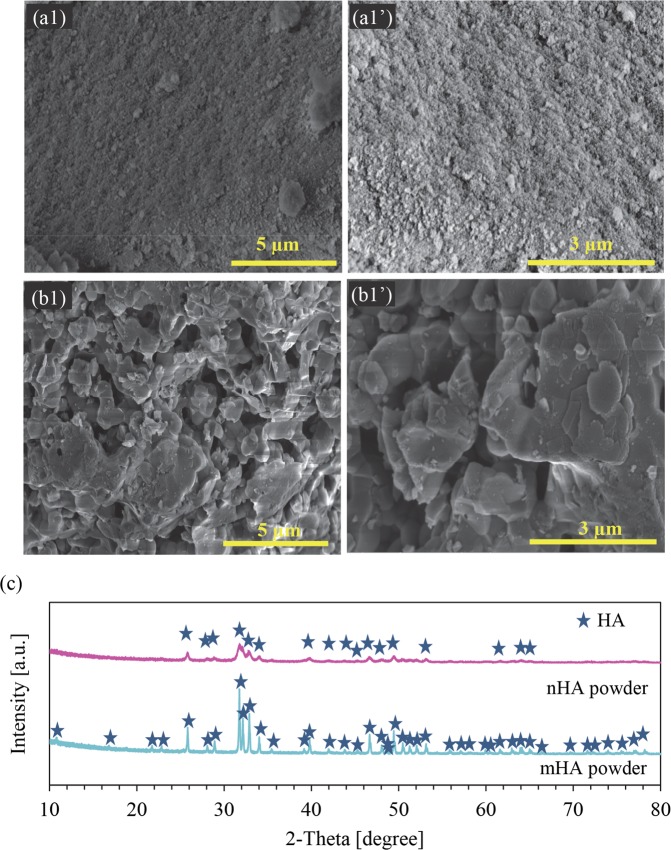
Surface microstructures and phase analyses of nHA and mHA powders. (**a1**,**a1’**,**b1**,**b1’**) SEM images of (**a1**,**a1’**) nHA and (**b1**,**b1’**) mHA powders. The original magnification was 10,000x for (**a1**,**b1**) and 20,000x for (**a1’**,**b1’**). Scale bar = 5μm for (**a1**,**b1**) and Scale bar = 3μm for (**a1’**,**b1’**). (**c**) XRD spectra of nHA and mHA powders. Phases were identified based on nHA (ICSD pattern 00-001-1008) and mHA (ICSD pattern 01-073-8419).

**Figure 2:**
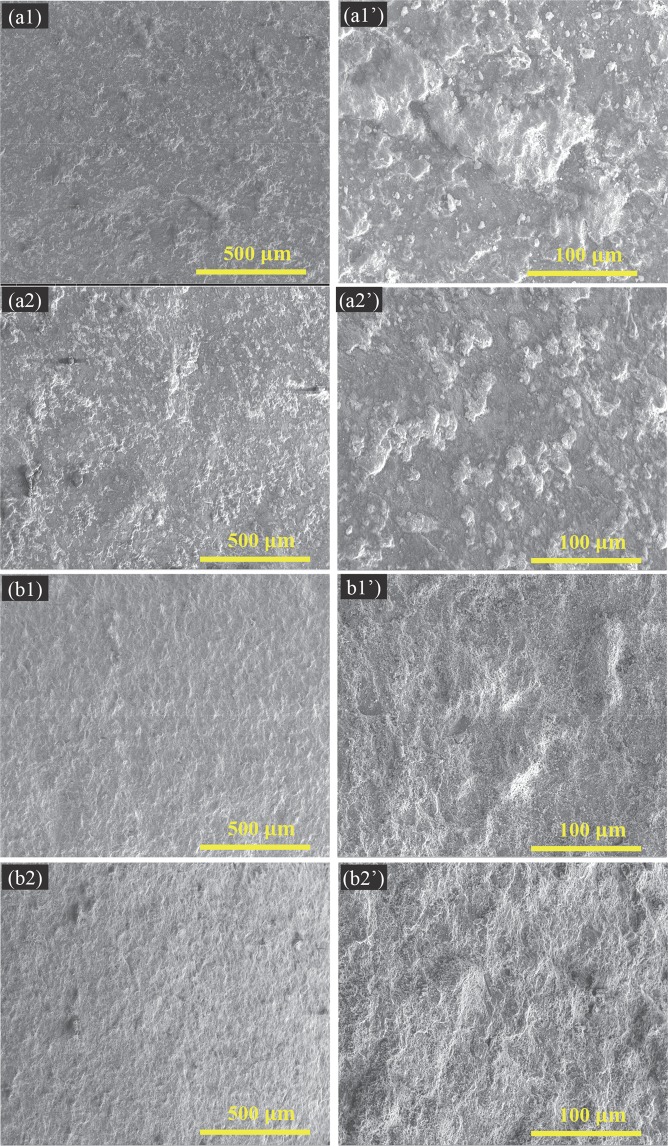
Surface microstructures of nHA_100, nHA_400, mHA_100, and mHA_400 coated Mg rods, as well as non-coated Mg rod control under low magnification. Mg rod samples had an initial dimension of 7.5 mm in diameter and 15 mm in height. SEM images of (**a1**,**a1’**) nHA_100, (**a2**,**a2’**) nHA_400, (**b1**,**b1’**) mHA_100, (**b2**,**b2’**) mHA_400. The original magnification was 100x for (**a1**,**a2**,**b1**,**b2**) and 500x for (**a1’**,**a2’**,**b1’**,**b2’**). Scale bar = 500 µm for (**a1**,**a2**,**b1**,**b2**) and scale bar = 100 µm for (**a1’**,**a2’**,**b1’**,**b2’**).

**Figure 3:**
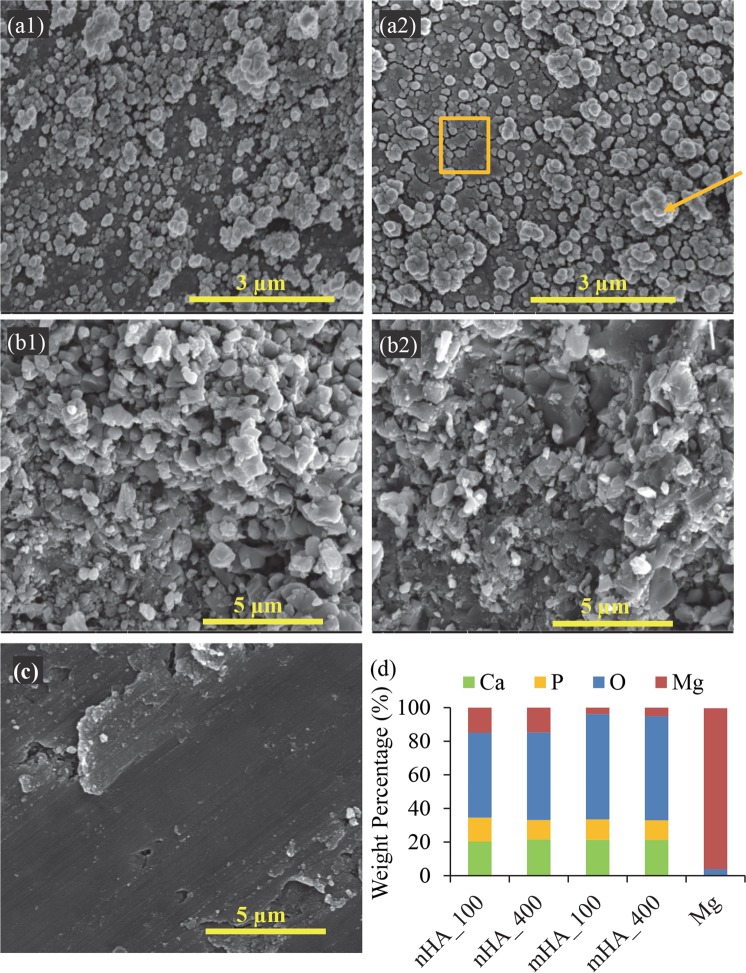
Surface microstructures and elemental compositions of nHA_100, nHA_400, mHA_100, and mHA_400 coated Mg rods, as well as non-coated Mg rod control under high magnification. Mg rod samples had an initial dimension of 7.5 mm in diameter and 15 mm in height. SEM images of (**a1**) nHA_100, (**a2**) nHA_400, (**b1**) mHA_100, (**b2**) mHA_400 and (**c**) Mg. The original magnification for nHA was 20,000x and for mHA and non-coated Mg was 10,000x. Orange square in (**a2**) indicates the first-deposited layer of nHA particles that were flattened onto the substrates by TPA process and orange arrow in (**a2**) indicates the subsequently-deposited layer of nHA particles. Scale bar = 3 µm for (**a1**,**a2**) and scale bar = 5 µm for (**b1**,**b2**,**c**). (**d**) Surface elemental composition (wt%) for each sample, quantified on respective SEM images of a-c using EDS.

The microstructures of nHA and mHA powders were characterized using SEM, as shown in Fig. 1. The microstructures of nHA_100, nHA_400, mHA_100, and mHA_400 coated Mg, as well as non-coated Mg control were characterized using SEM and EDS, as shown in Figs 2 and 3. Figure 2 shows the lower-magnification (100x and 500x) SEM images of the nHA_100, nHA_400, mHA_100, and mHA 400 coated Mg for overview of the coating morphologies. Figure 3 shows the higher-magnification (10000x and 20000x) SEM images of the respective samples and the nanostructures of the coatings. The nHA_100, nHA_400, mHA_100, and mHA_400 coatings all showed a full coverage of HA on the Mg substrates. The nHA powder used for the TPA process (Fig. 1a1’) appeared to have smaller particle size and more aggregated morphology than the nHA coating on Mg after TPA process (Fig. 3a1,a2). In contrast, mHA coating on Mg after the TPA process (Fig. 3b1,b2) showed visually finer microstructures and less aggregates than the mHA powder used for the TPA process, as shown in Fig. 1(b1). XRD analyses showed the HA [Ca_5_(PO_4_)_3_(OH)] phase for both nHA and mHA powders before the TPA process in Fig. 1(c). In the XRD spectra, the nHA powder showed less peaks with lower intensity than that of the mHA powder.

The nHA_100 and nHA_400 coatings showed two distinctive features as highlighted in Fig. 3(a2) by an orange square and an orange arrow, repsectively. The former feature as shown in the orange square seemed that spherical-like HA particles were embedded and compressed onto the substrates, possibly because of the high TPA pressure applied to the initially deposited nHA particles. The latter feature as pointed out by the orange arrow appeared to be the nHA particles that were deposited onto the substrates subsequently. The mHA_100 and mHA_400 coatings seemed similar to each other, but drastically different from the two nHA coatings of nHA_100 and nHA_400. Mg control without coating showed a machined surface with several defects and cracks, possibly from the manufacturing process (Fig. 3c). The EDS analyses in Fig. 3d confirmed the presence of Ca, P and O in nHA_100, nHA_400, mHA_100 and mHA_400 coated Mg, which are the expected elements for HA [Ca_5_(PO_4_)_3_(OH)]. As expected, the elemental compositions of the nHA_100 and nHA_400 coated Mg were very similar to each other, and those of the mHA_100 and mHA_400 coated Mg were similar to each other as well. All the coated samples showed a small percentage of Mg; interestingly, the nHA coatings showed a much higher Mg content than that in the mHA coatings. For Mg control, Mg was the major peak detected, with a very small amount of O, most likely from residual oxides left on the surface after the manufacturing process and sample preparation.

**Figure 4:**
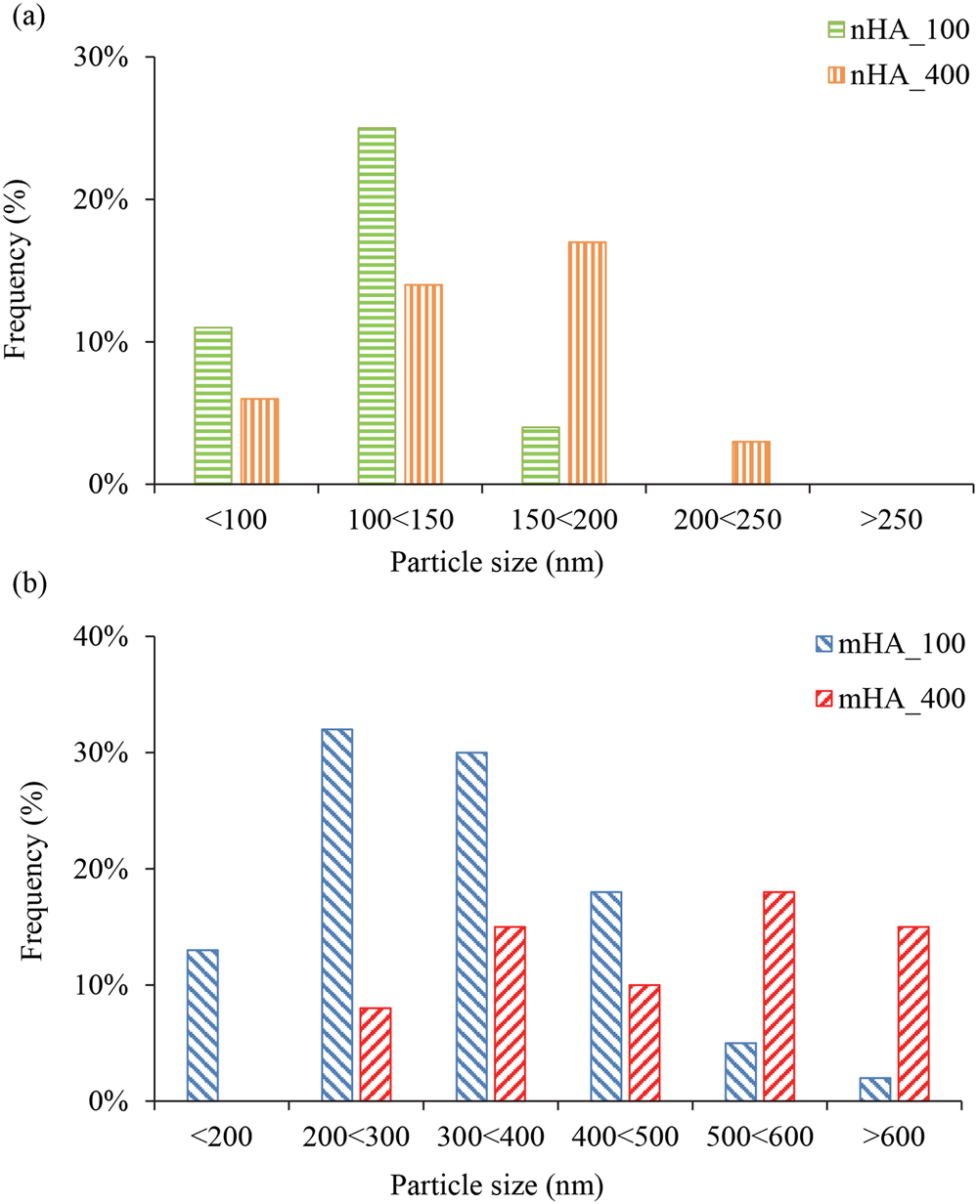
Size distributions of HA in the coatings of (**a**) nHA_100 and nHA_400, and (**b**) mHA_100 and mHA_400 on Mg substrates after TPA deposition. Particle size in diameter is in nanometer (nm).

Quantitative analyses of the microstructures of nHA and mHA coatings in Fig. 4 showed that the nHA coating generally had a finer microstructure (i.e. smaller particle size) than those in mHA coating; and the coating formed under higher TPA pressure, i.e., nHA_400 and mHA_400, showed a coarser microstructure (i.e., larger particle size) than their counterparts formed under lower TPA pressure, i.e., nHA_100 and mHA_100 respectively. Specifically, the size of HA was less than 200 nm in nHA_100, with a dominant percentage of 25% stayed in the range of 100 nm to 150 nm, as shown in Fig. 4a. For nHA_400, the size of HA was less than 250 nm with a dominant percentage of 18% in the range of 150 nm to 200 nm. For mHA_100 coating in Fig. 4b, a dominant percentage of 30% of HA particles had a diameter from 200 nm to 300 nm with 2% features greater than 600 nm. For mHA_400 coating, a dominant percentage of 18% of HA particles had a diameter from 500 nm to 600 nm with 15% features greater than 600 nm.

**Figure 5:**
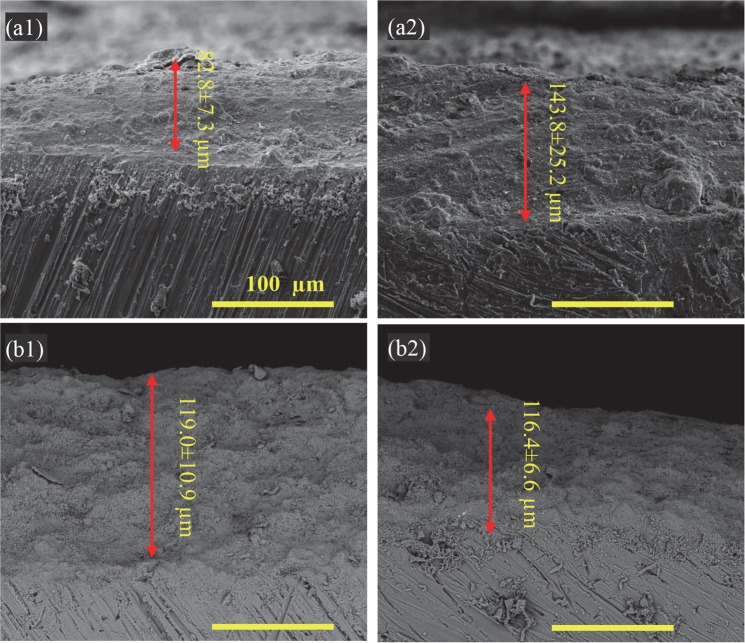
SEM images of the cross sections of (**a1**) nHA_100, (**a2**) nHA_400, (**b1**) mHA_100, and (**b2**) mHA_400 coatings on Mg substrates after TPA deposition. The coating thickness was 82.8 ± 7.3 μm for nHA_100, 143.8 ± 25.2 μm for nHA_400, 119.0 ± 10.9 μm for mHA_100, and 116.4 ± 6.6 μm for mHA_400. The original magnifications were 500x for all images.

The SEM images of cross sections of nHA_100, nHA_400, mHA_100, and mHA_400 coated Mg in Fig. 5 showed the coating thickness and standard deviation. All the coatings were densely packed without any obvious cracks or voids at an original magnification of 5,000x. The nHA_100, nHA_400, mHA_100 and mHA_400 coatings had a thickness of 82.8 ± 7.3 µm, 143.8 ± 25.2 µm, 119.0 ± 10.9 µm and 116.4 ± 6.6 µm, respectively.

### Qualitative characterization of Mg plates and Mg rods after immersion degradation in rSBF

**Figure 6:**
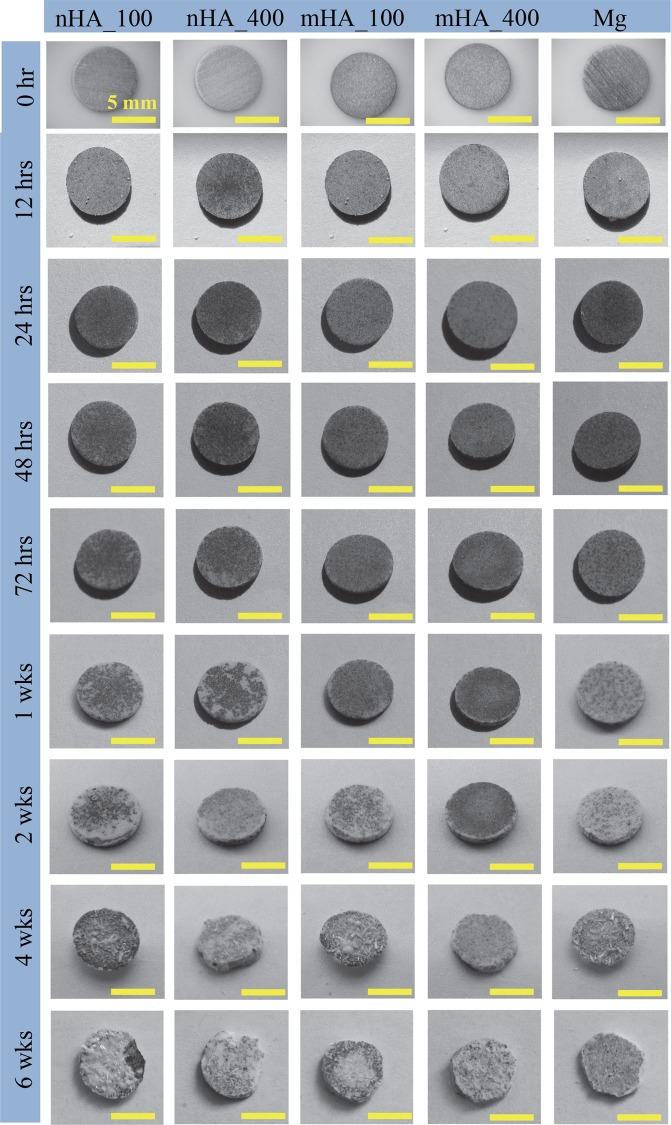
Macroscopic images of nHA_100, nHA_400, mHA_100, mHA_400 coated Mg plates as well as non-coated Mg plate control at each prescribed time point during 6 weeks of immersion degradation in rSBF. Mg plates had a starting dimension of 7.5 mm in diameter and 1 mm in thickness. The plate samples before immersion is show in the 0 hr row.

Figure 6 shows the macroscopic images of Mg plates with a diameter and thickness of 7.5 mm × 1 mm, including nHA_100, nHA_400, mHA_100, and mHA_400 coated Mg, as well as non-coated Mg control, at each prescribed time point during the 6 weeks of immersion in rSBF. All the samples showed continuous deposition of degradation products on the surface. Small crystal features started to form on the surface of all the samples at 1 week and became visible to human eyes. As the immersion continued, more crystals formed and grew larger on all the sample surface, but in different rates. The nHA_100 showed the most accumulation of the crystals after 6 weeks of immersion among all the samples. At 2 weeks of immersion, cracks were found on the edge of nHA_100 coated Mg sample, but not for the other samples. At 4 weeks of immersion, cracks were found on the edge of all the samples. At the end of 6 weeks of immersion, all the samples lost their regular shape, but mHA_400 retained the structural integrity the most.

**Figure 7:**
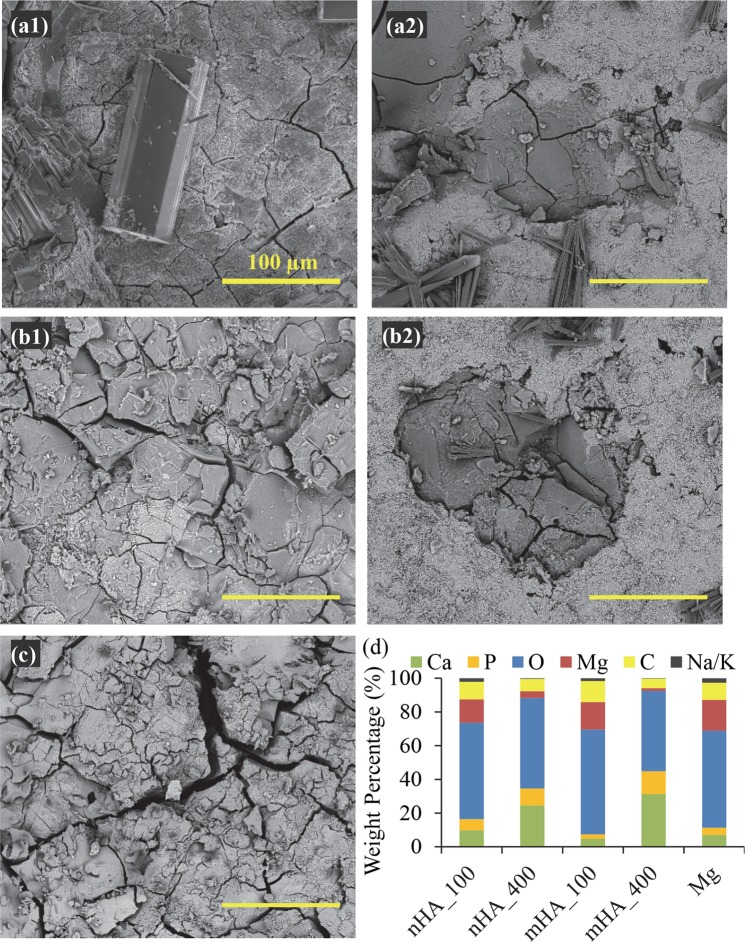
Surface microstructures and elemental compositions of nHA_100, nHA_400, mHA_100, and mHA_400 coated Mg plates, as well as non-coated Mg plate control after immersion in rSBF for 6 weeks. SEM images of (**a1**) nHA_100, (**a2**) nHA_400, (**b1**) mHA_100, and (**b2**) mHA_400 coated Mg, and (**c**) non-coated Mg. The original magnifications for all images were 500x. (**d**) Surface elemental compositions (wt %) of each sample after immersion in rSBF for 6 weeks, quantified on the respective SEM images of a–c using EDS analyses. All Mg plate samples had an initial dimension of 7.5 mm in diameter and 1 mm in height.

Figure 7 shows the SEM images of Mg plates after 6 weeks of immersion in rSBF, which had a very different surface morphology from the respective samples before the immersion study as shown in Fig. 2(a1’,a2’). All the Mg-based samples showed cracks after 6 weeks of immersion, and Mg control showed the most and the largest cracks among all the samples. Crystal deposition was found on the surface of nHA_100, nHA_400 and mHA_400, where the nHA_100 had the largest crystals. In nHA_400 and mHA_400 (Fig. 7a2,b2), a white layer composed of small particles (most likely the original HA coating) covered a large surface area of the samples. The EDS analyses confirmed the presence of Ca, P, O, Mg and C in all the Mg-based samples. Interestingly, nHA_400 and mHA_400 had a higher content of Ca and P and lower percentage of Mg than all the other samples, which indicated higher Ca/P deposition or better preservation of HA coating during the immersion. When comparing between nHA_400 and mHA_400, mHA_400 showed the highest content of Ca and P and lowest Mg. Trace elements such as sodium (Na) and potassium (K), were also found on all the Mg-based samples after 6 weeks of immersion.

**Figure 8:**
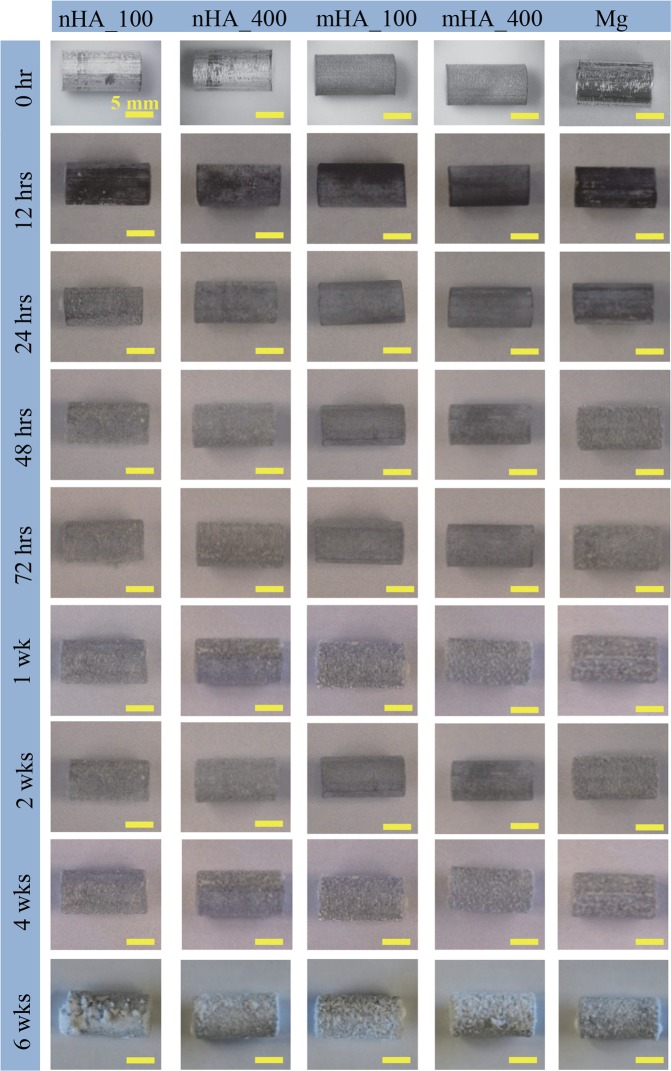
Macroscopic images of nHA_100, nHA_400, mHA_100, and mHA_400 coated Mg rods, as well as non-coated Mg rod as a control at each prescribed time point during 6 weeks of immersion degradation in rSBF. All Mg rod samples had an initial dimension of 7.5 mm in diameter and 15 mm in height before immersion in rSBF. The rod samples before immersion are shown in the 0 hr row.

**Figure 9:**
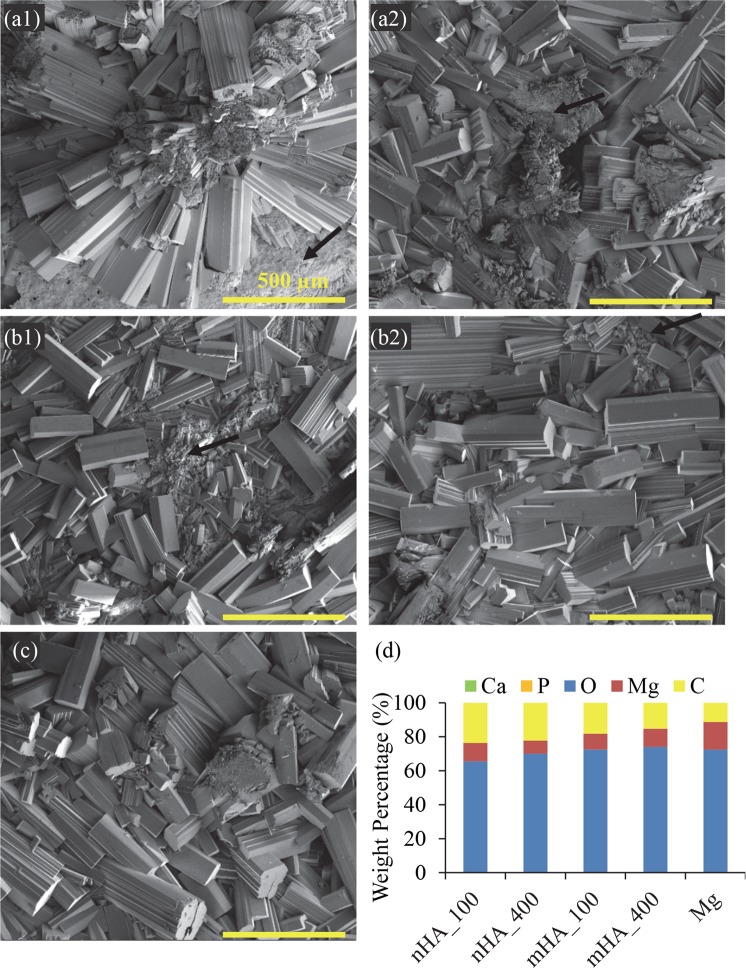
Surface microstructures and elemental compositions of nHA_100, nHA_400, mHA_100, and mHA_400 coated Mg rods, as well as non-coated Mg rod control after immersion in rSBF for 6 weeks. SEM images of (**a1**) nHA_100, (**a2**) nHA_400, (**b1**) mHA_100, and (**b2**) mHA_400 coated Mg rods, and (**c**) non-coated Mg rod. The original magnifications for all images were 100x. (d) Surface elemental compositions (wt %) of each sample after immersion in rSBF for 6 weeks, quantified on the respective SEM images of a-c using EDS analyses. All Mg rod samples had an initial dimension of 7.5 mm in diameter and 15 mm in height.

Figure 8 shows the macroscopic images of Mg rods with a diameter and height of 7.5 mm × 15 mm, i.e., nHA_100, nHA_400, mHA_100, and mHA_400 coated Mg, as well as Mg control, at each prescribed time point during the 6 weeks of immersion in rSBF. All the samples showed continuous deposition of degradation products on the surface. During immersion, small crystal features started to form on the surface of all the samples at 72 hours, and the crystals became larger and accumulated more as the immersion continued. No obvious cracks were observed for all the Mg rod samples except nHA_100 coated Mg rod and Mg control during the 6 weeks of immersion. SEM images in Fig. 9 shows the surface microstructures of Mg rods after 6 weeks of immersion, which had very different surface morphology from the respective samples before immersion as shown in Fig. 2(b1,b2). Aggregation of crystals was observed on all the Mg rod samples after 6 weeks in rSBF, which covered most surfaces of all the samples, except a few spots of remaining HA coating as indicated by black arrows in Fig. 9. The EDS analyses in Fig. 9d confirmed the presence of Ca, P, O, Mg and C in all the Mg-based samples, but the percentages of Ca and P were low (<2% in weight). C content on Mg rod samples was significantly higher than that on their respective Mg plate counterparts (Fig. 7d), possibly because significantly more prism-shaped crystals formed on Mg rod samples.

**Figure 10:**
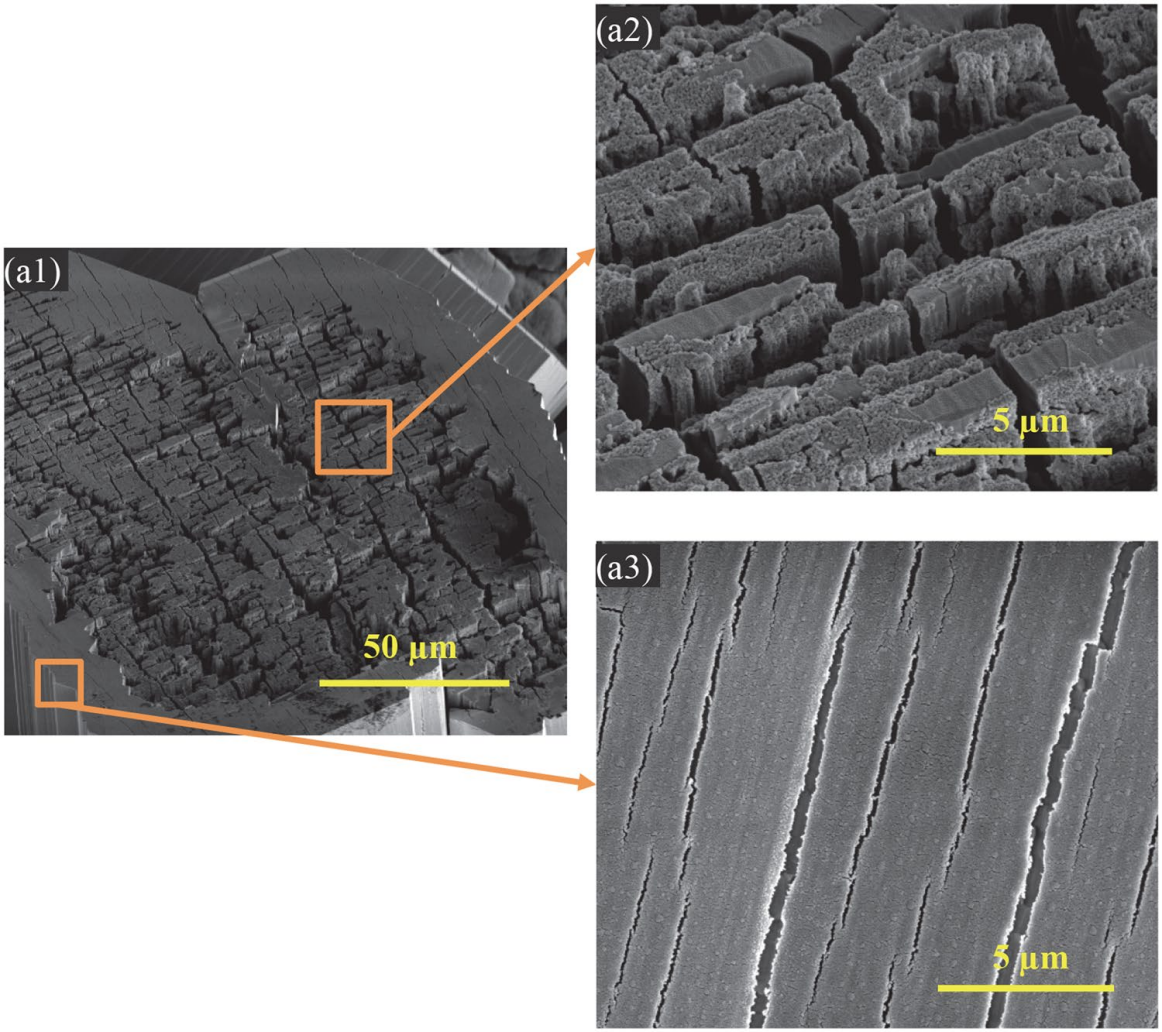
Representative SEM images of the features found in the degradation layers of Mg rods after immersion in rSBF for 6 weeks. (**a1**) Overview of the canyon-like region at an original magnification of 1,000x. (**a2**) SEM image of the magnified canyon-like region at an original high magnification of 10,000x. (**a3**) SEM image of the region surrounding the canyon-like region at an original high magnification of 10,000x.

The degradation products formed on both the coated and non-coated Mg plates and Mg rods. Mg rod samples showed more and larger crystals on their surfaces, and their microstructures and morphologies were further characterized, as shown in the higher-magnification SEM images in Fig. 10. Figure 10(a1) shows the surface morphologies of the canyon-like region and the surrounding flat region. Figure 10(a2) shows the vertical layers of canyon-like microstructures at the higher magnification that aligned in parallel. Nano-sized spherical particles were observed on top of the canyons and between the vertical canyons. Figure 10(a3) shows the flat region peripheral to the canyon-like region, which had cracks aligned in a nearly parallel fashion and spherical-shaped nano-scale features on the surface, likely the precursors for the canyon formation. In general, the results suggested that the canyon-like microstructures formed because of Mg degradation and deposition of nano-sized spherical particles.

**Figure 11:**
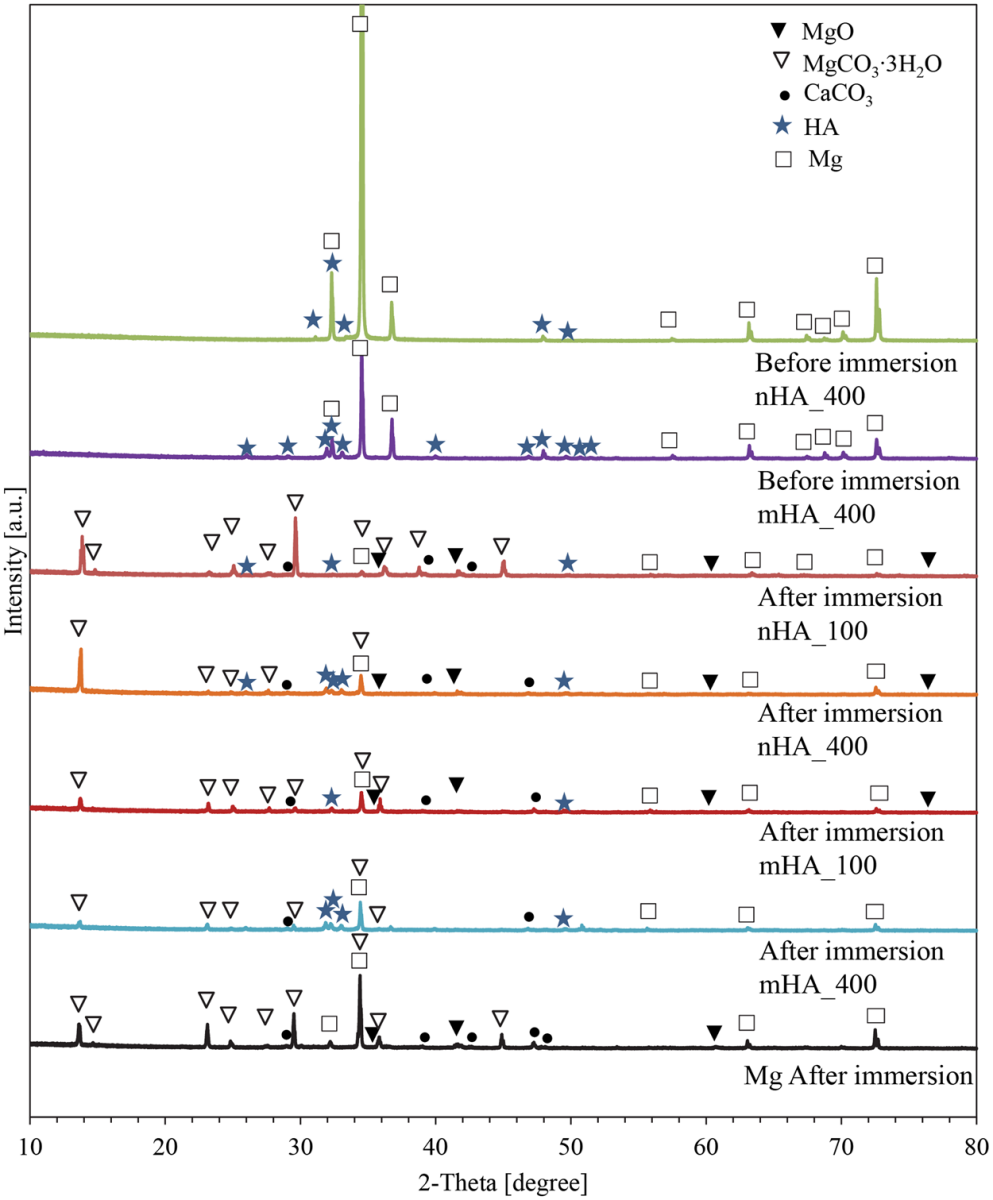
XRD spectra of degradation products on the surface of nHA_100, nHA_400, mHA_100, and mHA_400 coated Mg plates, as well as non-coated Mg plate control after immersion in rSBF for 6 weeks. XRD spetra of the representative nHA and mHA coatings before immersion were included for comparison. (**a**) nHA_100 and (**b**) nHA_400 after immersion, (**c**) nHA_400 before immersion, (**d**) mHA_100 and (**e**) mHA_400 after immersion, (**f**) mHA_400 before immersion, and (**g**) Mg plate after immersion. Mg plate samples had a dimension of 7.5 mm × 1 mm in diameter and thickness. Phases were identified based on MgO (ICSD pattern 01-077-2906), MgCO_3_∙3H_2_O (ICSD pattern 00-020-0669), CaCO_3_ (ICSD pattern 01-086-0174), HA (ICSD pattern 01-075-9526) and Mg (ICSD pattern 03-065-3365).

**Figure 12:**
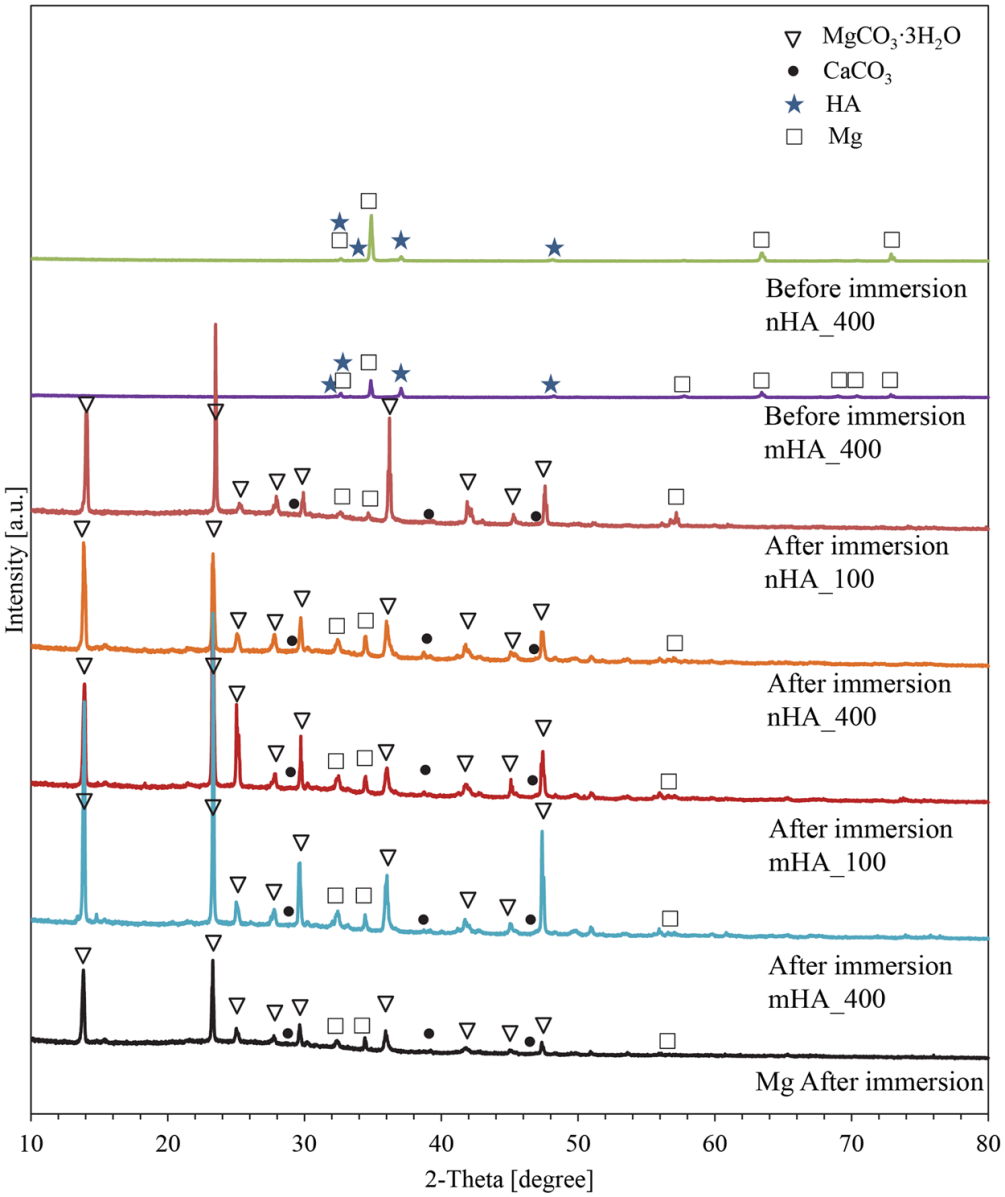
XRD spectra of degradation products on the surface of nHA_100, nHA_400, mHA_100, and mHA_400 coated Mg rods, as well as non-coated Mg rod control after immersion in rSBF for 6 weeks. XRD spetra of the representative nHA and mHA coatings before immersion were included for comparison. The XRD spectra are for the samples of nHA_400 and mHA_400 before immersion, and nHA_100, nHA_400, mHA_100, mHA_400 and Mg control after immersion. Mg rod samples had an initial dimension of 7.5 mm in diameter and 15 mm in height. Phases were identified based on MgCO_3_∙3H_2_O (ICSD pattern 00-020-0669), CaCO_3_ (ICSD pattern 01-086-0174), HA (ICSD pattern 00-001-1008) and Mg (ICSD pattern 01-079-6692).

**Figure 13:**
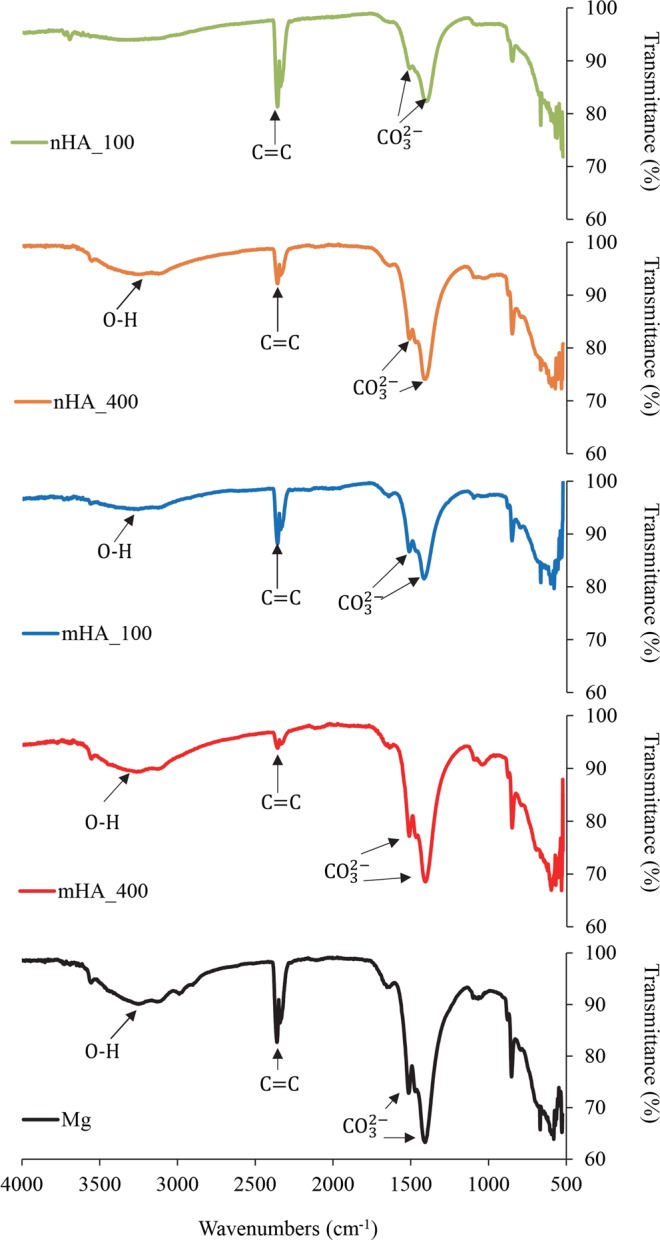
FTIR spectra of the degradation layers on the surface of nHA_100, nHA_400, mHA_100 mHA_100, and mHA_400 coated Mg rods, and non-coated Mg rod after immersion in rSBF for 6 weeks. Mg rod samples had an initial dimension of 7.5 mm in diameter and 15 mm in height.

XRD analysis in Fig. 11 showed the phases of Mg, MgO, MgCO_3_∙3H_2_O, CaCO_3_, and hydroxyapatite (HA) on nHA and mHA coated or non-coated Mg plates after 6 weeks of immersion. The nHA_400 and mHA_400 coated plates before the immersion study was included as a representative sample for nHA and mHA coatings, and the XRD analyses of those two samples before immersion showed the presence of only Mg and hydroxyapatite (HA) phases, as shown in Fig. 11. As expected in Fig. 12, nHA_400 and mHA_400 coated rods before the immersion study also showed the presence of only Mg and hydroxyapatite (HA) phases. After 6 weeks of immersion, XRD spectra of the nHA and mHA coated and non-coated Mg plates and rods confirmed that the major peaks matched the reference of MgCO_3_∙3H_2_O (Figs 11 and 12). Other minor peaks also suggested the presence of CaCO_3_, which was consistent with the Ca content found in the EDS results (Figs 7 and 9). FTIR confirmed the presence of carbonates (Fig. 13) on Mg rod samples. Specifically, O-H, C=C, and CO_3_^2−^ functional groups were identified in the FTIR spectra (Fig. 13), which indicated the presence of MgCO_3_∙3H_2_O and CaCO_3_. All samples showed similar XRD spectra and FTIR spectra after 6 week of immersion in rSBF, which suggested a consistent formation of highly crystallized MgCO_3_∙3H_2_O phase on the surfaces of all Mg rod samples.

### Quantitative degradation analyses of Mg plates and Mg rods in rSBF

**Figure 14:**
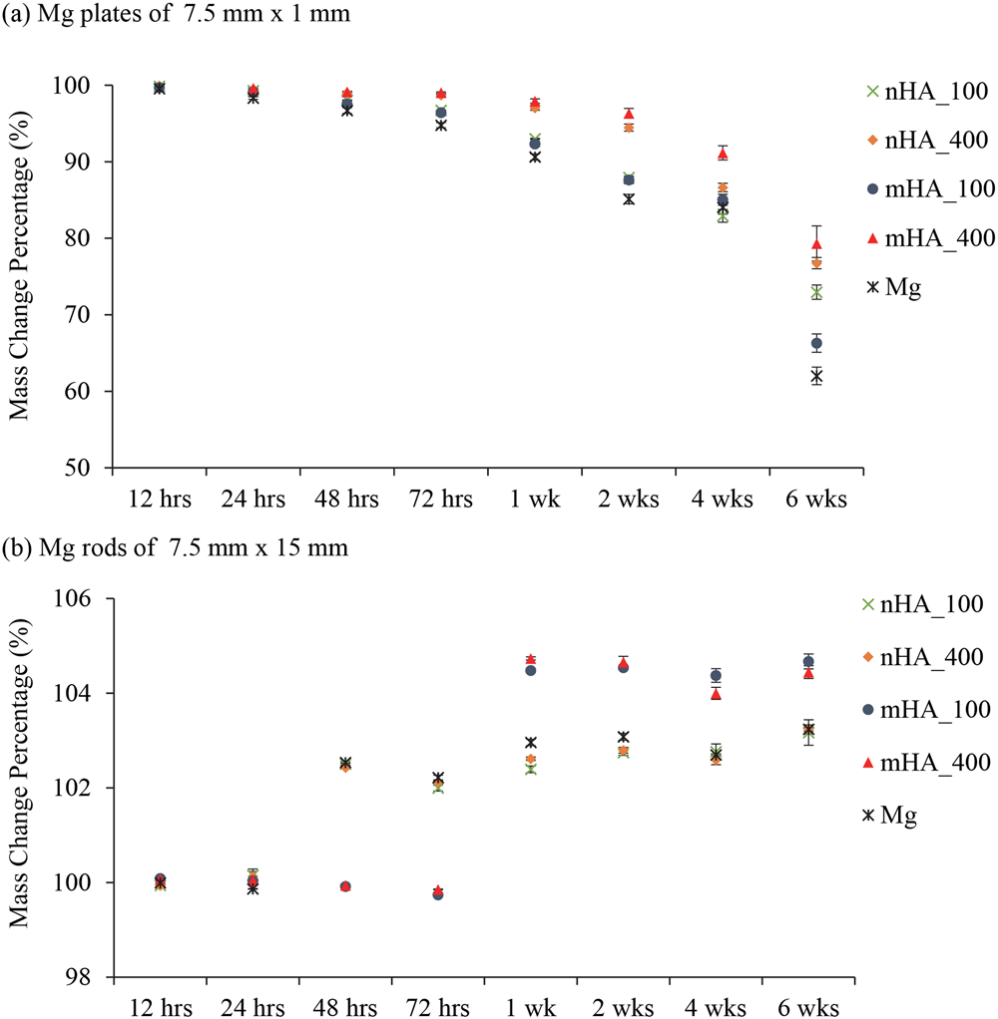
The mass change (final/initial) of nHA_100, nHA_400, mHA_100, mHA_400 coated Mg plates or rods, and non-coated Mg plate or rod control at each prescribed time point during the immersion in rSBF for 6 weeks. (**a**) Mass change of Mg plate samples with an initial dimension of 7.5 mm in diameter and 1 mm in height. (**b**) Mass change of Mg rod samples with an initial dimension of 7.5 mm in diameter and 15 mm in height. Data are mean ± standard error (N = 3).

During the 6-week immersion, the mass changes of Mg plates of 7.5 mm × 1 mm are summarized in Fig. 14a, and the mass changes of Mg rods of 7.5 mm × 15 mm are summarized in Fig. 14b. For Mg plates in Fig. 14a, all the samples showed a decrease of mass starting at 24 hours. The nHA_400 and mHA_400 coated Mg plates showed the least mass loss among all the samples, followed by nHA_100 and mHA_400 after 6 week of immersion. Mg control had the most mass loss with only 60% mass remaining at the end of 6 weeks. For Mg rods in Fig. 14b, continuous deposition of degradation products increased the mass of all the samples by 4% in average after 6 weeks of immersion. After 24 hours, nHA_100, nHA_400 and non-coated Mg rods had insignificant mass change as compared with their respective initial mass. After 48 hours, 2% to 3% mass increase was observed on nHA_100 and nHA_400 coated and non-coated Mg rods as compared with their respective mass at 12 and 24 hours, while mHA_100 and mHA_400 coated Mg rods showed 4% to 5% mass increase after 1 week, as compared with their respective mass at the early time points of 12, 24, 48 and 72 hours. After 1 week of immersion, all the Mg rod samples showed significant mass increase, where mHA_100 and mHA_400 coated rods had more mass gain than the nHA_100 and nHA_400 coated rods and non-coated Mg rod control.

**Figure 15:**
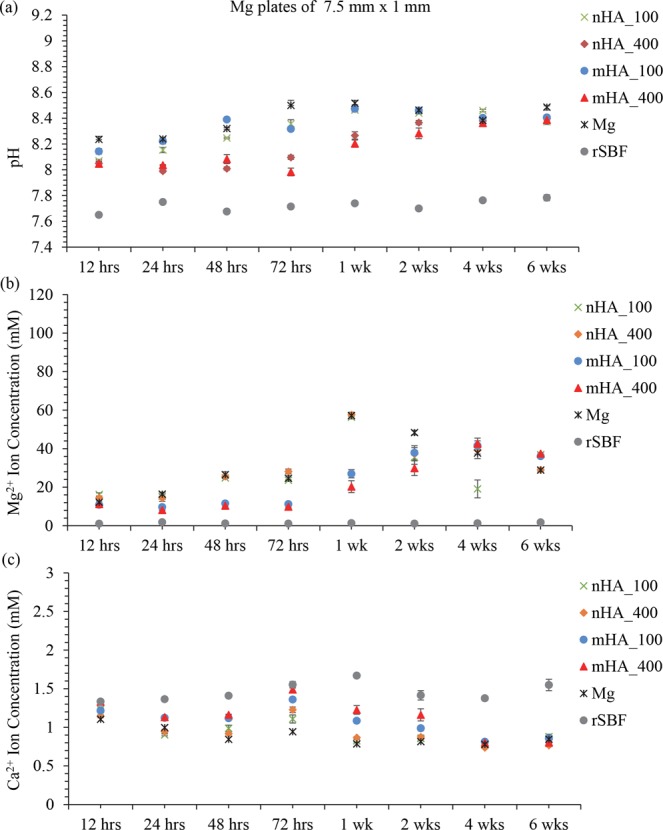
Post-culture media analyses at each prescribed time point after immersion of nHA_100, nHA_400, mHA_100, and mHA_400 coated Mg plates, non-coated Mg plate control in rSBF, and rSBF control for 6 weeks. Mg plate samples had a dimension of 7.5 mm in diameter and 1 mm in thickness. (**a**) The pH of the collected rSBF after cultured with each sample at each prescribed time point. (**b**) The Mg^2+^ ion concentration in the collected rSBF after cultured with each sample at each prescribed time point. (**c**) The Ca^2+^ ion concentration in the collected rSBF after cultured with each sample at each prescribed time point. Data are mean ± standard error (N = 3).

Figure 15a shows the pH of the media after being cultured with Mg-based plates of 7.5 mm × 1 mm. In comparison with the pH of rSBF, ANOVA confirmed a significant increase of pH for the groups with Mg-based samples at all the time points during 6 weeks of immersion. At the early time points of 12 hours, 48 hours, 72 hours and 1 week, a significant increase in the pH of the rSBF during the immersion with the non-coated Mg plates was found when compared with the pH of the nHA_400 and mHA_400 groups. In general, mHA_400 coated Mg plates showed the least pH increase among all the plate samples after 72 hours. After 2 weeks, all the groups with Mg-based plate samples showed a similar pH increase with no significant difference. Figure 15b shows Mg^2+^ ion concentrations in the media after being cultured with Mg-based plates during 6 weeks of immersion in rSBF. When compared with the Mg^2+^ ion concentrations in the rSBF reference, ANOVA showed a significant increase in Mg^2+^ ion concentrations in the rSBF cultured with Mg-based plate samples at most of the time points except for 24 hours and 48 hours. At 48 hours and 1 week, mHA coated Mg plates showed significantly lower Mg^2+^ ion concentrations than non-coated Mg, whereas mHA_400 coated Mg plates showed significantly lower Mg^2+^ ion concentrations than nHA coated Mg plates at 72 hours. In general, mHA_400 showed the least increase in Mg^2+^ ion concentration among all the plate samples in the most time points until 2 weeks. Interestingly, nHA_100 and nHA_400 coated and non-coated Mg plates showed that Mg^2+^ ion concentration reached a peak at 1 week. At 2 weeks, all the HA coated Mg plate samples showed a similar increase in Mg^2+^ ion concentration without statistically significant differences among them, but all lower than that of Mg plate control. After 4 weeks, all the HA coated Mg plate samples showed a similar increase in Mg^2+^ ion concentration without statistically significant differences. In contrast to pH and Mg^2+^ ion concentration, the groups of Mg-based plates in Fig. 15c all showed a decreasing Ca^2+^ ion concentration when compared with the Ca^2+^ ion concentration in the rSBF reference at most of the time points except for the initial 12 hours during 6 weeks of immersion. The nHA_100 and nHA_400 coated and non-coated Mg plates showed that their Ca^2+^ ion concentrations reached the lowest at 1 week of immersion. ANOVA confirmed a significant higher Ca^2+^ ion concentration in the rSBF cultured with the mHA_400 than that of nHA coated Mg plates, and a significant higher Ca^2+^ ion concentration in the rSBF cultured with the mHA_100 than that of nHA_100 coated Mg plates at 24 hours. At 48 hours, the Ca^2+^ ion concentrations for the groups of mHA coated Mg plates were significantly higher than that for the non-coated Mg; and at 72 hours, the Ca^2+^ ion concentration for the group of mHA_400 was significantly higher than that for the non-coated Mg. Generally, mHA_400 coated Mg plates showed the least decrease in Ca^2+^ ion concentration among all the plate samples until 2 weeks. After 4 weeks, all the Mg-based plate groups showed a similar decrease in Ca^2+^ ion concentration without statistically significant differences among the HA coated Mg plates. Interestingly, the increase of Mg^2+^ ion concentration in rSBF showed a correlation with the decrease of Ca^2+^ ion concentration in all the Mg-based plate samples at all the time points. That is, the greater the increase of Mg^2+^ ion concentration, the greater the decrease of Ca^2+^ ion concentration and vice versa.

**Figure 16:**
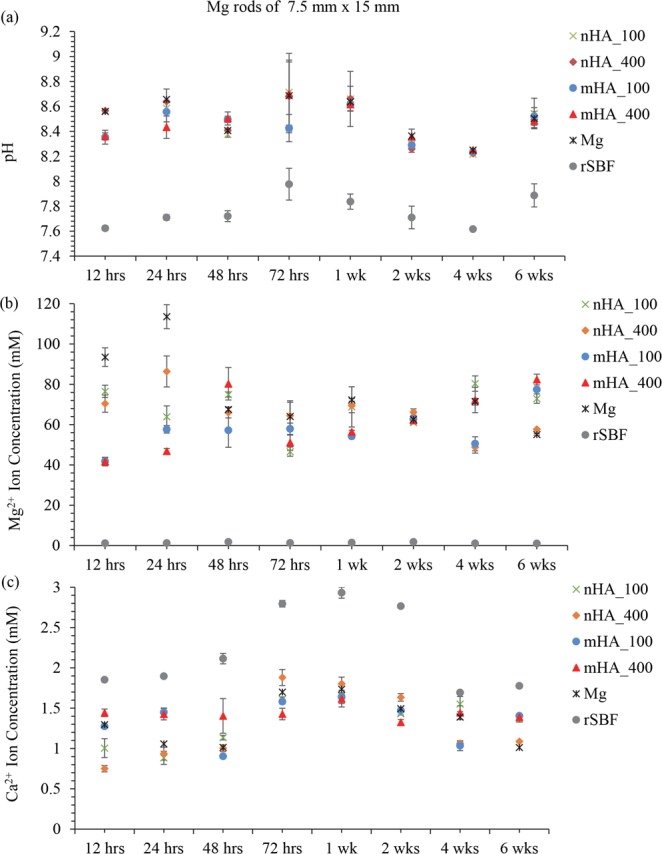
Post-culture media analyses at each prescribed time point after immersion of nHA_100, nHA_400, mHA_100, and mHA_400 coated Mg rods, non-coated Mg rod control in rSBF, and rSBF control for 6 weeks. Mg rod samples had a dimension of 7.5 mm in diameter and 15 mm in height. (**a**) The pH of the collected rSBF after cultured with each sample at each prescribed time point. (**b**) The Mg^2+^ ion concentration in the collected rSBF after cultured with each sample at each prescribed time point. (**c**) The Ca^2+^ ion concentration in the collected rSBF after cultured with each sample at each prescribed time point. Data are mean ± standard error (N = 3).

Figure 16a shows the pH of the media after being cultured with Mg-based rods of 7.5 mm × 15 mm. All the groups with Mg-based rods showed a significant increase of pH at each time point during 6 weeks of immersion when compared with the pH of the rSBF control. Among the groups with Mg-based rods, the mHA coated Mg rods showed significantly lower pH than that of the nHA coated Mg and non-coated Mg rods at 12 hours. For the time points from 12 hours to 6 weeks, the pH of rSBF did not show statistically significant differences for all the groups with Mg-based rods. Similarly, in Fig. 16b, all the groups with Mg-based rods showed a significant increase of Mg^2+^ ion concentration at each time point during 6 weeks of immersion when compared with the Mg^2+^ ion concentration in the rSBF control. Non-coated Mg rods showed a significantly greater Mg^2+^ ion concentration than that of mHA coated Mg rods at 12 hours and 24 hours. No significant differences were found among the coated and non-coated Mg rods from 48 hours to 4 weeks. At 6 weeks, nHA_400 coated Mg rods showed significantly lower Mg^2+^ ion concentration than nHA_100 and mHA-coated Mg rods. All the groups with Mg-based rods showed a significant decrease in Ca^2+^ ion concentrations in Fig. 16c when compared with the rSBF control at each time point during 6 weeks of immersion. Initially at 12 hours, the groups cultured with nHA coated Mg rods showed significantly lower Ca^2+^ ion concentrations than that of mHA coated Mg rods; non-coated Mg showed a significantly lower Ca^2+^ ion concentration than that of mHA_400 coated Mg rods. At 24 hours, the Ca^2+^ ion concentrations in the groups with nHA coated and non-coated Mg rods were significantly lower than that of the groups with mHA coated Mg rods. No significant differences were found among the Mg-based rods from 48 hours to 4 weeks. At 6 weeks, ANOVA confirmed a significantly lower Ca^2+^ ion concentration in the groups with nHA_400 coated and non-coated Mg rods than that in the groups with the mHA and nHA_100 coated Mg rods.

### *In vitro* daily Mg^2+^ release rates of the nHA and mHA coated Mg plates and rods

**Figure 17:**
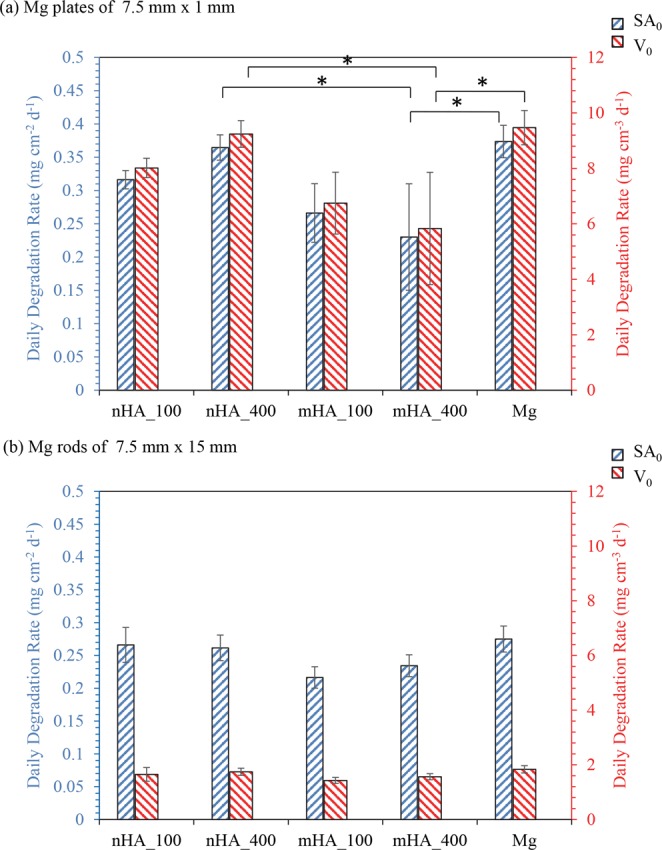
Average daily Mg^2+^ release rates per unit initial surface area (SA_0_, blue) and per unit initial volume (V_0_, red) after 6 weeks of immersion in rSBF. (**a**) Average daily Mg^2+^ release rates of nHA_100, nHA_400, mHA_100, mHA_400 coated and non-coated Mg plates with an initial dimension of 7.5 mm in diameter and 1 mm in height. (**b**) Average daily Mg^2+^ release rates of nHA_100, nHA_400, mHA_100, mHA_400 coated and non-coated Mg rods with an initial dimension of 7.5 mm in diameter and 15 mm in height. Data are mean ± standard deviation (N = 3); *p < 0.05.

**Figure 18:**
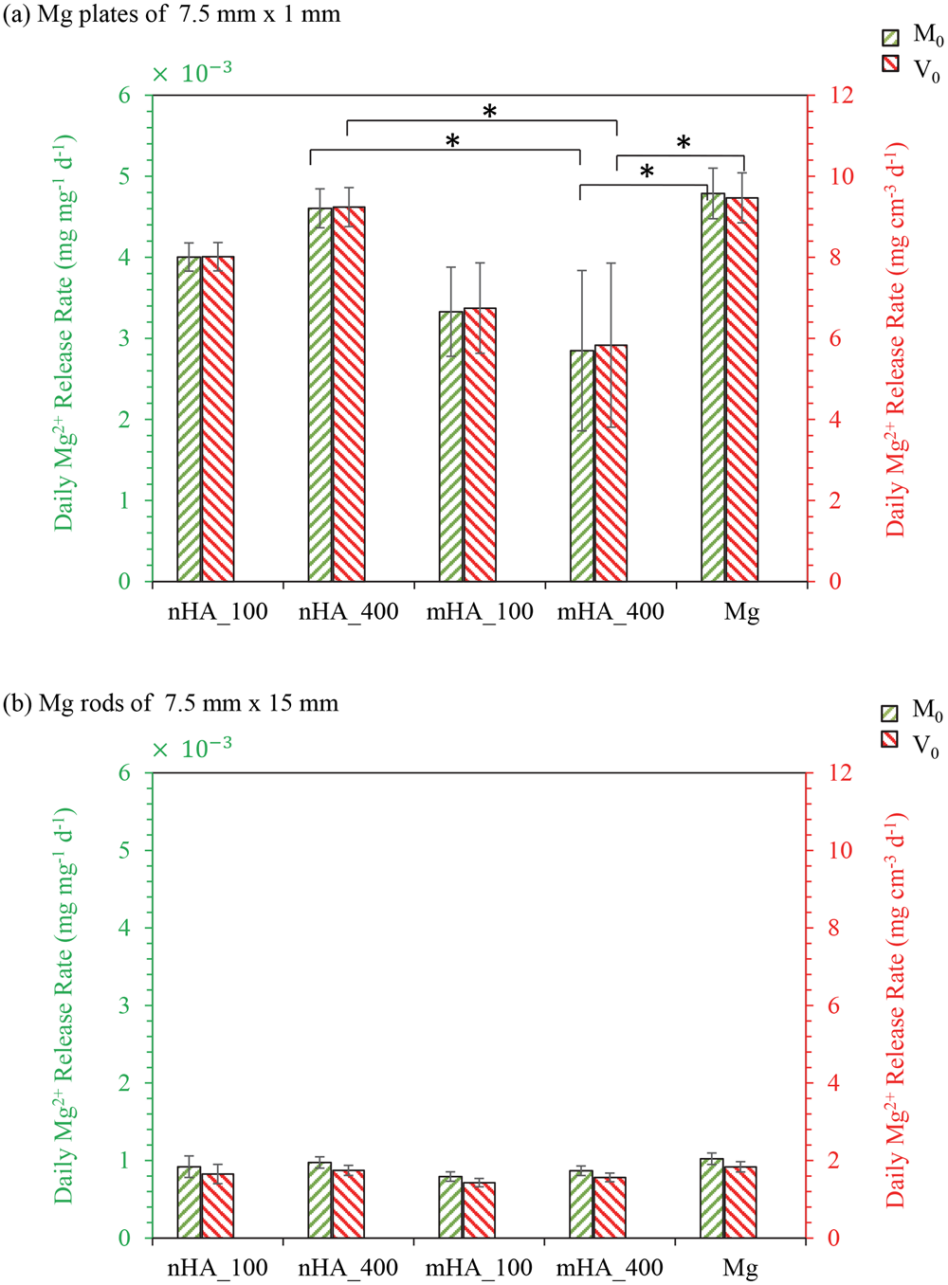
Average daily Mg^2+^ release rates per unit initial mass (M_0_, green) and per unit initial volume (V_0_, red) after 6 weeks of immersion in rSBF. (**a**) Average daily Mg^2+^ release rates of nHA_100, nHA_400, mHA_100, mHA_400 coated and non-coated Mg plates with an initial dimension of 7.5 mm in diameter and 1 mm in height. (**b**) Average daily Mg^2+^ release rates of nHA_100, nHA_400, mHA_100, mHA_400 coated and non-coated Mg rods with an initial dimension of 7.5 mm in diameter and 15 mm in height. Data are mean ± standard deviation (N = 3); *p < 0.05.

The *in vitro* average daily Mg^2+^ release rates of the nHA and mHA coated as well as non-coated Mg plates or rods were calculated following the equation 1 described above and plotted in Figs 17 and 18. The blue rectangular bars in Fig. 17 represent the average daily Mg^2+^ release rates normalized by the surface area of the respective samples; the red rectangular bars in Figs 17 and 18 represent the average daily Mg^2+^ release rates normalized by the volume of the respective samples; and the green rectangular bars in Fig. 18 represent the average daily Mg^2+^ release rates normalized by the mass of the respective samples. As shown in Fig. 17(a), the average daily Mg^2+^ release rates of the nHA and mHA coated Mg plates, whether normalized by surface area or volume, were slower than that of the non-coated Mg plates. ANOVA showed statistically significant differences in the Mg^2+^ release rates of the Mg plates normalized by surface area [F = 5.787, p = 0.044]. Post-hoc pairwise comparisons showed that the *in vitro* average daily Mg^2+^ release rate of the mHA_400 coated Mg plate (0.23 ± 0.08 mg cm^2^ d^−1^), as normalized by surface area, was significantly lower than that of the Mg (0.37 ± 0.02 mg cm^2^ d^−1^) and nHA_400 (0.36 ± 0.02 mg cm^2^ d^−1^). Similarly, ANOVA showed statistically significant differences in the Mg^2+^ release rates of the plates normalized by volume [F = 5.787, p = 0.044]. Post-hoc pairwise comparisons showed that the *in vitro* average daily Mg^2+^ release rate of the mHA_400 coated Mg plate (5.83 ± 2.02 mg cm^3^ d^−1^), as normalized by volume, was significantly lower than that of the Mg (9.47 ± 0.62 mg cm^3^ d^−1^) and nHA_400 (9.24 ± 0.48 mg cm^3^ d^−1^).

Figure 17(b) shows the average daily Mg^2+^ release rates of the nHA and mHA coated Mg rods normalized either by surface area or volume, which were all slower than that of the non-coated Mg rods in average. ANOVA showed no statistically significant differences found in the Mg^2+^ release rates of the Mg rods normalized either by surface area or volume [F = 3.70, p = 0.093]. Nevertheless, mHA_100 coated Mg rods showed the lowest average daily Mg^2+^ release rate normalized either by surface area (0.22 ± 0.02 mg cm^2^ d^−1^) or volume (1.43 ± 0.11 mg cm^3^ d^−1^) among all the samples. Even though not statistically significant, mHA_400 coated Mg rods still showed slower average daily Mg^2+^ release rate than nHA_100 and nHA_400 coated Mg rods, when normalized either by surface area (0.23 ± 0.02 mg cm^2^ d^−1^) or volume (1.56 ± 0.11 mg cm^3^ d^−1^).

Figure 18(a) shows the average daily Mg^2+^ release rates of the nHA and mHA coated Mg plates normalized by either mass (green bars) or volume (red bars). The trends for green or red bars were similar because the density of the samples was regarded as the same, since Mg has a theoretical density of 1.74 g/cm^3^ and the coatings have little effect on the sample density. Post-hoc pairwise comparisons showed that the *in vitro* average daily Mg^2+^ release rate of the mHA_400 coated Mg plate, whether normalized by mass or volume, was significantly lower than that of the Mg and nHA_400. In Fig. 18(a), when comparing the two TPA pressure conditions of 100 versus 400, the nHA_100 and mHA_400 coated Mg plates showed generally lower average daily Mg^2+^ release rates than their counterparts of nHA_400 and mHA_100 coated Mg plates, but not statistically significant. Figure 18(b) shows the average daily Mg^2+^ release rates of the nHA and mHA coated Mg rods normalized by either mass (green bars) or volume (red bars). ANOVA showed no statistically significant differences among the Mg^2+^ release rates of the Mg rods normalized by either mass or volume. Even though not statistically significant, mHA_400 coated Mg rods still showed lower average daily Mg^2+^ release rate, as normalized by mass, than nHA_100 and nHA_400 coated Mg rods.

### Mechanical properties of the nHA and mHA coated Mg rods before and after Immersion

**Figure 19:**
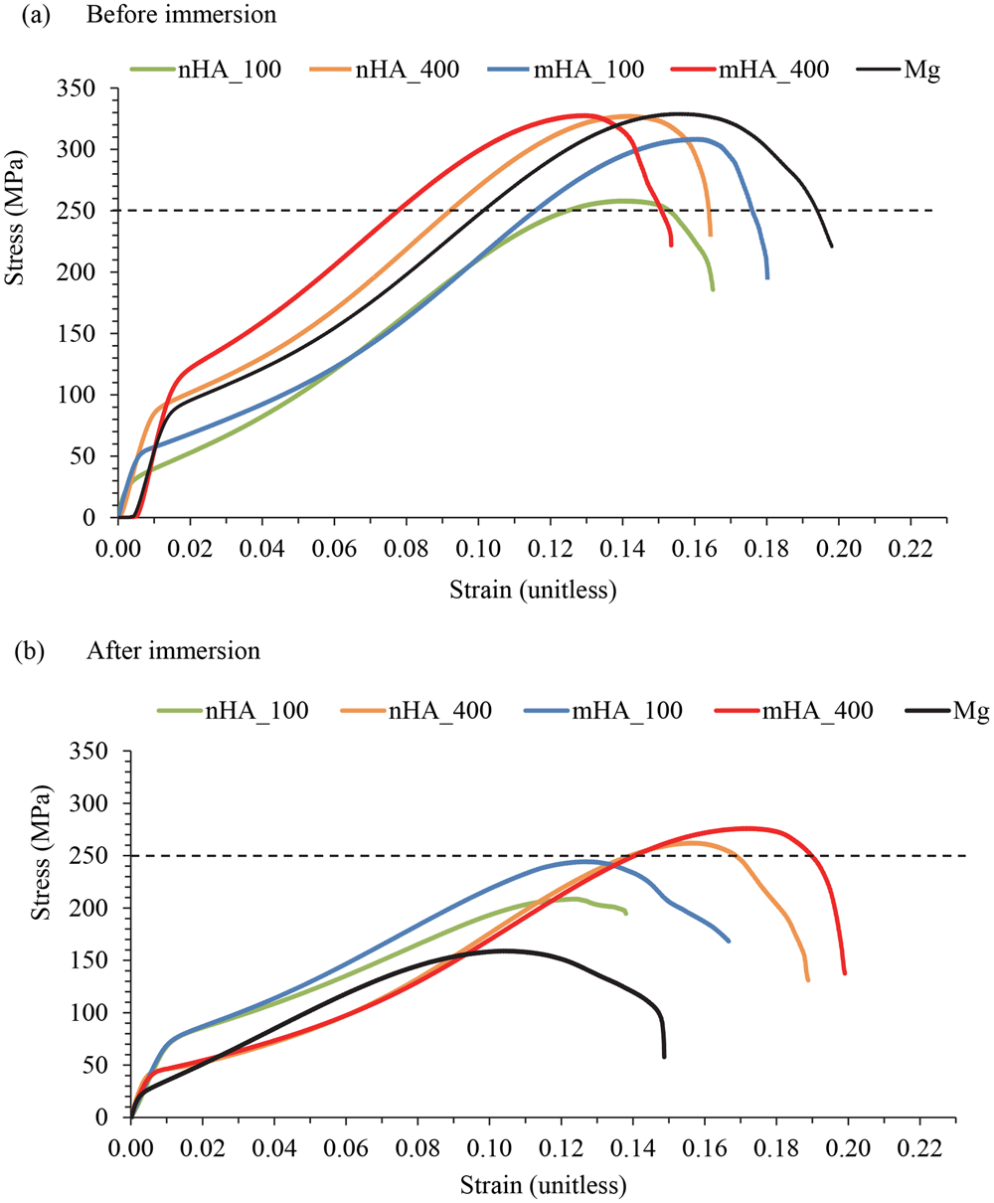
Representative stress-strain curves of nHA_100, nHA_400, mHA_100, and mHA_400 coated Mg rods, and non-coated Mg rod as a control. Mg rod samples had an initial dimension of 7.5 mm in diameter and 15 mm in height. (**a**) Stress-strain curves of all Mg-based rod samples before immersion. (**b**) Stress-strain curves of all Mg-based rod samples after immersed in rSBF for 6 weeks.

The representative stress-strain curves of 7.5 mm × 15 mm Mg rods, including nHA_100, nHA_400, mHA_100, and mHA_400 coated and non-coated Mg rods are summarized in Fig. 19. The calculated mechanical properties of respective Mg rods are summarized in Table 1. Before immersion, Mg rods had an ultimate compressive strength of 314 ± 14.0 MPa, where the nHA_400 coated Mg rods showed the highest average strength among all (319 ± 7.5 MPa). After immersion, the ultimate compressive strength of all Mg-based samples showed a significant decrease when compared with the respective counterparts before immersion, where Mg control showed the lowest compressive strength (208 ± 39.0 MPa) among all after immersion. The nHA_400 and mHA_400 showed a higher compressive strength than their counterparts of nHA_100 and mHA_100 respectively, indicating the high TPA pressure condition of 400 provided a better coating that protected Mg substrates from rapid degradation more effectively. After 6 weeks of immersion, nHA and mHA coated Mg showed less decrease in ultimate compressive strength. Specifically, the reduction in compressive strength was 9.8% for nHA_100, 13.2% for nHA_400, 13.7% for mHA_100, and 11.0% for mHA_400, when compared with the significant decrease in ultimate compressive strength of non-coated Mg (33.8%). The ultimate compressive load of Mg rods was around 14.0 kN for all the samples except nHA_100, which was 11.5 ± 0.1 kN before immersion. A significant decrease in maximum load was found for all the Mg-based samples after immersion of 6 weeks. Mg controls had the lowest ultimate compressive load of 9.0 ± 2.0 kN, and the nHA_400 and mHA_400 maintained a higher ultimate compressive load than all the other samples. Young’s modulus of the Mg-based samples had a similar trend to the ultimate stress and maximum load, as shown in Table 1. Specifically, all coated and non-coated Mg rods showed a significant decrease in Young’s modulus after 6 weeks of immersion; and, non-coated Mg rod control showed the lowest Young’s modulus (2.9 ± 1.4 GPa) after immersion.

**Table 1:** Mechanical properties of nHA_100, nHA_400, mHA_100, mHA_400 coated and non-coated Mg rods with an initial geometry of 7.5 mm × 15 mm before and after 6 weeks of immersion in rSBF.

Samples		nHA_100	nHA_400	mHA_100	mHA_400	Mg
Ultimate compressive strength (MPa)	Before immersion	259 ± 1.0	319 ± 7.5	306 ± 1.0	309 ± 18.5	314 ± 14.0
	After immersion	234 ± 18.5	277 ± 14.5	264 ± 20.0	275 ± 1.5	208 ± 39.0
Ultimate compressive load (kN)	Before immersion	11.5 ± 0.1	14.1 ± 0.4	13.6 ± 0.1	13.6 ± 0.8	13.6 ± 0.9
	After immersion	10.5 ± 1.0	12.3 ± 0.7	11.7 ± 0.9	12.3 ± 0.1	9.0 ± 2.0
Young’s modulus (GPa)	Before immersion	9.8 ± 2.3	9.1 ± 0.4	8.4 ± 0.8	9.3 ± 1.5	9.9 ± 1.1
	After immersion	4.4 ± 0.7	4.4 ± 2.0	5.3 ± 1.2	6.1 ± 0.6	2.9 ± 1.4

### Cytocompatibility of nHA and mHA coated Mg plates with BMSCs in direct culture *in vitro*

**Figure 20:**
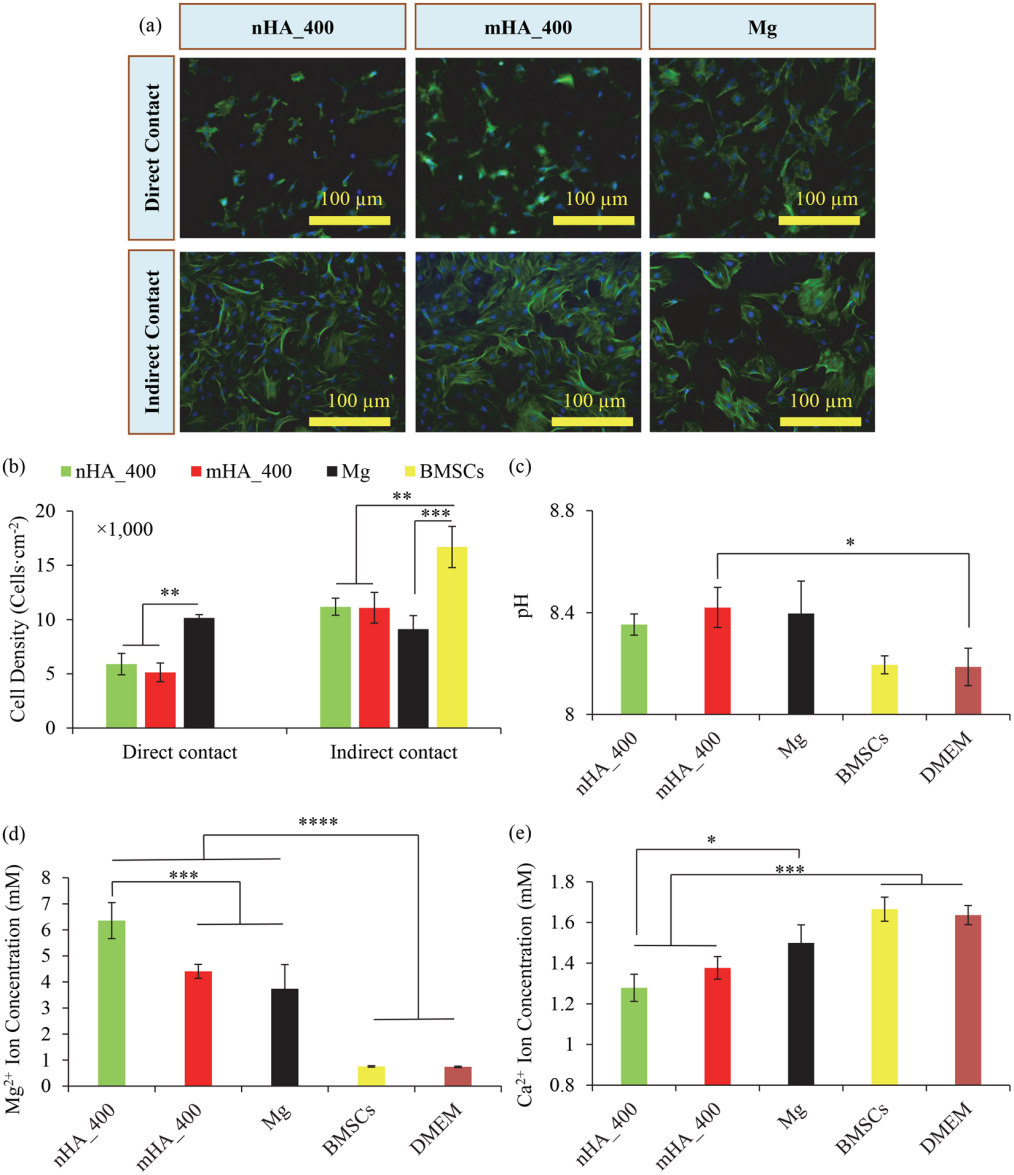
*In vitro* direct cutlure of nHA_400, mHA_400 and Mg plates with BMSCs for 24 hours and the resulted BMSC morphology, cell adhesion density, and post-culure media analyses. Mg plate samples had an initial dimension of 7.5 mm in diameter and 1 mm in height. (**a**) Flourensence images of BMSCs that were under direct contact or indirect contract conditions with nHA_400, mHA_400 and Mg plates after 24 hours of direct culture. (**b**) BMSC adhesion densities after 24 hours of culture were quantified based on the fluoresence images using ImageJ. (**c**) The pH of media after cultured with the respective Mg plate samples. (**d**) Mg^2+^ ion concentrations in the media after 24 hours of BMSC culture. (**e**) Ca^2+^ ion concentrations in the media after 24 hours of BMSC cutlure. Data are mean ± standard deviation (N = 3); *p < 0.05, ***p < 0.001, ****p < 0.0001.

The adhesion and morphology of BMSCs after the 24-hour culture on and around nHA_400, mHA_400 and Mg plates are shown in the representative fluorescence images under direct contact and indirect contact conditions in Fig. 20a. The BMSCs on nHA_400 and mHA_400 showed different morphologies when compared with the BMSCs on the non-coated Mg plates, most likely because the surface chemistry, microstructure, and topography of the nHA or mHA coated Mg samples were different from non-coated Mg. Quantitatively, in Fig. 20b, the samples of nHA_400 and mHA_400 showed statistically lower cell adhesion densities on the surface than that of the Mg plate under direct contact conditions. Under indirect contact conditions, no statistical difference was found among the groups of nHA_400, mHA_400, and Mg plates, but in average the groups of nHA_400 and mHA_400 showed greater cell densities than that of the non-coated Mg plate. The Mg-based samples showed significantly lower BMSC adhesion densities than that of the BMSCs control under indirect contact conditions of direct culture.

The pH of the media after cultured with mHA_400 was statistically higher than that of DMEM control, but the difference was small, as shown in Fig. 20c. The other groups showed no statistical difference in media pH. In Fig. 20d, the nHA_400, mHA_400 and Mg showed statistically higher Mg^2+^ ion concentrations in the post-culture media than the control groups of BMSCs and DMEM only. The nHA_400 showed a higher release of Mg^2+^ ions than mHA_400 and Mg, indicating a higher degradation rate in the BMSC/DMEM direct culture in 24 hours. In Fig. 20e, reductions in Ca^2+^ ion concentrations were observed in the post-culture media with all Mg-based samples, indicating mineral deposition on these samples. The nHA_400 showed the lowest average Ca^2+^ ion concentration after culture, which was statistically lower than that of non-coated Mg plate. Both nHA coated and mHA coated Mg plates showed statistically lower Ca^2+^ ion concentrations than the control groups of BMSCs only and DMEM only, after 24 hours of culture. The correlations between Mg^2+^ and Ca^2+^ ion concentrations in the BMSC culture were similar to the results observed in the immersion degradation study in rSBF. That is, the higher Mg^2+^ ion releases corresponded to the lower Ca^2+^ ion concentrations in the post-culture media, likely because more Ca^2+^ ions from the media precipitated onto the sample surface to form the degradation layer.

## Discussion

### HA coatings in controlling the degradation rates of Mg-based implants at the bone interface

Generally, depending on implant type, anatomical location of bone and healing capacity of different individuals, 4 to 12 weeks are required for bone repair^5^. Therefore, it is preferable for Mg-based biodegradable implants to retain their mechanical integrity *in vivo* over a time scale of 12 to 18 weeks for bone tissue regeneration^10^. The main roles of HA coating are to improve the corrosion resistance, reduce the degradation rate of Mg substrates, and prolong the mechanical strength of Mg during *in vivo* degradation, in addition to accelerating bone healing. The previous study has shown that nHA coatings on Mg substrates improved the corrosion resistance and reduced degradation rates^10^. In this study, two TPA process conditions were used to deposit nHA and mHA coatings on Mg plates and rods that represent the typical dimensions of bone fixation devices for CMF and orthopedic applications.

This study contributed to the field of Mg-based biodegradable implants and devices by establishing highly reproducible coating processes for controlling the degradation rates of Mg and Mg alloys for a wide range of applications. Surface characterization of the HA coatings (Figs 1 and 2) suggested that the high pressure TPA condition (_400) provided a more homogeneous distribution of HA particles on Mg substrates with less aggregation of particles and a higher packing density when compared with the low pressure TPA condition (_100). Among the nHA and mHA coatings, nHA_400 had the greatest coating thickness (Fig. 5). The thicker coatings may be able to decrease Mg degradation more than thinner coatings due to barrier effect, but the thicker coating may induce more defects such as cracks^28^ that allow aggressive ions to penetrate the coating and attack the underlying Mg substrate. Collectively, the mHA_100 and mHA_400 coated plates and rods showed lower degradation rates than the nHA coated counterparts (Fig. 17).

The degradation behaviors of the coated and non-coated Mg samples were further studied via quantitative analysis of the post-cultured media (rSBF) at each time point. During immersion in rSBF, the degradation of Mg led to the increases of Mg^2+^ ion concentration and pH in rSBF; The higher Mg^2+^ ion concentration in rSBF cultured with nHA_100 and nHA_400 coated Mg indicated the higher diffusion of media to Mg substrates through the nHA coatings when compared with mHA coatings. The cracks in nHA_400 and nHA_100 coatings could also contribute to the higher degradation rates of nHA_400 and nHA_100 coated Mg plates than the mHA coated plates, because aggressive ions from media could penetrate through cracks more easily to attack the Mg substrates. Moreover, OH^−^ ions released from Mg degradation could react with Ca^2+^ ions in rSBF to form precipitates on the sample surface, as confirmed by the decrease of Ca^2+^ ion concentration in the culture media (Figs 15c and 16c). Generally, the non-coated Mg plates and rods showed higher pH and Mg^2+^ ion concentration in the rSBF at different time points (Figs 15a,b and 16a,b), which indicated that nHA and mHA coatings improved corrosion resistance when compared with non-coated Mg. Among the nHA and mHA coated Mg plates, mHA_400 coated Mg plates showed the lowest degradation rate among all the plates (Fig. 17a), likely because of its crack-free and relatively thicker coating on Mg substrate.

### HA coatings in retaining the mechanical properties of Mg-based implants for a longer period

The bone fixation devices such as plates and screws typically experience tension and compression stresses when used, and such stresses could lead to undesirable mechanical failure, which has been reported for orthopedic implants made of stainless steel and titanium alloys^29^. When coupled with stresses, localized corrosion such as pitting could adversely affect the mechanical properties of Mg-based implants^29^. Increased corrosion resistance will reduce the initial degradation rate of Mg-based implants, which is critical for maintaining their mechanical properties during the period of bone healing. The ultimate compressive strengths of nHA and mHA coated Mg and non-coated Mg rods before immersion in rSBF (260–320 MPa in average) were found to be higher than the values reported in literature; for example, the ultimate compressive strength of the HA coated Mg prepared by pulse electrodeposition process was reported to be 175 MPa and non-coated Mg was 162 MPa^29^. In this study, the nHA and mHA coated Mg rods showed improvement in ultimate compressive strength when compared with non-coated Mg control after 6 weeks of immersion in rSBF (Table 1), because the nHA and mHA coatings enhanced the resistance to localized corrosion and impeded the stress-assisted corrosion process. Among all the coated and non-coated Mg rods, the nHA_400 and mHA_400 coated rods showed the highest average compressive strength (277 ± 14.5 MPa and 275 ± 1.5 MPa, respectively) after 6 weeks of immersion; moreover, the mHA_400 coated Mg showed lower degradation rate and crack-free surface microstructure. Collectively, mHA_400 coated Mg could support fractured bones and retain structural stability for a longer period than non-coated Mg, thus providing better mechanical and degradation properties for bone fixation devices.

### Formation and function of crystalline degradation products on Mg

Formation of MgCO_3_∙3H_2_O and CaCO_3_ on the surface after immersion degradation was confirmed in this study. Previous studies^30,31^ have shown MgCO_3_ and CaCO_3_ phases in the degradation products after immersion in physiological solutions such as Dulbecco’s modified eagle medium, Hank’s solution, and simulated body fluid. It was revealed that MgCO_3_ and CaCO_3_ phases could form a dense and protective layer to reduce degradation and completely cover the surface of Mg alloy after immersion, while Mg(OH)_2_ as degradation products can hardly form effective protection from degradation^31^. Uan *et al*. reported the improvement in corrosion resistance of the Mg alloy coated with CaCO_3_^32^. It was also reported that CaCO_3_ coating on Mg alloy enhanced the initial cell adhesion on the surface when compared with the non-coated Mg alloy^33^. In this study, the equilibrium solubility of CO_2_ in water was 170 mM under the standard incubation condition of 5% CO_2_/95% air, making it possible for the reaction between Mg^2+^/Ca^2+^ and H_2_CO_3_^34^. The carbon dioxide (CO_2_) dissolves in water and forms H_2_CO_3_ to provide the buffering effect for bicarbonate-containing media. The release of OH^−^ from corrosion reaction will shift the equilibrium of bicarbonate/carbon dioxide buffering system to the production of ${{\rm{CO}}}_{3}^{2-}$, which further induced MgCO_3_∙3H_2_O and CaCO_3_ formation^34^. Guan *et al*. showed that HA, Mg(OH)_2_, and CaCO_3_ phases precipitated as degradation products during *in vitro* immersion of HA coated Mg^35^, in agreement with the results of this study. In this study, after 6 weeks of immersion, the HA phase was detected on nHA and mHA coated Mg plates (Fig. 11), but not on nHA and mHA coated Mg rods (Fig. 12); in contrast, carbonates of MgCO_3_∙3H_2_O and CaCO_3_ were detected on all samples of Mg-based plates and rods. SEM images in Fig. 7 showed that the original HA coatings on Mg-based plates were still visible after immersion degradation, but they were barely visible on Mg-based rods after immersion, as shown in Fig. 9. The most likely reason is that more carbonate degradation products deposited on Mg-based rods than that of plates and thus covered the surfaces of Mg-based rods. Moreover, in the same volume of immersion media (3 mL), the rod samples had a greater mass and volume than the plate samples, i.e., a greater ratio of sample volume to media volume, and resulted in greater pH and Mg^2+^ ion concentrations around the rods than the plates, which exceeded the solubility of MgCO_3_ in aqueous media. Overall, the precipitation of dense MgCO_3_∙3H_2_O and CaCO_3_ layer on the surfaces of nHA and mHA coated Mg rods improved corrosion resistance; in contrast, the loose degradation layer of mixed phases of MgO, MgCO_3_∙3H_2_O, CaCO_3_ and HA on nHA and mHA coated Mg plates was less effective in moderating the degradation.

### The effects of sample geometry, and dimension, volume, and mass on degradation behaviors of Mg-based implants

The results of this study first demonstrated the effects of different sample geometry (e.g., plates versus rods), dimension, volume, and mass on the degradation behaviors of Mg *in vitro*. Specifically, Mg-based plates and rods showed significant differences in morphology and composition of degradation layers, mass change, pH change, and ion (Mg^2+^, Ca^2+^) concentrations in the immersion media rSBF. The nHA and mHA coated Mg plates showed loose degradation products that partially covered the surface, with typical degradation morphology of non-coated Mg, i.e., corrosion cracks. The nHA and mHA coated Mg rods, however, showed the crystalline degradation products that fully covered the surface. As described above, the dissolved CO_2_ in rSBF built a bicarbonate/carbon dioxide buffering system under the standard cell culture conditions. Although Mg rods had a lower degradation rate than that of the Mg plates, the amount of OH^−^ and Mg^2+^ in the media cultured with Mg rods was generally higher than that in the media cultured with the Mg plates, because the rods had a greater volume and mass than the plate samples and were immersed in the same volume of rSBF (3 mL). An implant used at the same anatomical location inside the human body is typically exposed to a similar amount of body fluid with similar composition *in vivo*. Therefore, higher pH and Mg^2+^ ion concentrations around Mg rods promoted the formation of protective MgCO_3_ crystals on the top surface. The growth process of MgCO_3_∙3H_2_O whiskers over time at room temperature was investigated by Wang *et al*.^36^. The high pH of the Mg-based rods might have induced more precipitation of MgCO_3_∙3H_2_O crystals on the surface and offered more nucleation sites for MgCO_3_∙3H_2_O precursor whiskers^36^; as a result, larger and more thermodynamically stable crystals formed during degradation. The MgCO_3_∙3H_2_O precursor whiskers grew, extended and aggregated together to decrease the surface energy. In this study, layered morphology of crystals on Mg-based rods in Fig. 9 demonstrated aggregation of whiskers, in agreement with Wang *et al*.^36^. In contrast, the lower pH of Mg-based plates during the initial 24 hours induced less precipitation of MgCO_3_∙3H_2_O crystals on the surface, and decreased the number of nucleation rates of MgCO_3_∙3H_2_O precursor whiskers. Therefore, MgCO_3_∙3H_2_O whiskers could not form stable crystal aggregates in the degradation layer of Mg-based plates after 6 weeks of immersion; instead, loose degradation products on the surface facilitated the degradation process of Mg plates. In terms of mass change, Mg-based plates showed mass loss, while Mg-based rods showed mass gain during the 6 weeks of immersion in rSBF, whether the samples were coated or not. This confirmed more deposition of degradation products on the Mg-based rod samples than on the Mg-based plate samples. Generally, when immersed in the same volume of rSBF under the same environmental conditions, the lower volume and mass of Mg plates induced less pH increase, less release of Mg^2+^ ions, and less deposition of Ca^2+^ ions from rSBF than the Mg rods that had a greater volume and mass. In other words, Mg^2+^ ions around Mg rods could reach saturation due to solubility limit, which would increase the precipitation of degradation products and decrease the degradation rates.

Plates and rods in this study were used to mimic bone fixation plates and screws; and the greater surface-to-volume ratio of the Mg plates (25.3 cm^−1^) than Mg rods (6.7 cm^−1^) was likely the main reason for faster daily Mg^2+^ release rates of Mg-based plates than rods (Fig. 17). The faster daily Mg^2+^ release rates indicated a faster daily degradation rate of Mg-based plates. Greater surface area offers more sites for degradation reactions to occur. For a plate with a diameter of 7.5 mm and a thickness of 1 mm and a rod with a diameter of 1.94 mm and a length of 15 mm, they would have the same volume of 44 mm^3^; however, the plate has a higher surface area of 112 mm^2^ than that of the rod of 97 mm^2^. This means that the plate still has a higher surface area to volume ratio for possibly faster degradation than the rod, even when their volume and mass are the same. In a previous *in vivo* study, Mg plates implanted in a rabbit ulna fracture site showed greater corrosion rate than Mg screws, the difference in their local environment *in vivo* was considered as the main reason for the difference in the degradation of plates versus screws^37^. Specifically, the screws were implanted mostly within bone while the plates were initially covered by muscles; the higher water content and blood flow in muscles than compact bone could have accelerated the plate corrosion^37^. In this study, the immersion degradation of Mg-based plates and rods was performed in the same solution (that is, rSBF) with the same volume and under the same environment (that is, the standard cell culture condition); however, the degradation rates of Mg-based plates were still faster than the rods after being normalized by their respective volume or mass (Fig. 18). This suggested that Mg-based implants of different design and geometry would degrade differently even when they were implanted at the same or similar anatomical locations. Realistically, fixation plates, rods, and screws in clinical applications usually do not have the same mass and volume. Therefore, the geometries and dimensions of Mg-based implants and devices, especially surface-area-to-volume ratio, should be taken into special considerations in their design and processing, to ensure the device sets could fulfill clinical functions for a specific period of time and provide structural stability during tissue healing.

### BMSC behaviors in direct contact and indirect contact with nHA and mHA coated Mg in direct culture *in vitro*

When compared with the non-coated Mg group, the BMSCs on the nHA_400 and mHA_400 coated Mg groups showed statistically lower cell adhesion densities with less-spread cell morphologies under direct contact conditions; however, under the indirect contact conditions, the average cell adhesion densities around the nHA_400 and mHA_400 coated Mg were higher with well-spread BMSC morphologies. These results are in agreement with the previous studies that showed similar cell behaviors on HA-coated Mg and non-coated coated Mg controls^10,20^. The different cell behaviors under direct versus indirect contact conditions have also been reported in the previous *in vitro* studies of Mg and Mg alloys in direct cutlure^3,38–40^.

In the direct culture, the direct contact conditions could reveal cell responses to surface chemistry, microstructure, topography, and dynamic degradation directly at the cell-material interface. In comparison with non-coated Mg plates, the nHA or mHA coatings changed surface chemistry (Fig. 3d) and microstructure (Figs 2 and 3), which could directly affect BMSCs attached on the surface. When the non-coated Mg plates were cultured in the BMSC/DMEM culture system, magnesium oxide (MgO) and magnesium hydroxide (Mg(OH)_2_) would form on the surface^12^, and nanostructured MgO in a small dosage could enhance BMSC adhesion density in the DMEM culture as reported by Wetteland *et al*.^41^. Therefore, the oxide-containing degradation layer on Mg plates could possibly contribute to the observed higher cell adhesion density on non-coated Mg plates than on nHA or mHA coated Mg plates. In contrast, under indirect contact conditions in the direct culture, the solubilized ions released from the degrading samples to media might play more important roles in cell adhesion and morphology around the samples. The nHA and mHA coated Mg plates showed cell adhesion densities over 10,000 cell/cm^2^, indicating that the BMSCs proliferated during the 24-hour direct culture. It has been reported that neither culture media supplemented with up to 27.6 mM Mg^2+^ ions nor media transiently adjusted up to alkaline pH 9 induced any detectable adverse effects on BMSC responses^38^. In this study, the average Mg^2+^ ion concentrations in the BMSC/DMEM culture reached 6.36 mM, 4.41 mM, and 3.74 mM after 24 hours of culture with nHA coated, mHA coated, and non-coated Mg, respectively. The average pH was below 9 and the average Mg^2+^ ion concentrations were below 27.6 mM for all Mg-based groups in this study, indicating the degradation-induced dynamic concentration gradient on the surface and around the sample in the direct culture was also important for BMSC responses. The direct culture method could capture the effects of dynamic concentration gradient on cell behaviors, but the method of doping media with degradation products could not. The continuously elevated pH and Mg^2+^ ion concentrations, as well as reduced Ca^2+^ ion concentrations, in the direct culture with Mg-based samples might have contributed to the lower cell adhesion densities around the coated and non-coated Mg plates than the BMSCs control. This *in vitro* study with BMSCs was carried out for only 24 hours to capture cell responses in the initial period of *in vivo* implantation. Further *in vitro* and *in vivo* studies in longer periods are still needed to elucidate the effects of nHA and mHA coatings on cell functions and tissue regeneration.

## Conclusions

The nHA and mHA coatings were deposited on Mg plates and rods using the patented transonic particle acceleration (TPA) deposition process under two different conditions and their degradation and mechanical properties were investigated *in vitro* in rSBF for 6 weeks. The nHA and mHA coatings reduced the degradation rates of Mg plates and rods; and the coated Mg rods retained better mechanical properties than non-coated Mg rod after 6 weeks of immersion in rSBF. Generally, mHA_400 coated Mg showed a good combination of consistent surface microstructure, coating thickness, and slower degradation rates in rSBF over 6 weeks of immersion. Moreover, mHA_400 coated Mg rods retained 89% of compressive strength after 6-week immersion (275 ± 1.5 MPa), which still meets the mechanical requirement for load-bearing implants. The geometry, dimension, surface area, volume, and mass of Mg-based implants affected the *in vitro* degradation properties, including degradation rates and modes, when the environmental conditions were set to be the same. The nHA and mHA coated Mg rods, which had a lower surface-to-volume ratio (6.7 cm^−1^), showed slower degradation than the nHA and mHA coated Mg plates that has a greater surface-to-volume ratio (25.3 cm^−1^). After 6 weeks of immersion in rSBF, dense crystalline degradation layers covered the surface of the Mg rods; however, loose degradation layers formed on the surface of the Mg plates. Different geometry, dimension, surface area, volume, and mass of Mg rods versus plates resulted in significant differences in degradation modes and rates when they had the same HA coatings and were immersed in the same volume of immersion media under the same environment, and thus should be taken into account in Mg-based biodegradable device design and processing. In the 24-hour direct culture with BMSCs *in vitro*, the nHA and mHA coated Mg reduced BMSC adhesion densities directly on the surface under direct contact, but increased the average BMSC adhesion densities under indirect contact, when compared with non-coated Mg. The surface chemistry, microstructure, and morphology of nHA and mHA coatings on Mg could play key roles in BMSC adhesion and morphology under direct contact, but BMSC adhesion and morphology under indirect contact were mostly affected by the releases of soluble degradation products from sample degradation and the dynamic concentration gradient around the Mg-based samples. Collectively, Mg-based biodegradable metals coupled with nHA and mHA coatings are promising for load-bearing implant applications, and should be further studied in long-term *in vitro* and *in vivo* models toward potential clinical translation.

## Supplementary information

Dataset for SREP-18-29489A

## Data Availability

All data generated or analyzed during this study are included in this published article and its Supplementary Information Files.
