# Supplementary figures and images for: Nano-to-Submicron Hydroxyapatite Coatings for Magnesium-based Bioresorbable Implants – Deposition, Characterization, Degradation, Mechanical Properties, and Cytocompatibility (part 2 of 2)
Source: Sci Rep. 2019 Jan 28;9:810. doi: 10.1038/s41598-018-37123-3 (PMC6349930; doi:10.1038/s41598-018-37123-3)

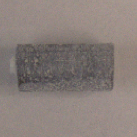

Supplement: Supplementary file 1 — Dataset for SREP-18-29489A [file 41598_2018_37123_MOESM1_ESM.zip › SupplementalDataFiles/Figure 8/24 hours/nHA_100.png]

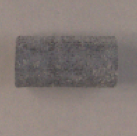

Supplement: Supplementary file 1 — Dataset for SREP-18-29489A [file 41598_2018_37123_MOESM1_ESM.zip › SupplementalDataFiles/Figure 8/24 hours/nHA_400.png]

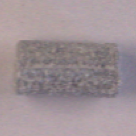

Supplement: Supplementary file 1 — Dataset for SREP-18-29489A [file 41598_2018_37123_MOESM1_ESM.zip › SupplementalDataFiles/Figure 8/4 wks/Mg.png]

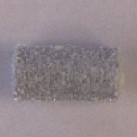

Supplement: Supplementary file 1 — Dataset for SREP-18-29489A [file 41598_2018_37123_MOESM1_ESM.zip › SupplementalDataFiles/Figure 8/4 wks/mHA_100.png]

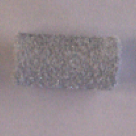

Supplement: Supplementary file 1 — Dataset for SREP-18-29489A [file 41598_2018_37123_MOESM1_ESM.zip › SupplementalDataFiles/Figure 8/4 wks/mHA_400.png]

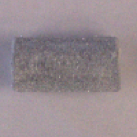

Supplement: Supplementary file 1 — Dataset for SREP-18-29489A [file 41598_2018_37123_MOESM1_ESM.zip › SupplementalDataFiles/Figure 8/4 wks/nHA_100.png]

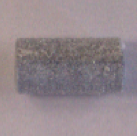

Supplement: Supplementary file 1 — Dataset for SREP-18-29489A [file 41598_2018_37123_MOESM1_ESM.zip › SupplementalDataFiles/Figure 8/4 wks/nHA_400.png]

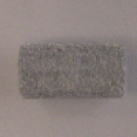

Supplement: Supplementary file 1 — Dataset for SREP-18-29489A [file 41598_2018_37123_MOESM1_ESM.zip › SupplementalDataFiles/Figure 8/48 hours/Mg.png]

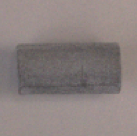

Supplement: Supplementary file 1 — Dataset for SREP-18-29489A [file 41598_2018_37123_MOESM1_ESM.zip › SupplementalDataFiles/Figure 8/48 hours/mHA_100.png]

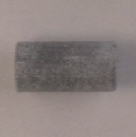

Supplement: Supplementary file 1 — Dataset for SREP-18-29489A [file 41598_2018_37123_MOESM1_ESM.zip › SupplementalDataFiles/Figure 8/48 hours/mHA_400.png]

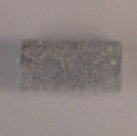

Supplement: Supplementary file 1 — Dataset for SREP-18-29489A [file 41598_2018_37123_MOESM1_ESM.zip › SupplementalDataFiles/Figure 8/48 hours/nHA_100.png]

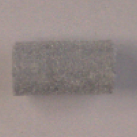

Supplement: Supplementary file 1 — Dataset for SREP-18-29489A [file 41598_2018_37123_MOESM1_ESM.zip › SupplementalDataFiles/Figure 8/48 hours/nHA_400.png]

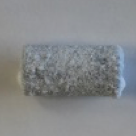

Supplement: Supplementary file 1 — Dataset for SREP-18-29489A [file 41598_2018_37123_MOESM1_ESM.zip › SupplementalDataFiles/Figure 8/6 wks/Mg.png]

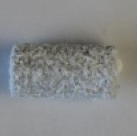

Supplement: Supplementary file 1 — Dataset for SREP-18-29489A [file 41598_2018_37123_MOESM1_ESM.zip › SupplementalDataFiles/Figure 8/6 wks/mHA_100.png]

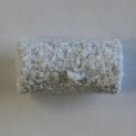

Supplement: Supplementary file 1 — Dataset for SREP-18-29489A [file 41598_2018_37123_MOESM1_ESM.zip › SupplementalDataFiles/Figure 8/6 wks/mHA_400.png]

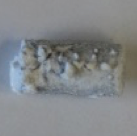

Supplement: Supplementary file 1 — Dataset for SREP-18-29489A [file 41598_2018_37123_MOESM1_ESM.zip › SupplementalDataFiles/Figure 8/6 wks/nHA_100.png]

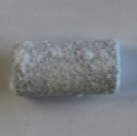

Supplement: Supplementary file 1 — Dataset for SREP-18-29489A [file 41598_2018_37123_MOESM1_ESM.zip › SupplementalDataFiles/Figure 8/6 wks/nHA_400.png]

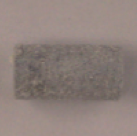

Supplement: Supplementary file 1 — Dataset for SREP-18-29489A [file 41598_2018_37123_MOESM1_ESM.zip › SupplementalDataFiles/Figure 8/72 hours/Mg.png]

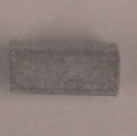

Supplement: Supplementary file 1 — Dataset for SREP-18-29489A [file 41598_2018_37123_MOESM1_ESM.zip › SupplementalDataFiles/Figure 8/72 hours/mHA_100.png]

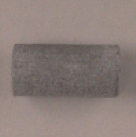

Supplement: Supplementary file 1 — Dataset for SREP-18-29489A [file 41598_2018_37123_MOESM1_ESM.zip › SupplementalDataFiles/Figure 8/72 hours/mHA_400.png]

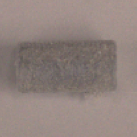

Supplement: Supplementary file 1 — Dataset for SREP-18-29489A [file 41598_2018_37123_MOESM1_ESM.zip › SupplementalDataFiles/Figure 8/72 hours/nHA_100.png]

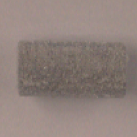

Supplement: Supplementary file 1 — Dataset for SREP-18-29489A [file 41598_2018_37123_MOESM1_ESM.zip › SupplementalDataFiles/Figure 8/72 hours/nHA_400.png]

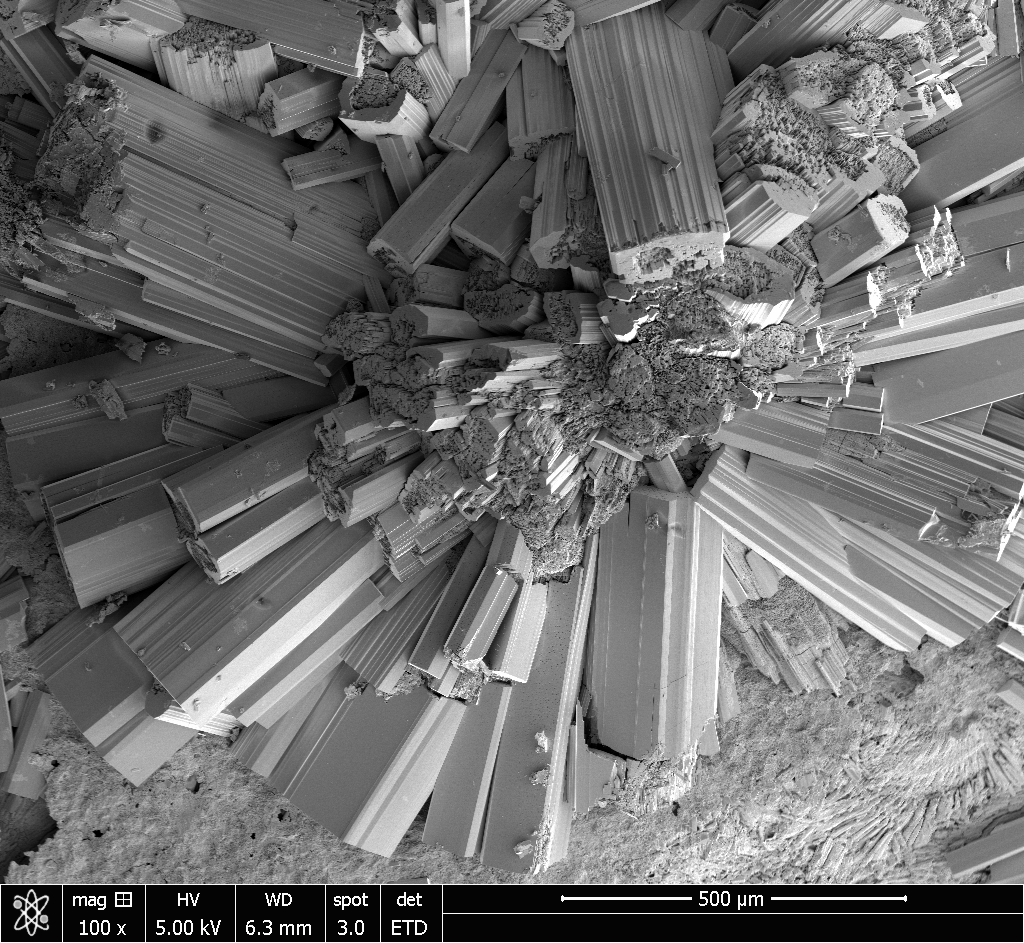

Supplement: Supplementary file 1 — Dataset for SREP-18-29489A [file 41598_2018_37123_MOESM1_ESM.zip › SupplementalDataFiles/Figure 9/Figure 9(a1).tif]

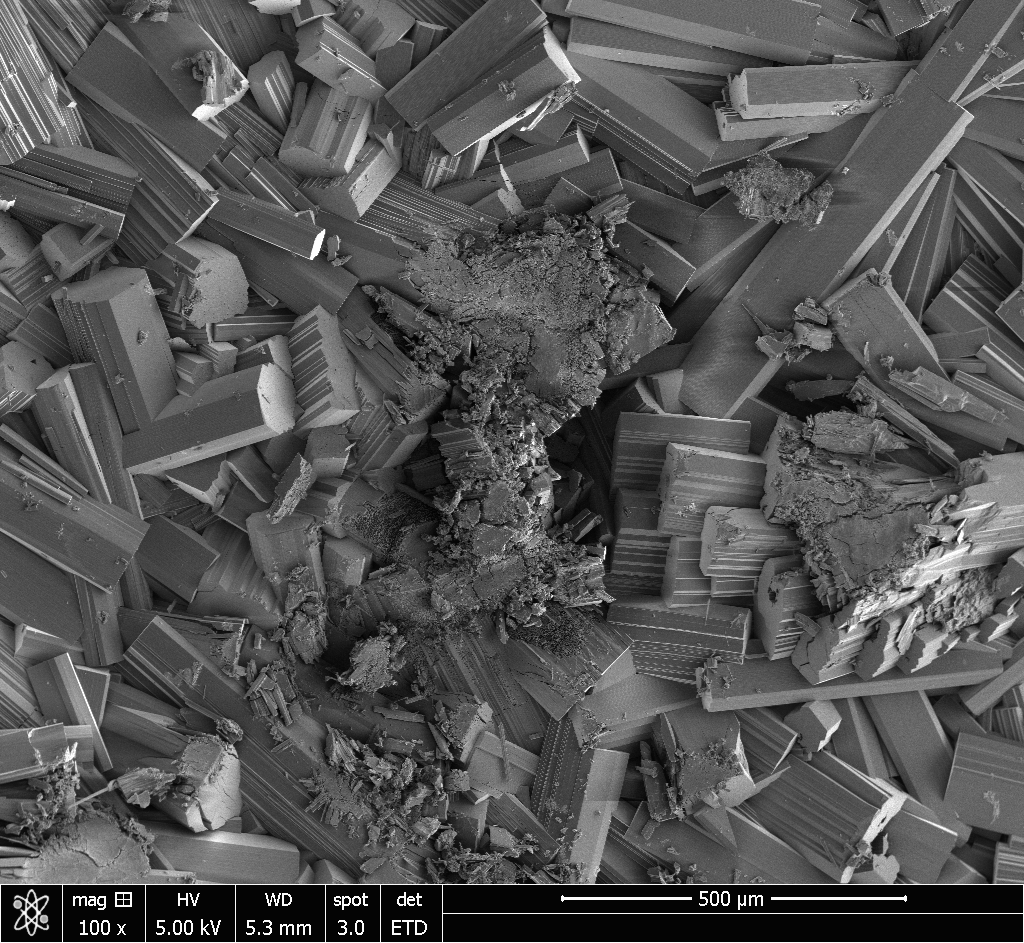

Supplement: Supplementary file 1 — Dataset for SREP-18-29489A [file 41598_2018_37123_MOESM1_ESM.zip › SupplementalDataFiles/Figure 9/Figure 9(a2).tif]

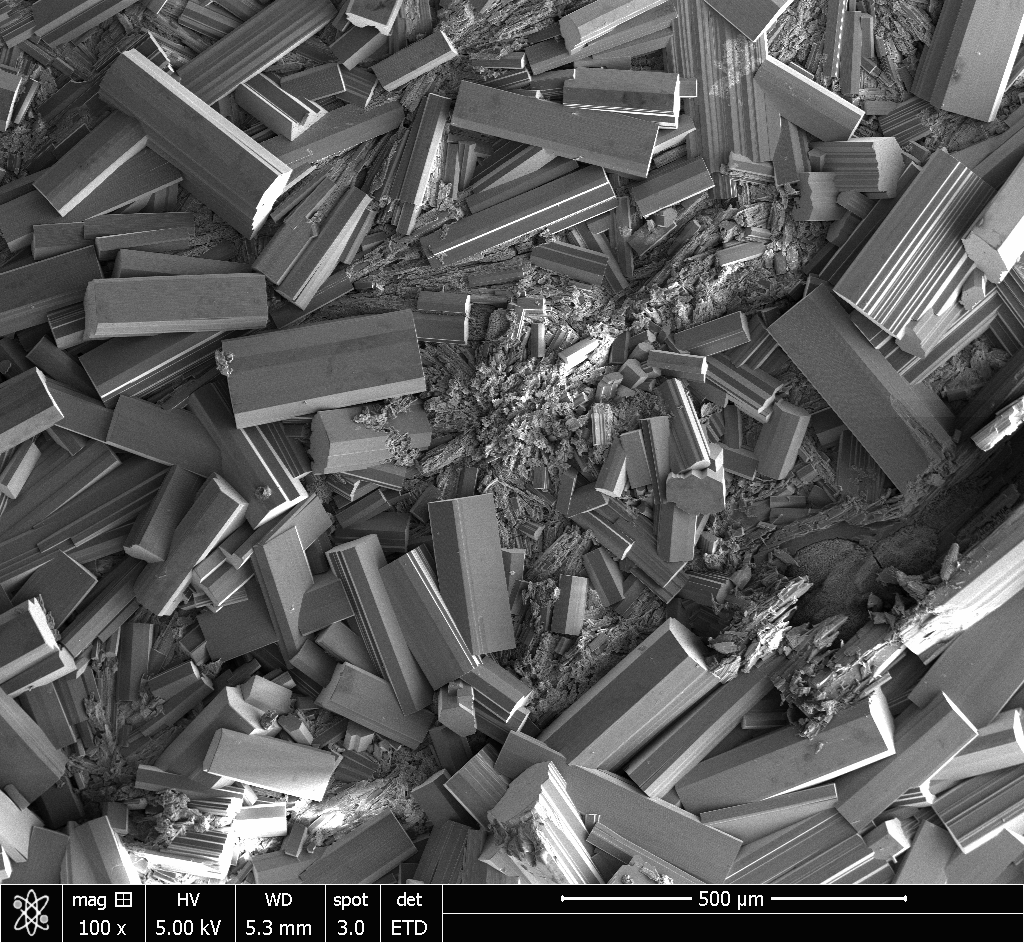

Supplement: Supplementary file 1 — Dataset for SREP-18-29489A [file 41598_2018_37123_MOESM1_ESM.zip › SupplementalDataFiles/Figure 9/Figure 9(b1).tif]

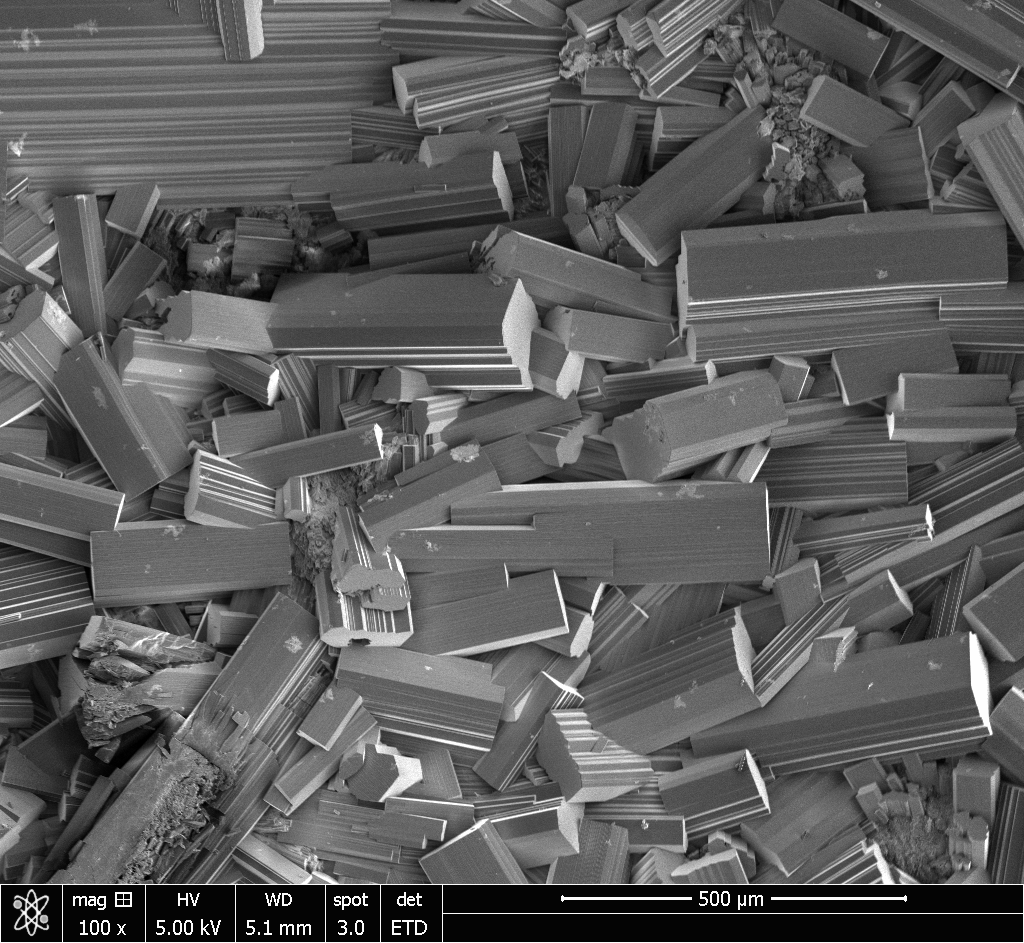

Supplement: Supplementary file 1 — Dataset for SREP-18-29489A [file 41598_2018_37123_MOESM1_ESM.zip › SupplementalDataFiles/Figure 9/Figure 9(b2).tif]
