# Supplementary figures and images for: Nano-to-Submicron Hydroxyapatite Coatings for Magnesium-based Bioresorbable Implants – Deposition, Characterization, Degradation, Mechanical Properties, and Cytocompatibility (part 1 of 2)
Source: Sci Rep. 2019 Jan 28;9:810. doi: 10.1038/s41598-018-37123-3 (PMC6349930; doi:10.1038/s41598-018-37123-3)

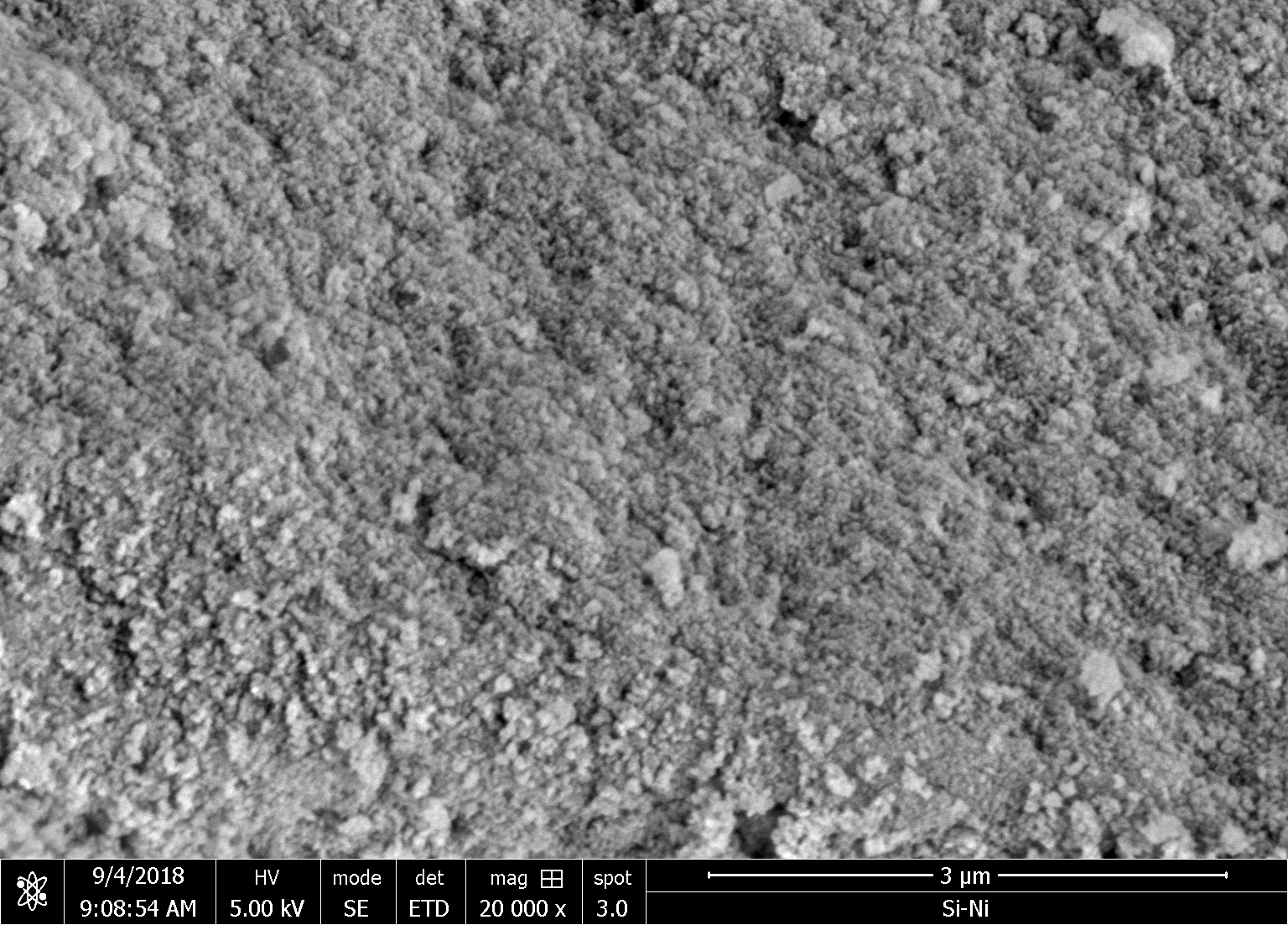

Supplement: Supplementary file 1 — Dataset for SREP-18-29489A [file 41598_2018_37123_MOESM1_ESM.zip › SupplementalDataFiles/Figure 1/Figure 1a, 1a'/Figure 1a1.tif]

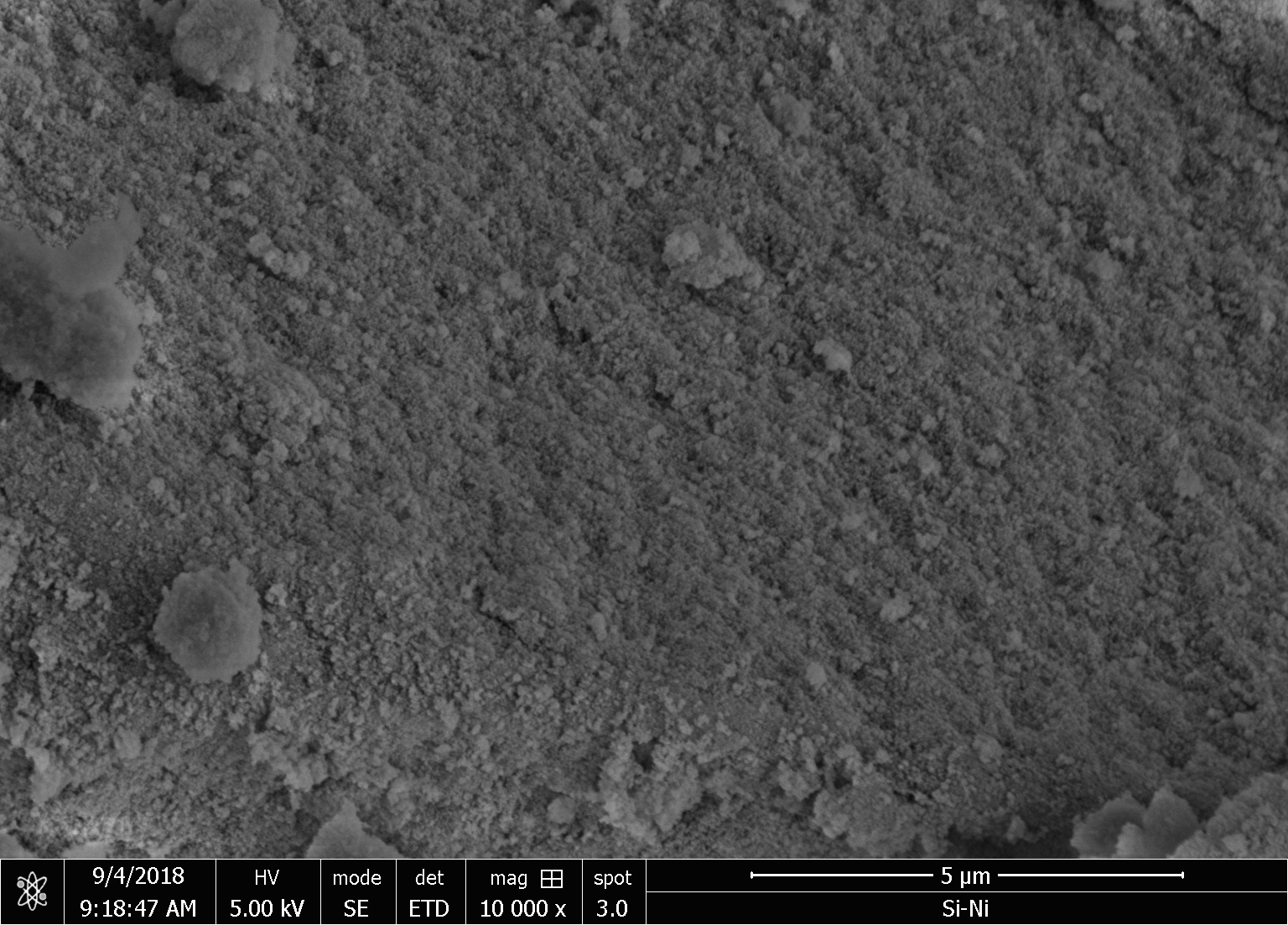

Supplement: Supplementary file 1 — Dataset for SREP-18-29489A [file 41598_2018_37123_MOESM1_ESM.zip › SupplementalDataFiles/Figure 1/Figure 1a, 1a'/Figure 1a1'.tif]

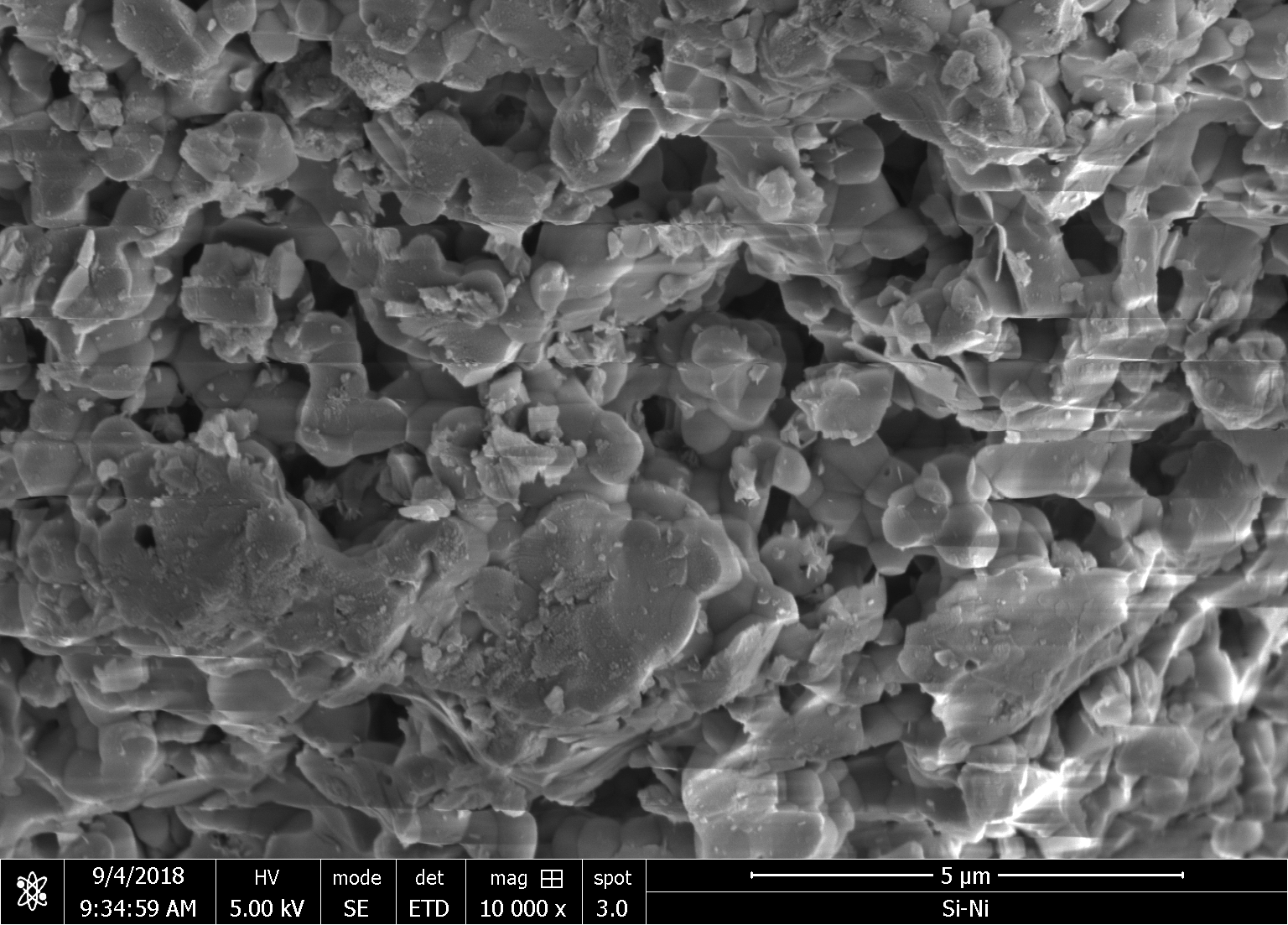

Supplement: Supplementary file 1 — Dataset for SREP-18-29489A [file 41598_2018_37123_MOESM1_ESM.zip › SupplementalDataFiles/Figure 1/Figure 1b, 1b'/Figure 1b1.tif]

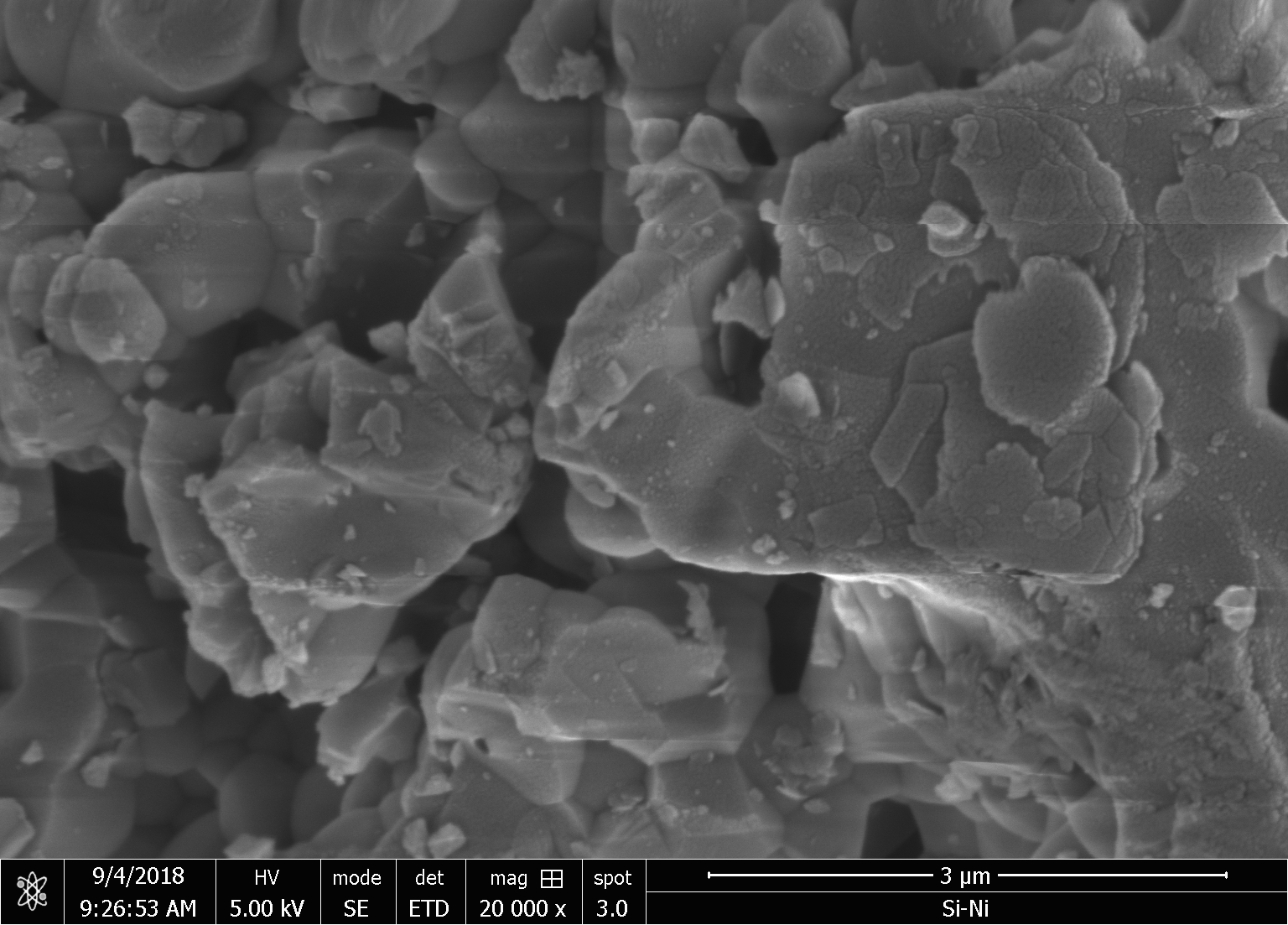

Supplement: Supplementary file 1 — Dataset for SREP-18-29489A [file 41598_2018_37123_MOESM1_ESM.zip › SupplementalDataFiles/Figure 1/Figure 1b, 1b'/Figure 1b1'.tif]

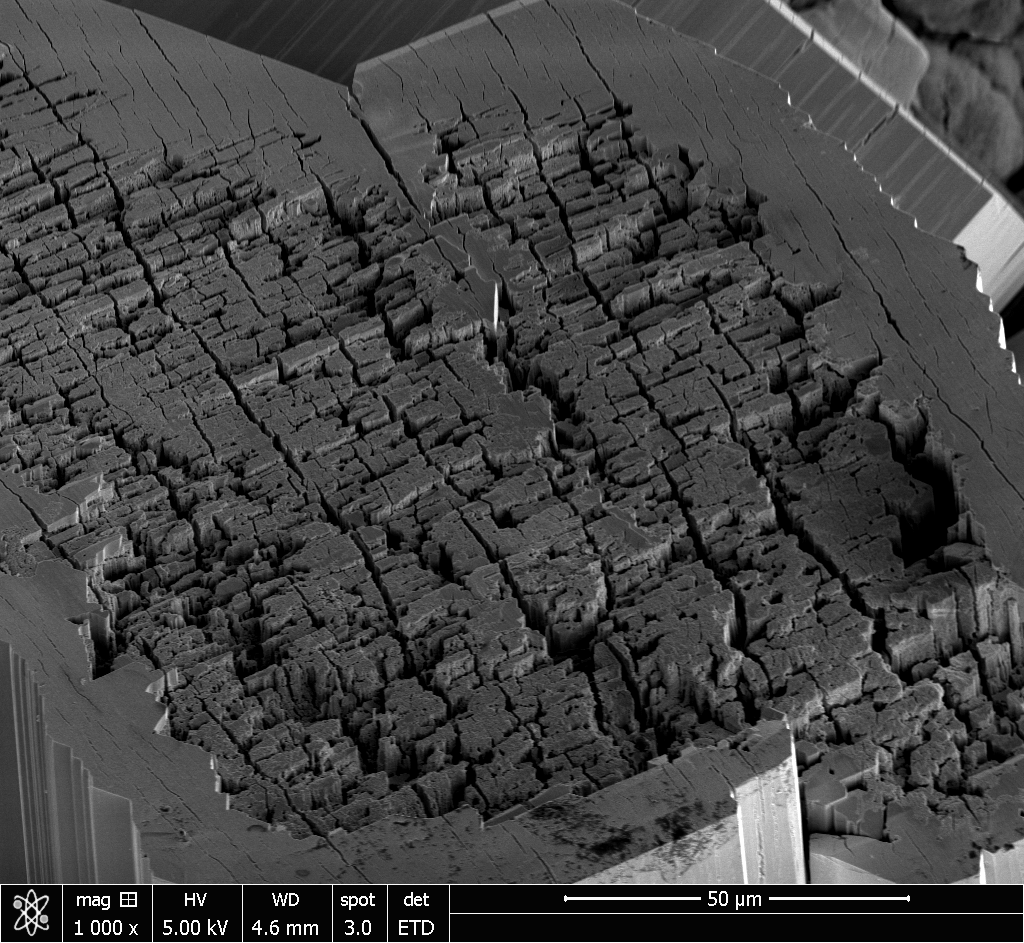

Supplement: Supplementary file 1 — Dataset for SREP-18-29489A [file 41598_2018_37123_MOESM1_ESM.zip › SupplementalDataFiles/Figure 10/Figure 10 (a1).tif]

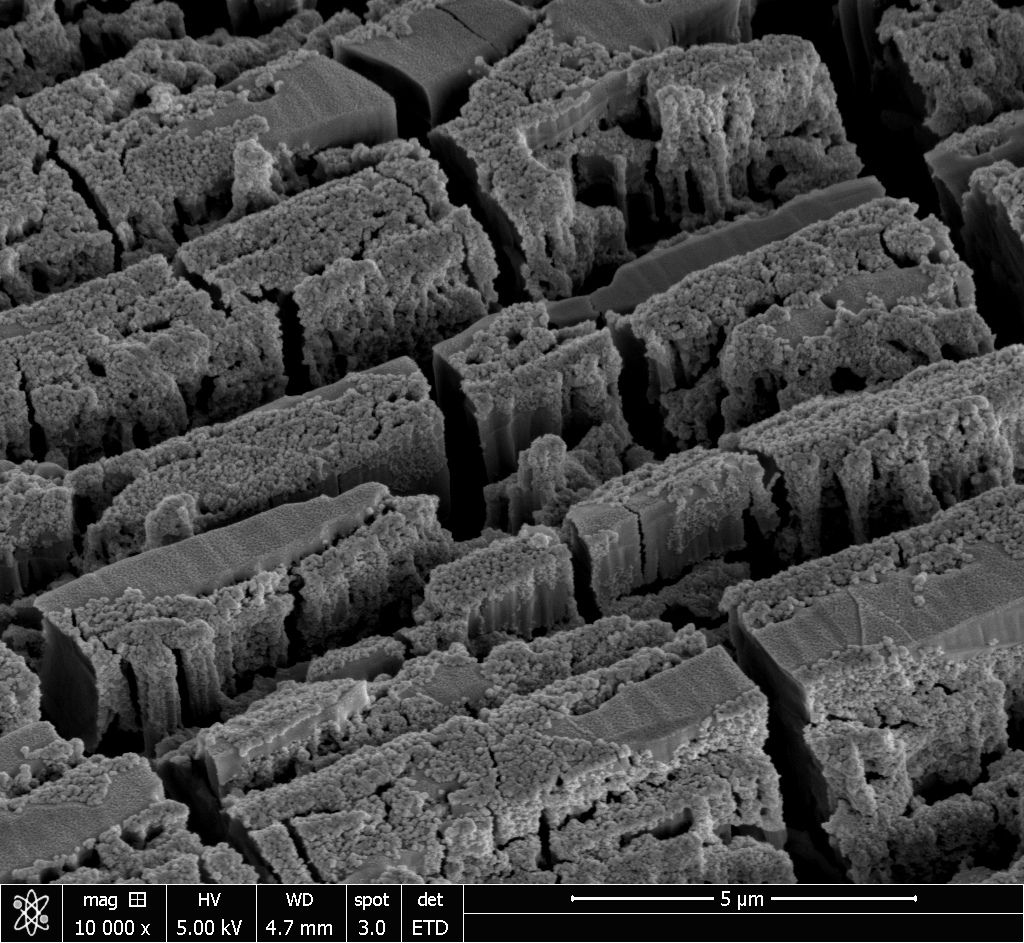

Supplement: Supplementary file 1 — Dataset for SREP-18-29489A [file 41598_2018_37123_MOESM1_ESM.zip › SupplementalDataFiles/Figure 10/Figure 10 (a2).tif]

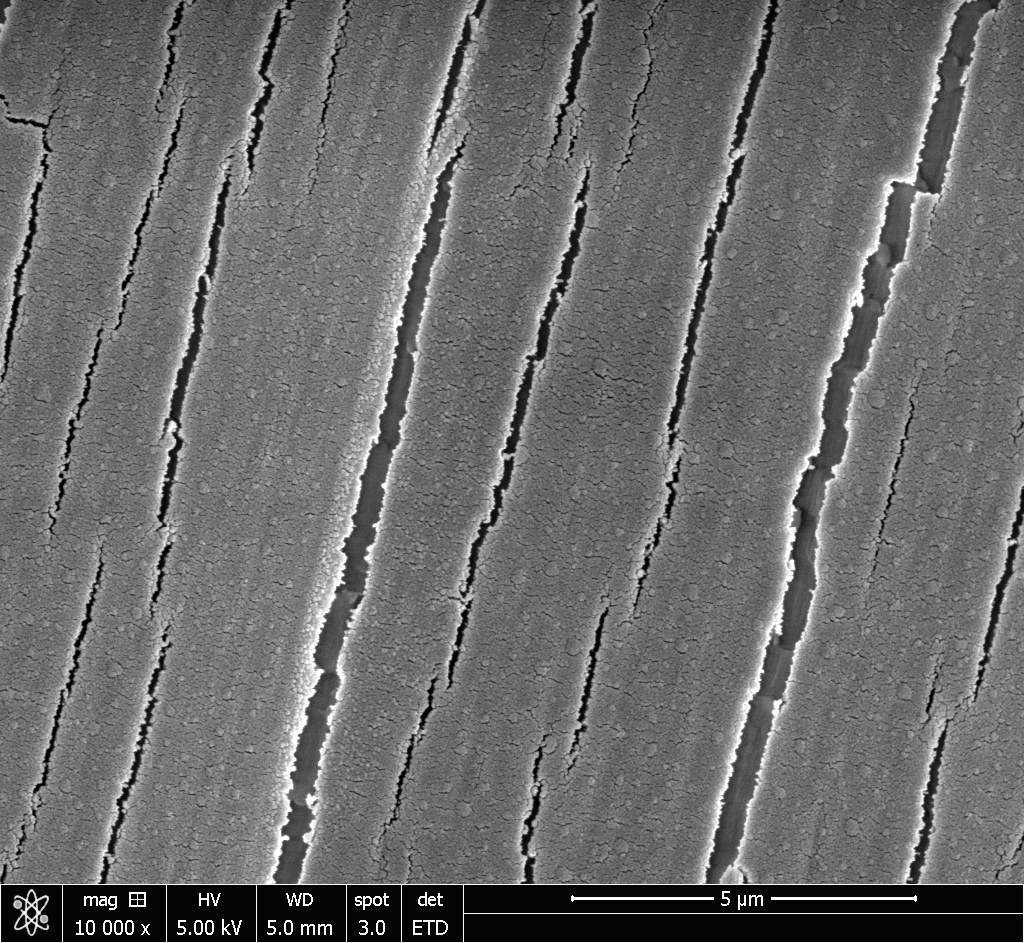

Supplement: Supplementary file 1 — Dataset for SREP-18-29489A [file 41598_2018_37123_MOESM1_ESM.zip › SupplementalDataFiles/Figure 10/Figure 10 (a3).tif]

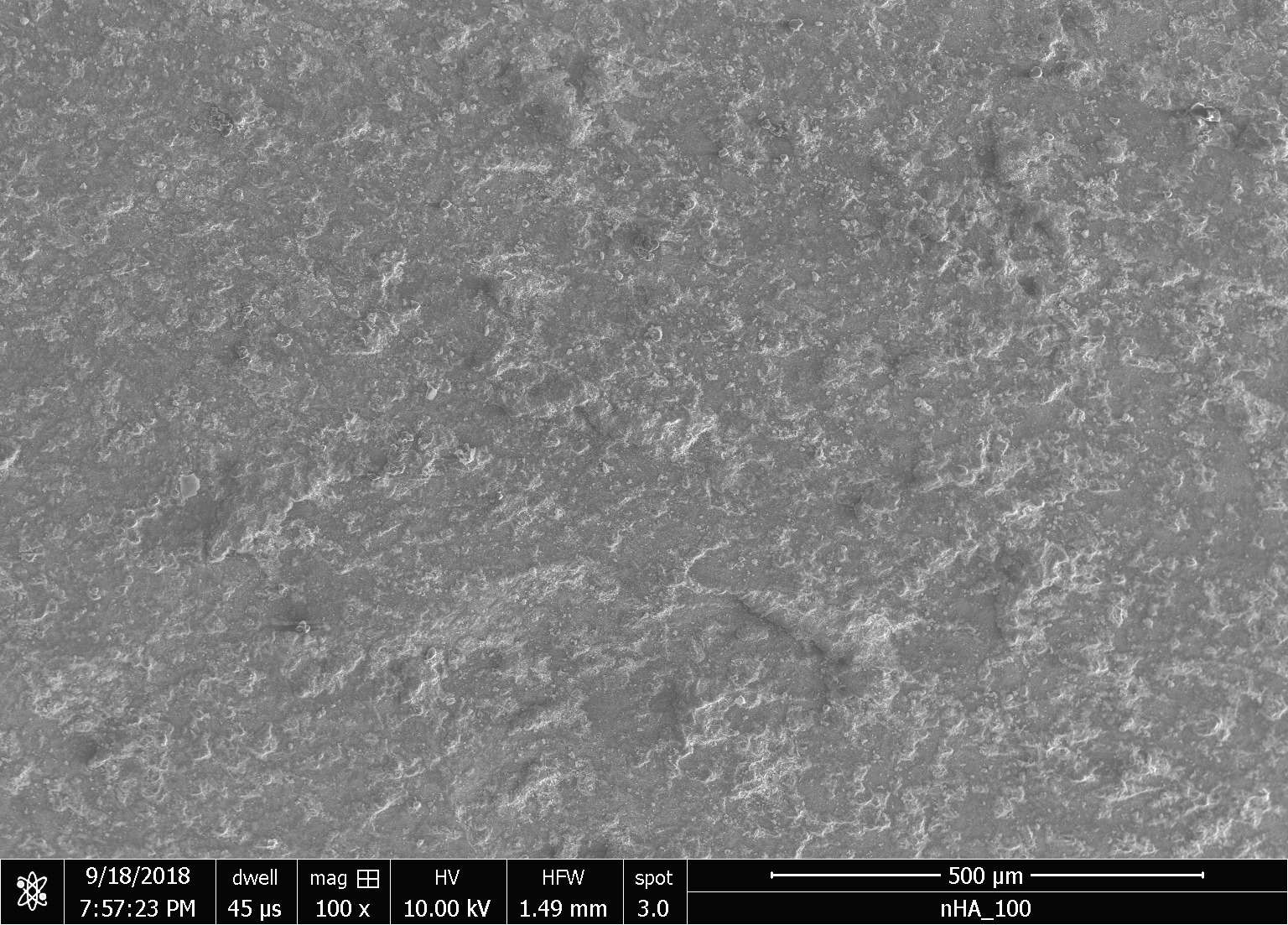

Supplement: Supplementary file 1 — Dataset for SREP-18-29489A [file 41598_2018_37123_MOESM1_ESM.zip › SupplementalDataFiles/Figure 2/Figure 2a1, a1'/a1.tif]

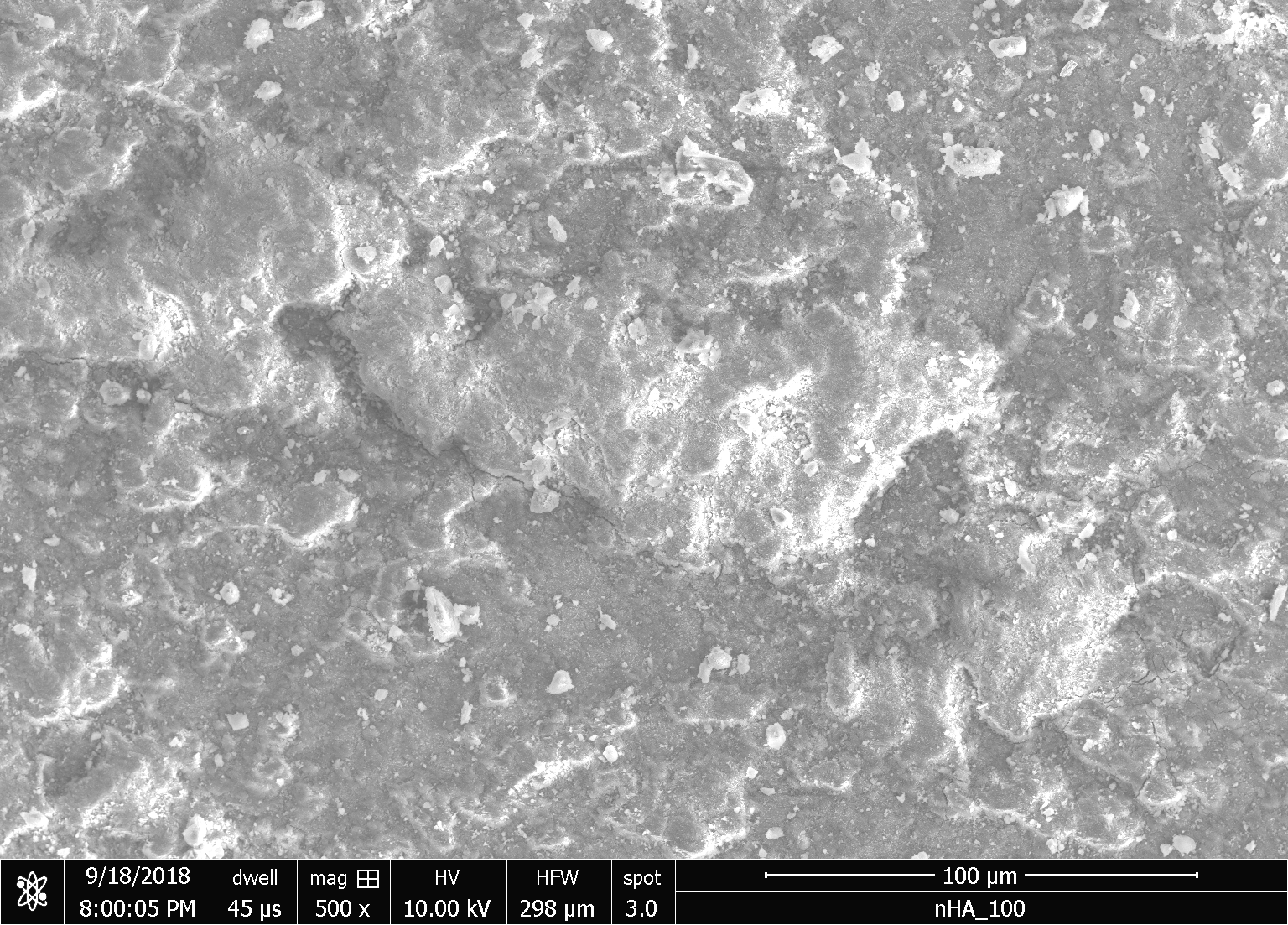

Supplement: Supplementary file 1 — Dataset for SREP-18-29489A [file 41598_2018_37123_MOESM1_ESM.zip › SupplementalDataFiles/Figure 2/Figure 2a1, a1'/a1'.tif]

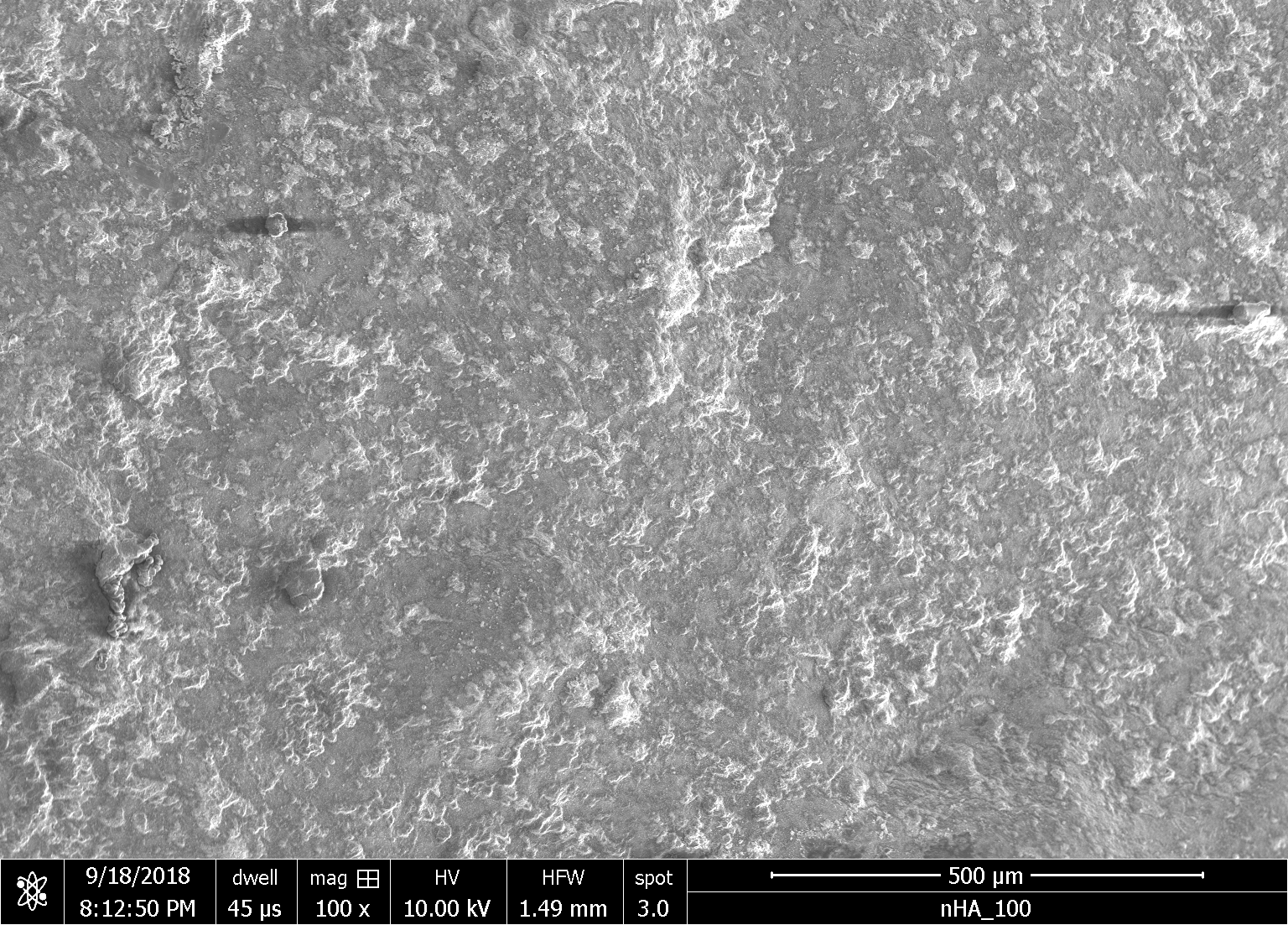

Supplement: Supplementary file 1 — Dataset for SREP-18-29489A [file 41598_2018_37123_MOESM1_ESM.zip › SupplementalDataFiles/Figure 2/Figure 2a2, a2'/a2.tif]

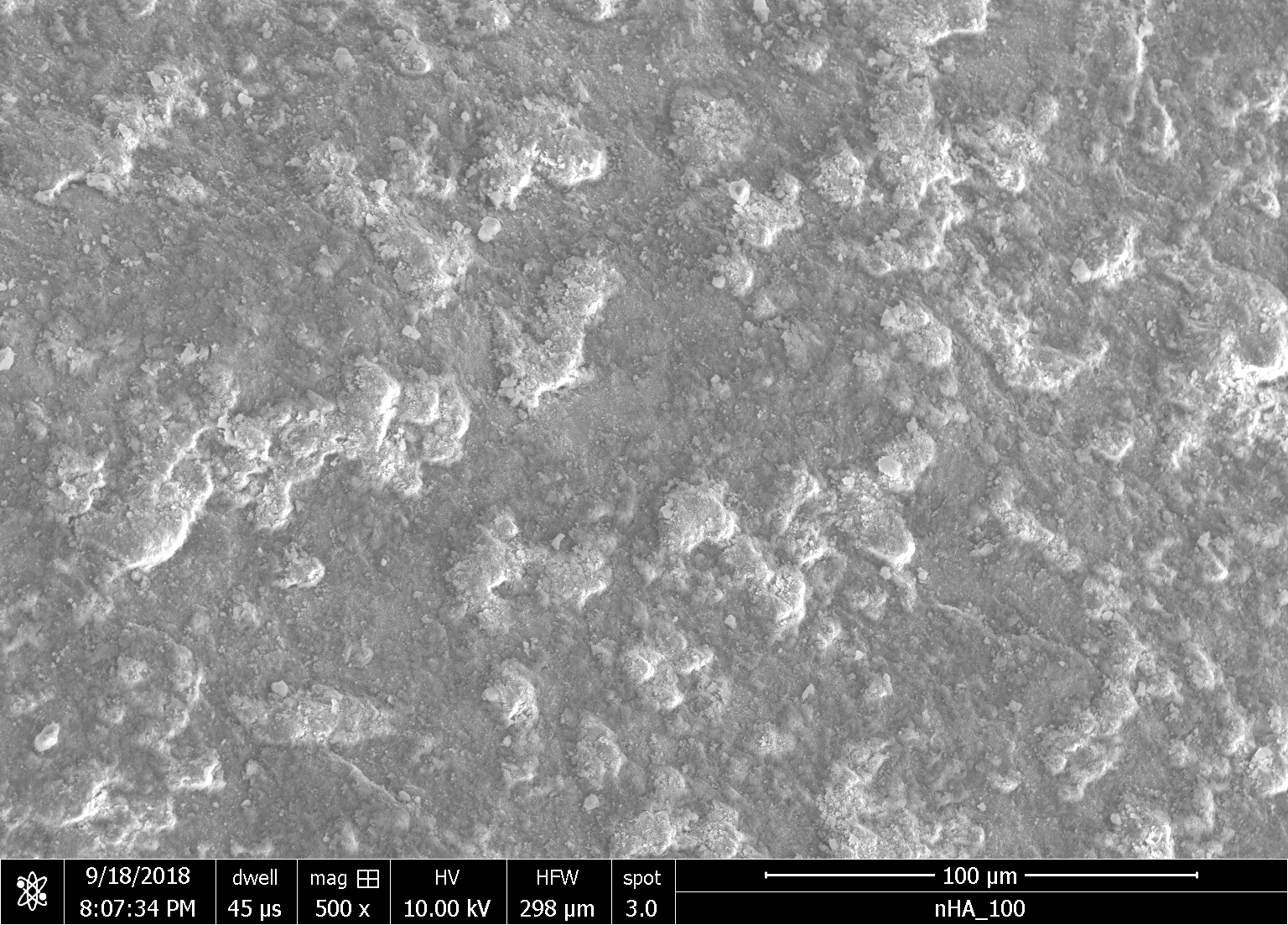

Supplement: Supplementary file 1 — Dataset for SREP-18-29489A [file 41598_2018_37123_MOESM1_ESM.zip › SupplementalDataFiles/Figure 2/Figure 2a2, a2'/a2'.tif]

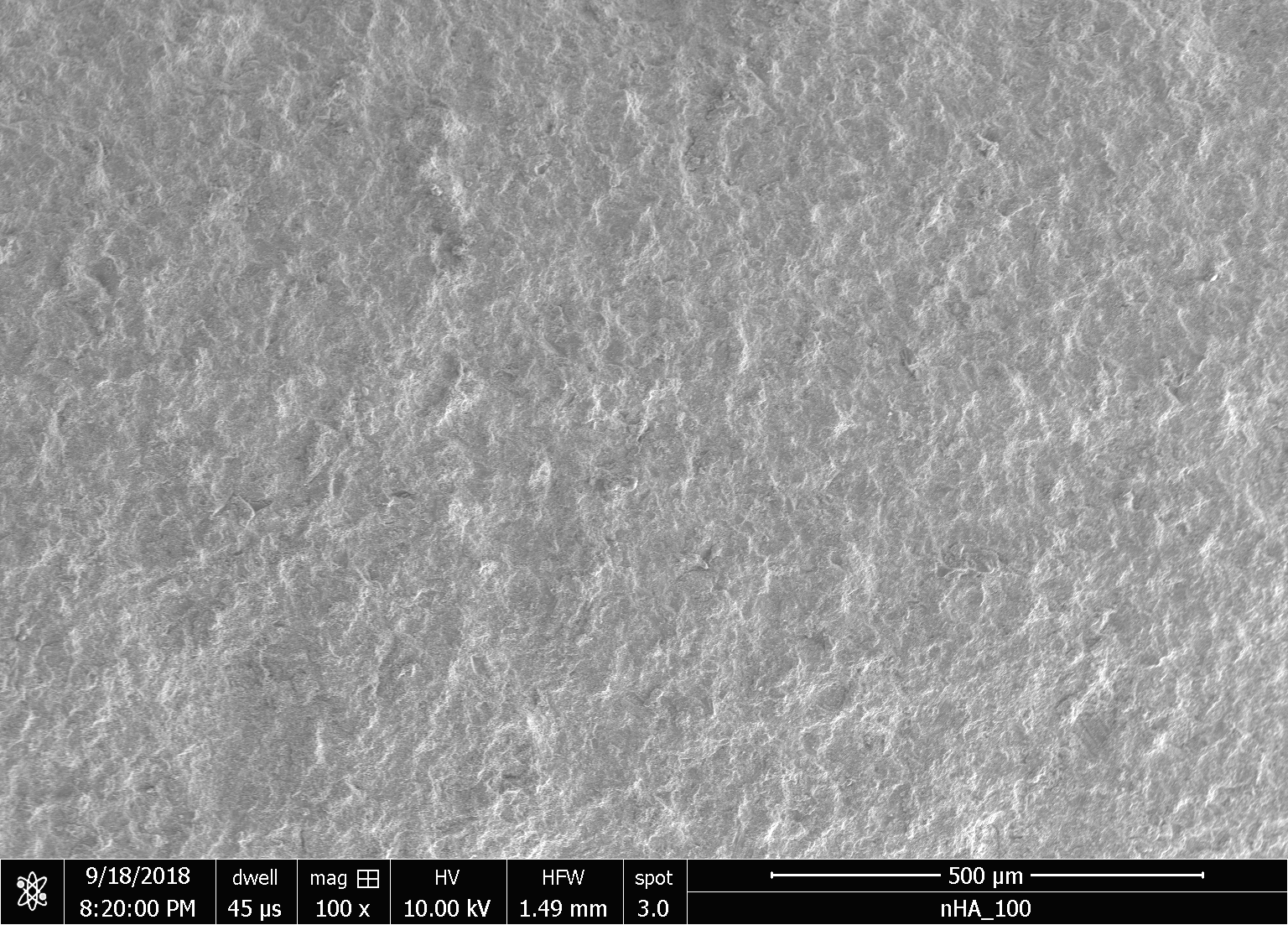

Supplement: Supplementary file 1 — Dataset for SREP-18-29489A [file 41598_2018_37123_MOESM1_ESM.zip › SupplementalDataFiles/Figure 2/Figure 2b1, b1'/b1.tif]

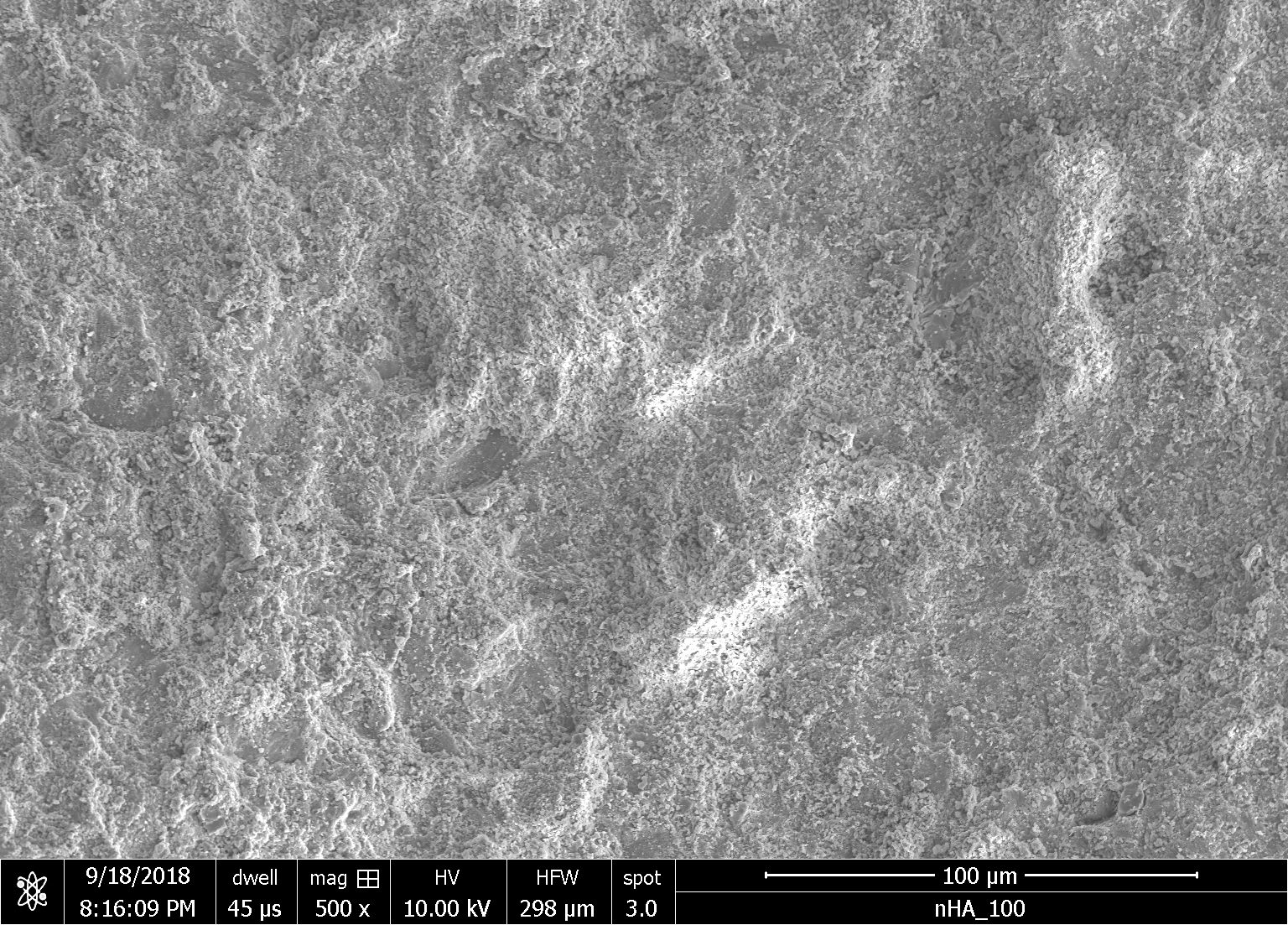

Supplement: Supplementary file 1 — Dataset for SREP-18-29489A [file 41598_2018_37123_MOESM1_ESM.zip › SupplementalDataFiles/Figure 2/Figure 2b1, b1'/b1'.tif]

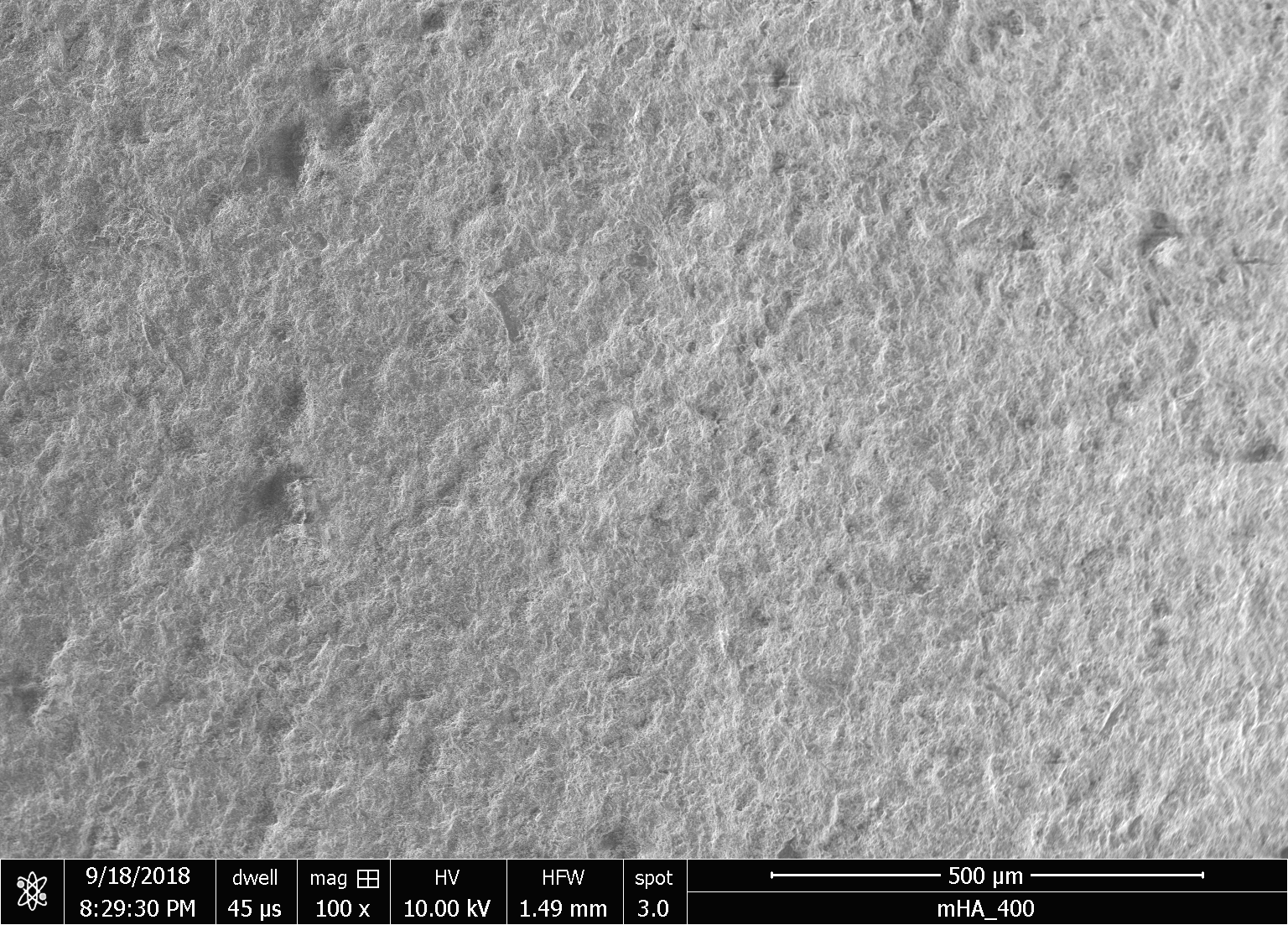

Supplement: Supplementary file 1 — Dataset for SREP-18-29489A [file 41598_2018_37123_MOESM1_ESM.zip › SupplementalDataFiles/Figure 2/Figure 2b2, b2'/b2.tif]

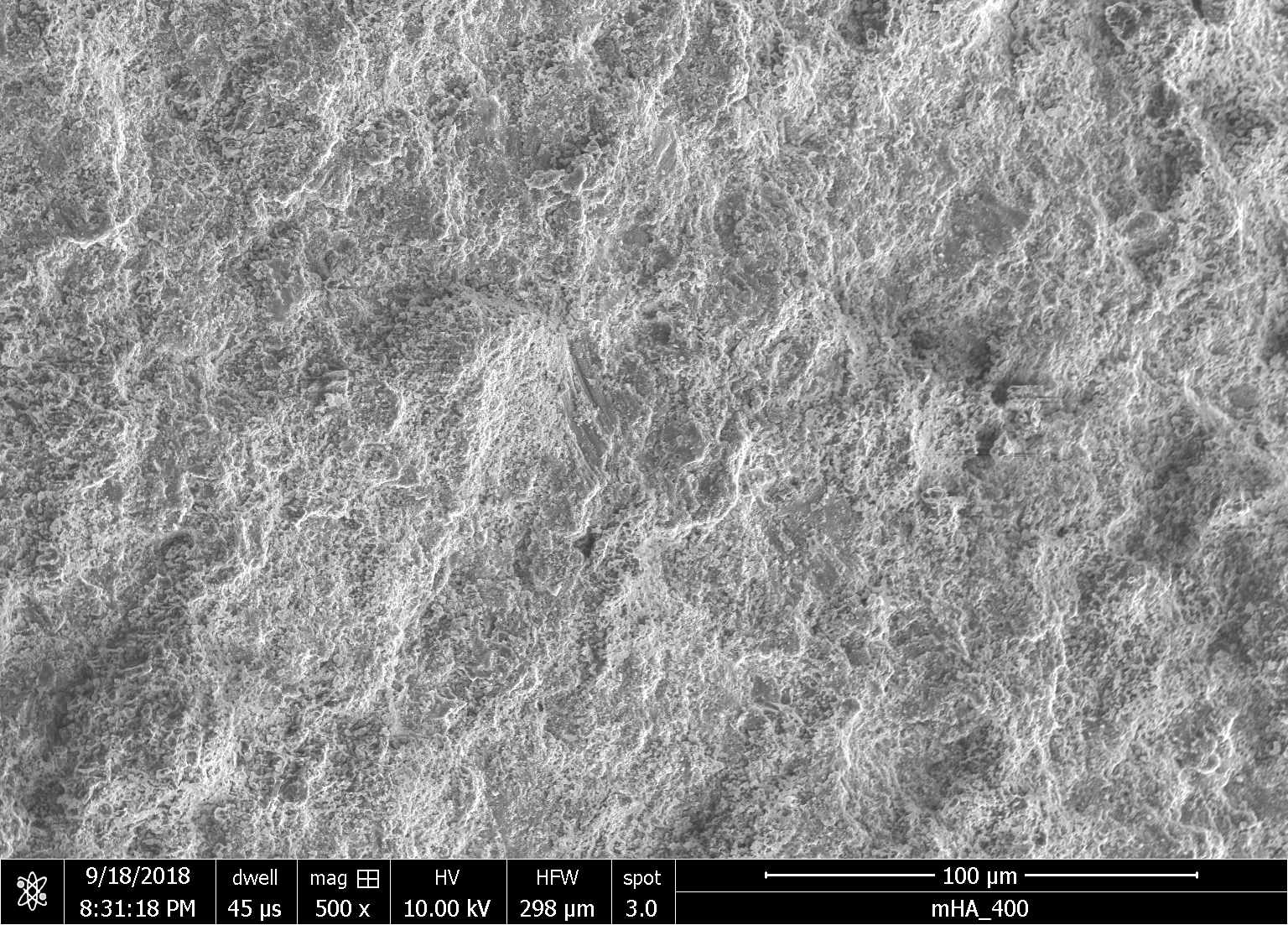

Supplement: Supplementary file 1 — Dataset for SREP-18-29489A [file 41598_2018_37123_MOESM1_ESM.zip › SupplementalDataFiles/Figure 2/Figure 2b2, b2'/b2'.tif]

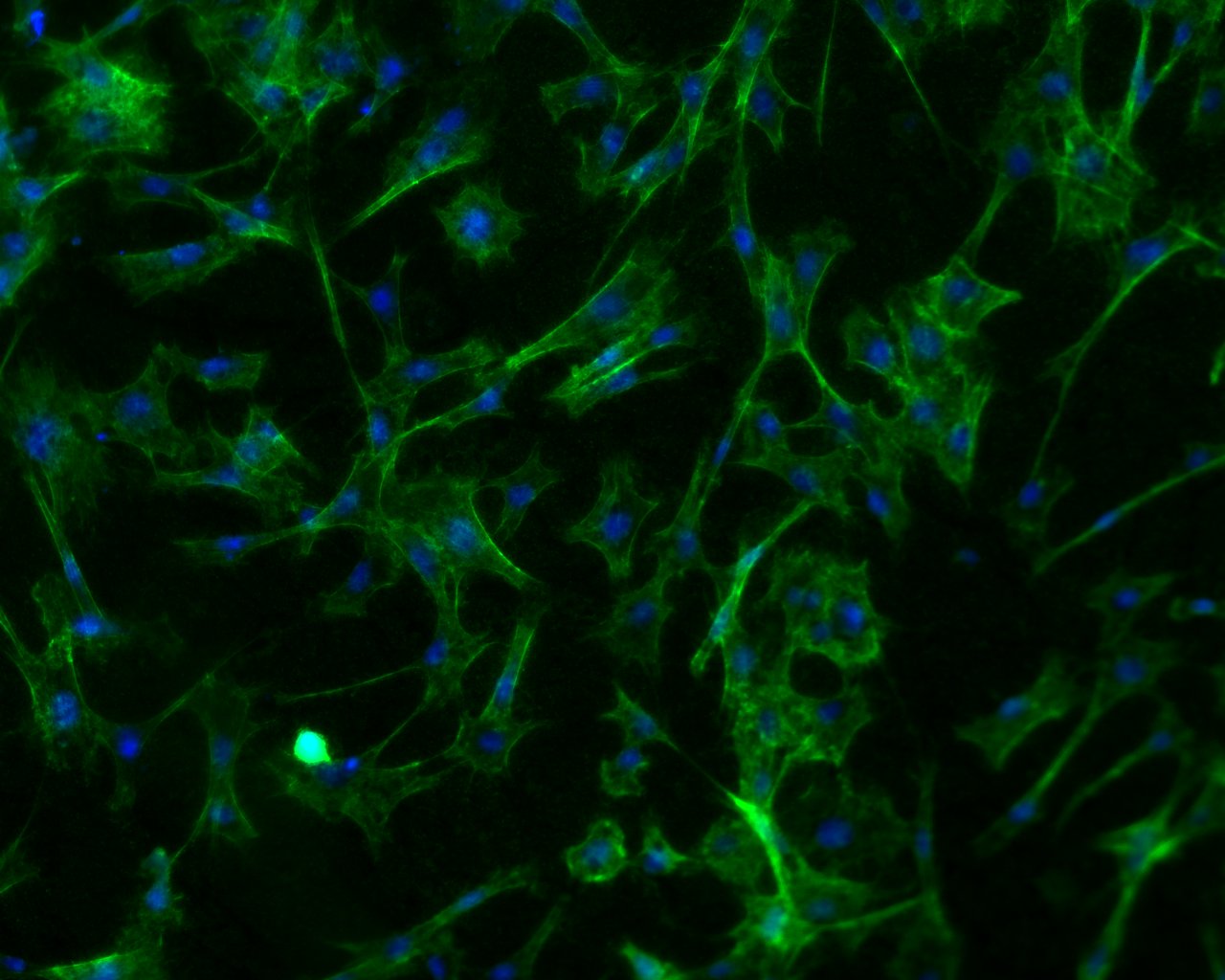

Supplement: Supplementary file 1 — Dataset for SREP-18-29489A [file 41598_2018_37123_MOESM1_ESM.zip › SupplementalDataFiles/Figure 20/Figure 20(a)/Mg_direct contact.jpg]

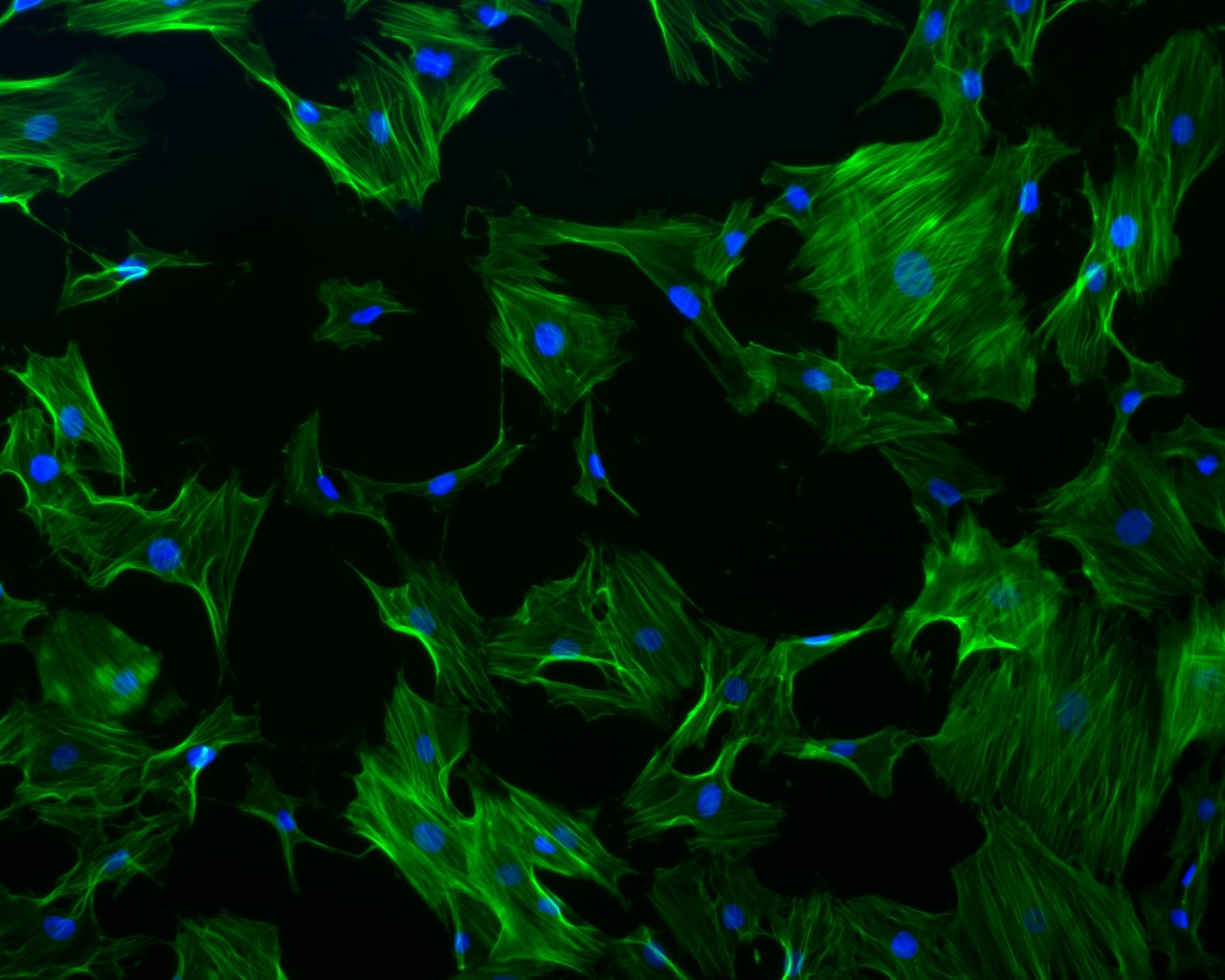

Supplement: Supplementary file 1 — Dataset for SREP-18-29489A [file 41598_2018_37123_MOESM1_ESM.zip › SupplementalDataFiles/Figure 20/Figure 20(a)/Mg_indirect contact.jpg]

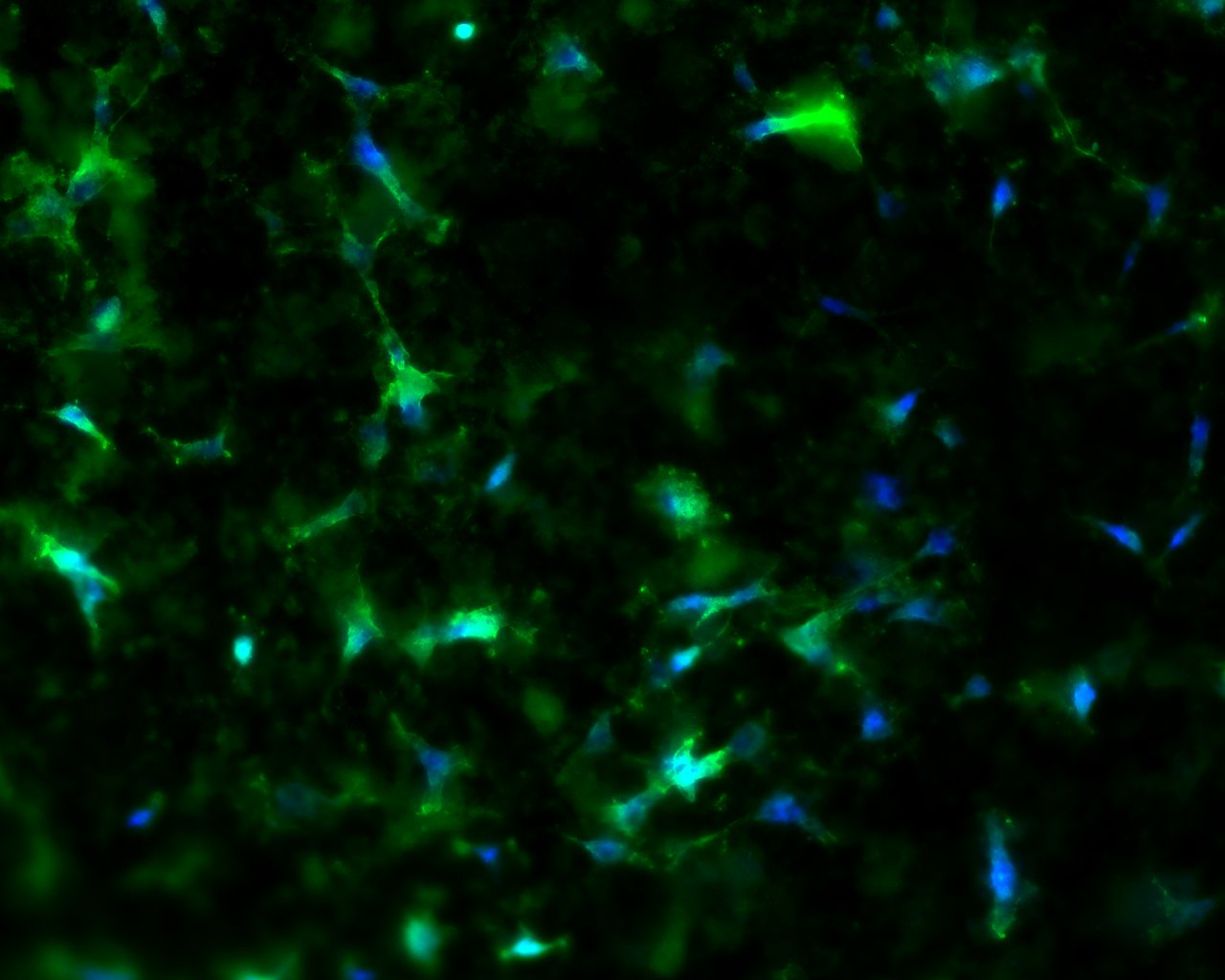

Supplement: Supplementary file 1 — Dataset for SREP-18-29489A [file 41598_2018_37123_MOESM1_ESM.zip › SupplementalDataFiles/Figure 20/Figure 20(a)/mHA_400_direct contact.jpg]

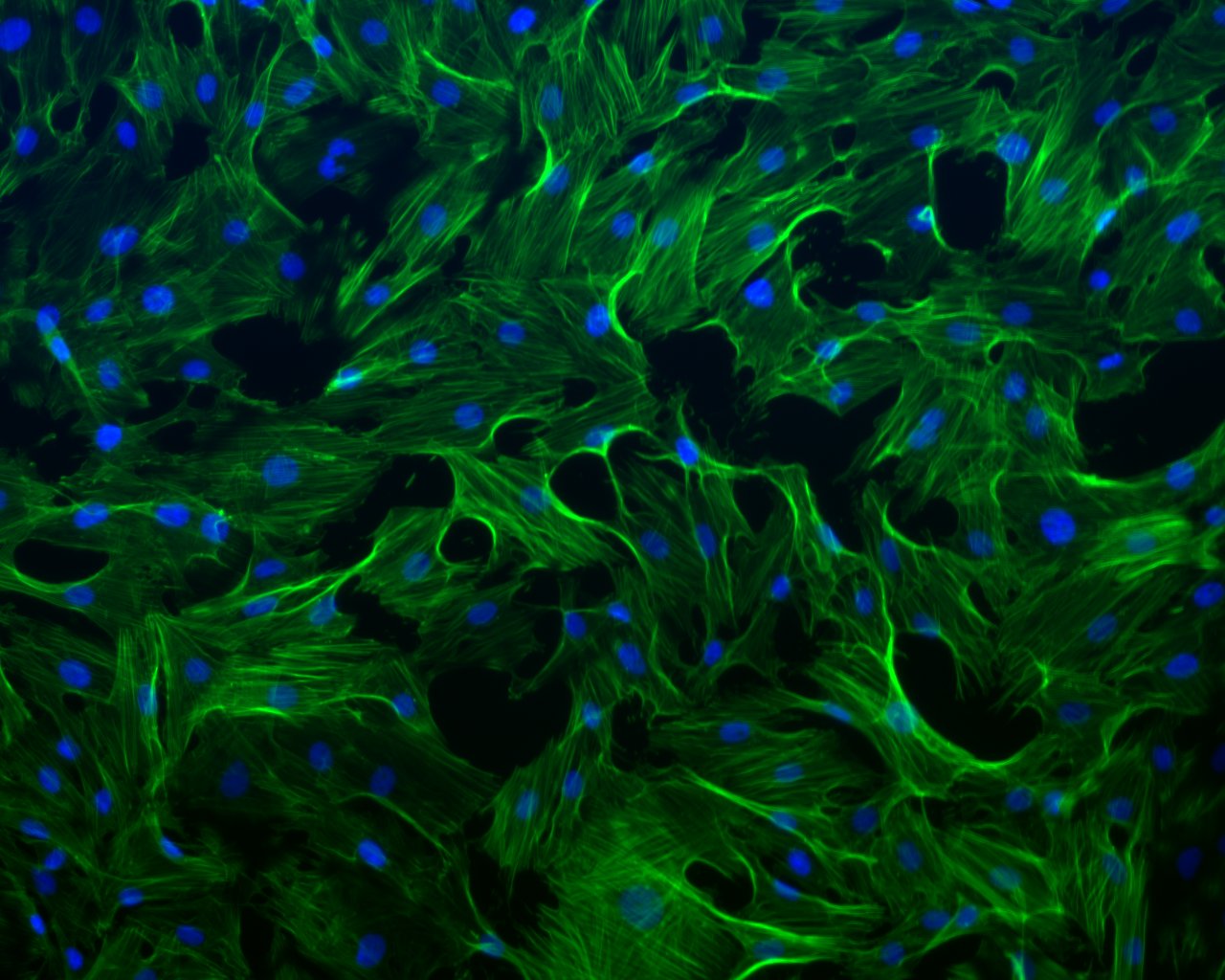

Supplement: Supplementary file 1 — Dataset for SREP-18-29489A [file 41598_2018_37123_MOESM1_ESM.zip › SupplementalDataFiles/Figure 20/Figure 20(a)/mHA_400_indirect contact.jpg]

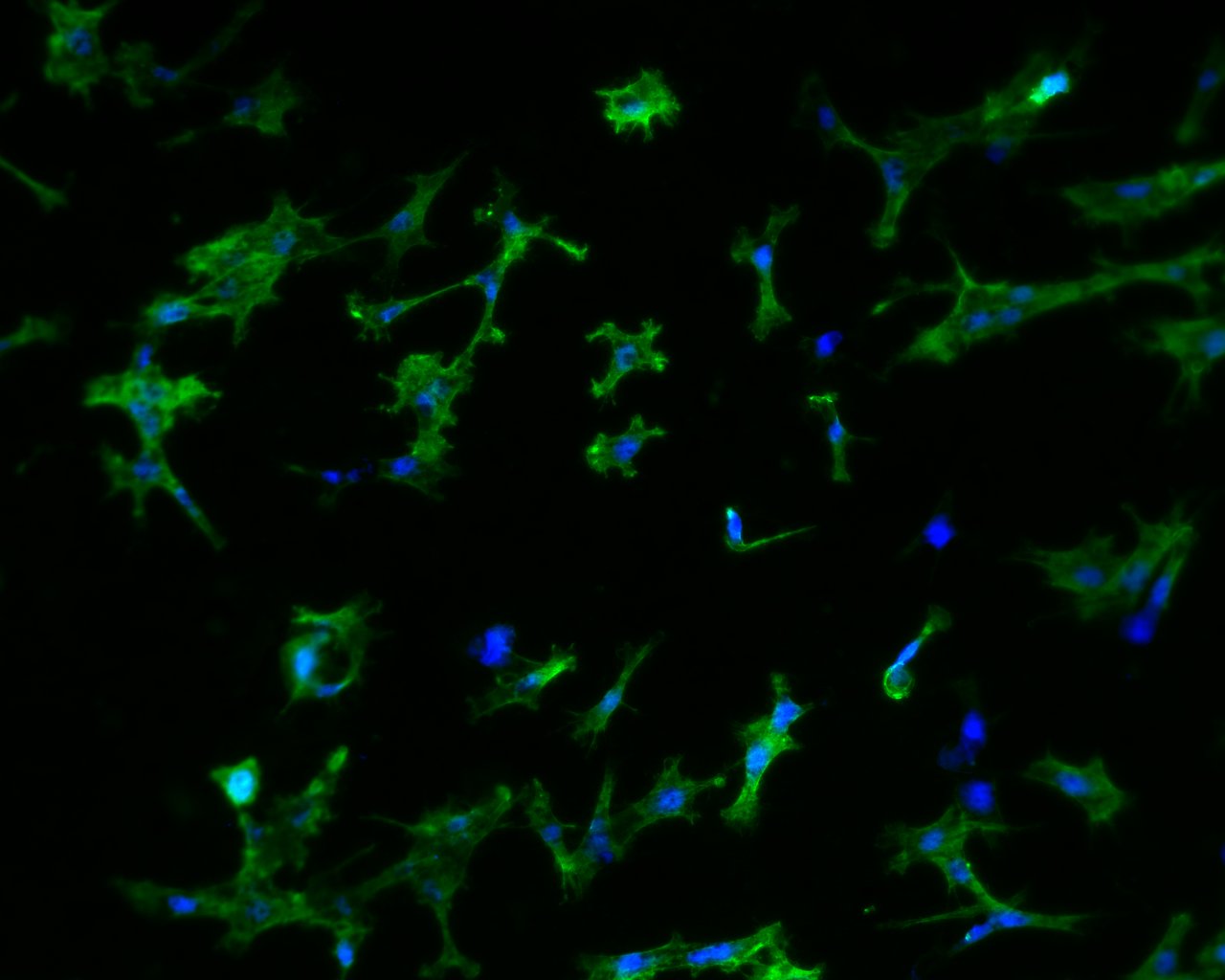

Supplement: Supplementary file 1 — Dataset for SREP-18-29489A [file 41598_2018_37123_MOESM1_ESM.zip › SupplementalDataFiles/Figure 20/Figure 20(a)/nHA_400_direct contact.jpg]

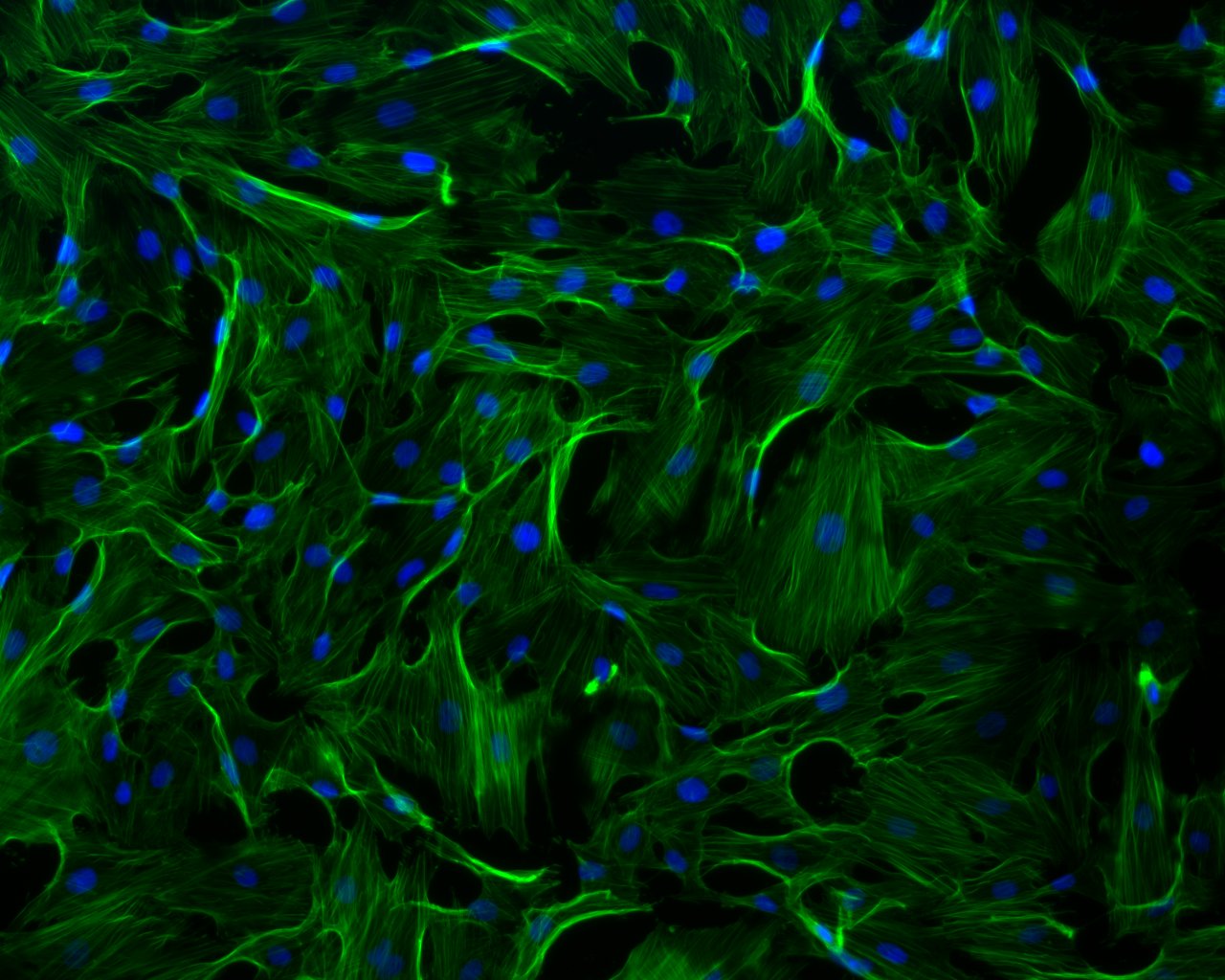

Supplement: Supplementary file 1 — Dataset for SREP-18-29489A [file 41598_2018_37123_MOESM1_ESM.zip › SupplementalDataFiles/Figure 20/Figure 20(a)/nHA_400_indirect contact.jpg]

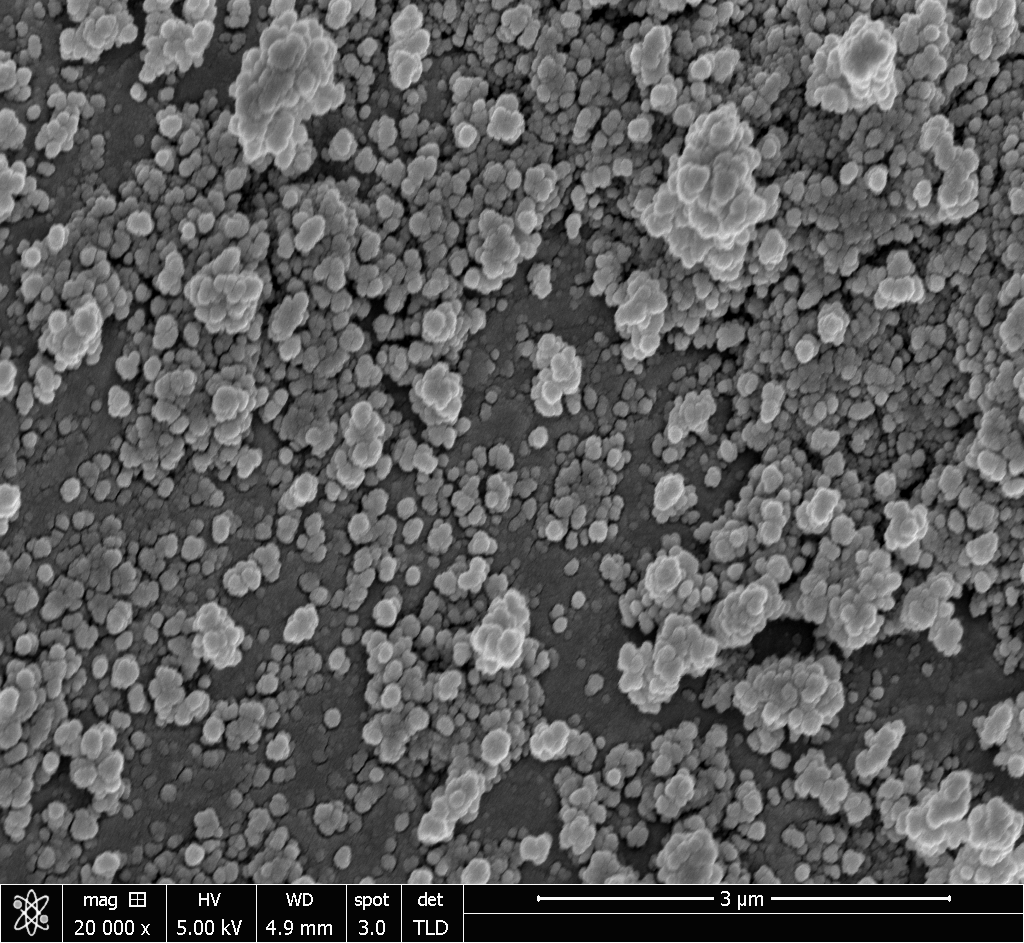

Supplement: Supplementary file 1 — Dataset for SREP-18-29489A [file 41598_2018_37123_MOESM1_ESM.zip › SupplementalDataFiles/Figure 3/Figure 3(a1).tif]

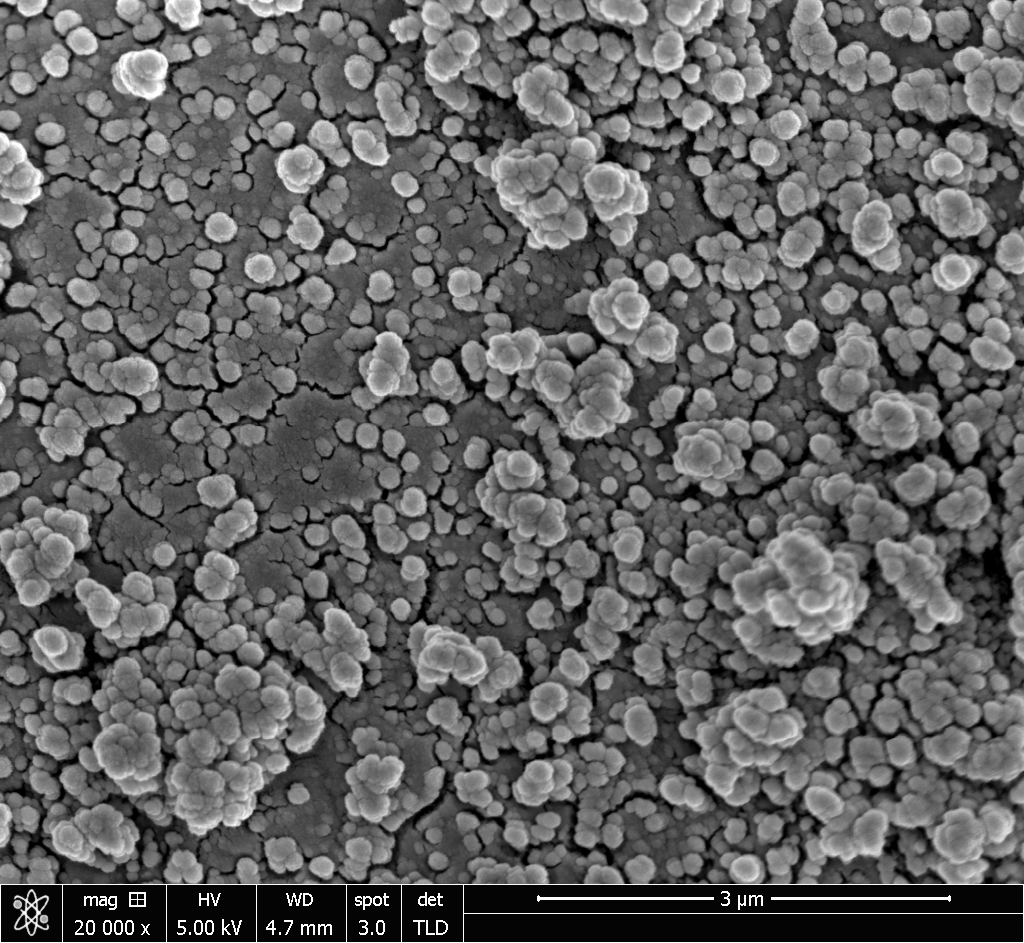

Supplement: Supplementary file 1 — Dataset for SREP-18-29489A [file 41598_2018_37123_MOESM1_ESM.zip › SupplementalDataFiles/Figure 3/Figure 3(a2).tif]

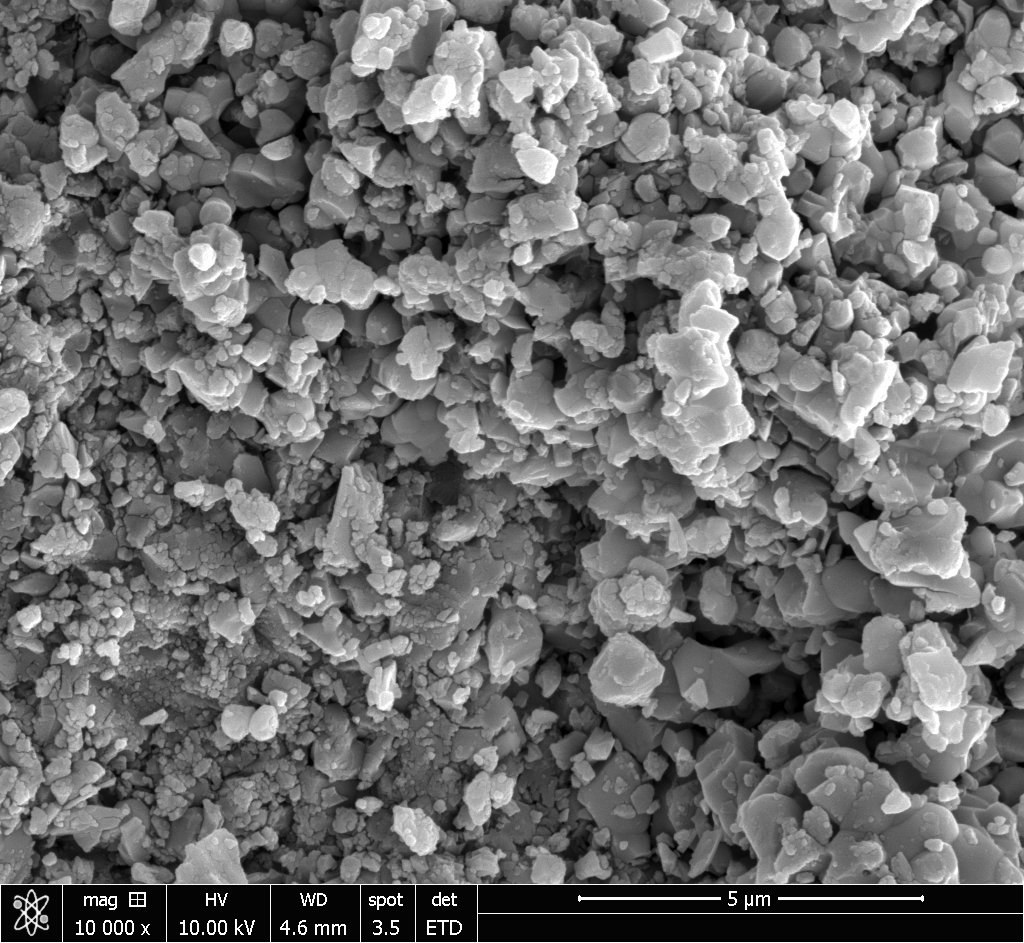

Supplement: Supplementary file 1 — Dataset for SREP-18-29489A [file 41598_2018_37123_MOESM1_ESM.zip › SupplementalDataFiles/Figure 3/Figure 3(b1).tif]

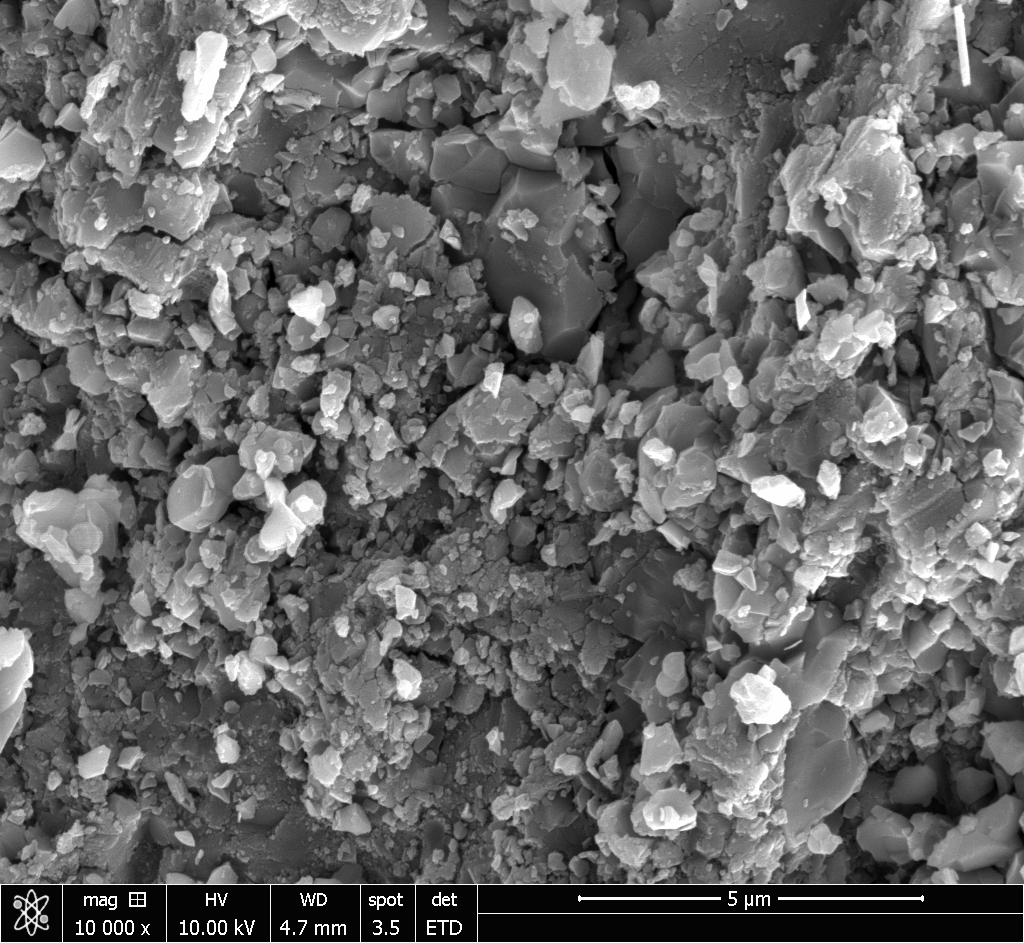

Supplement: Supplementary file 1 — Dataset for SREP-18-29489A [file 41598_2018_37123_MOESM1_ESM.zip › SupplementalDataFiles/Figure 3/Figure 3(b2).tif]

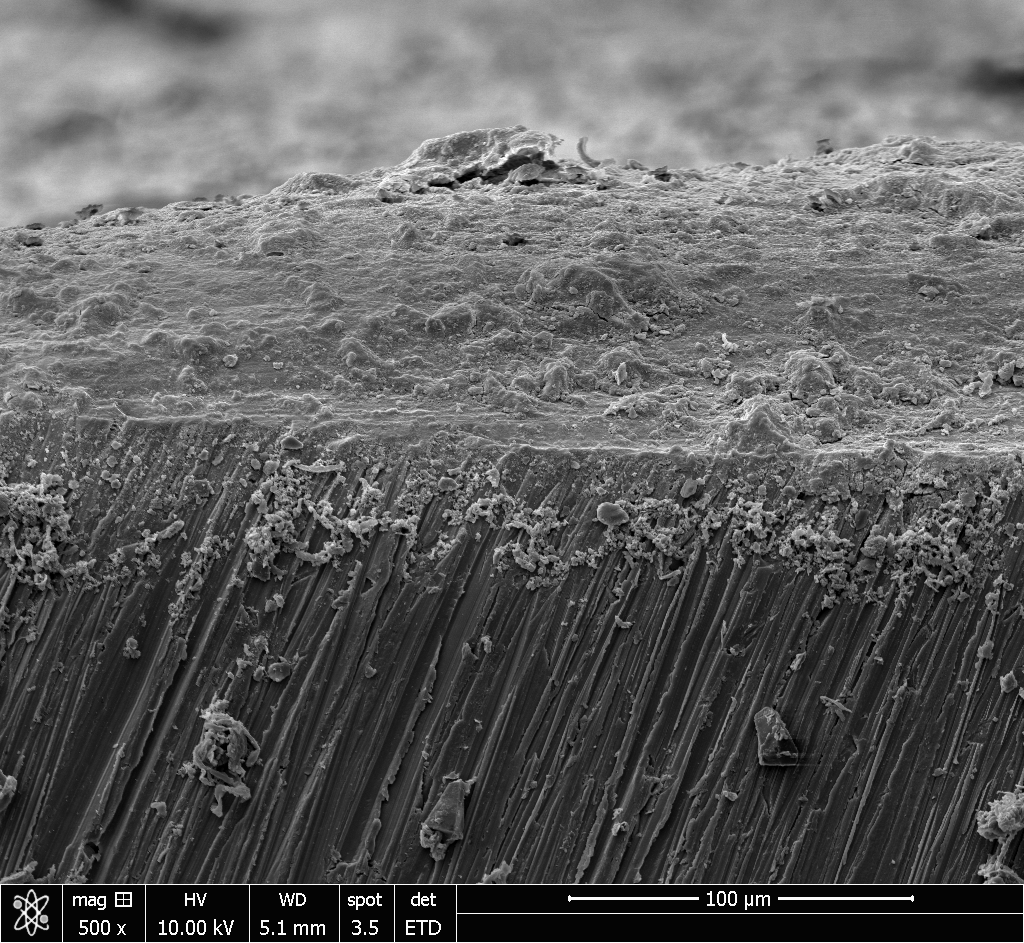

Supplement: Supplementary file 1 — Dataset for SREP-18-29489A [file 41598_2018_37123_MOESM1_ESM.zip › SupplementalDataFiles/Figure 5/Figure 5(a1).tif]

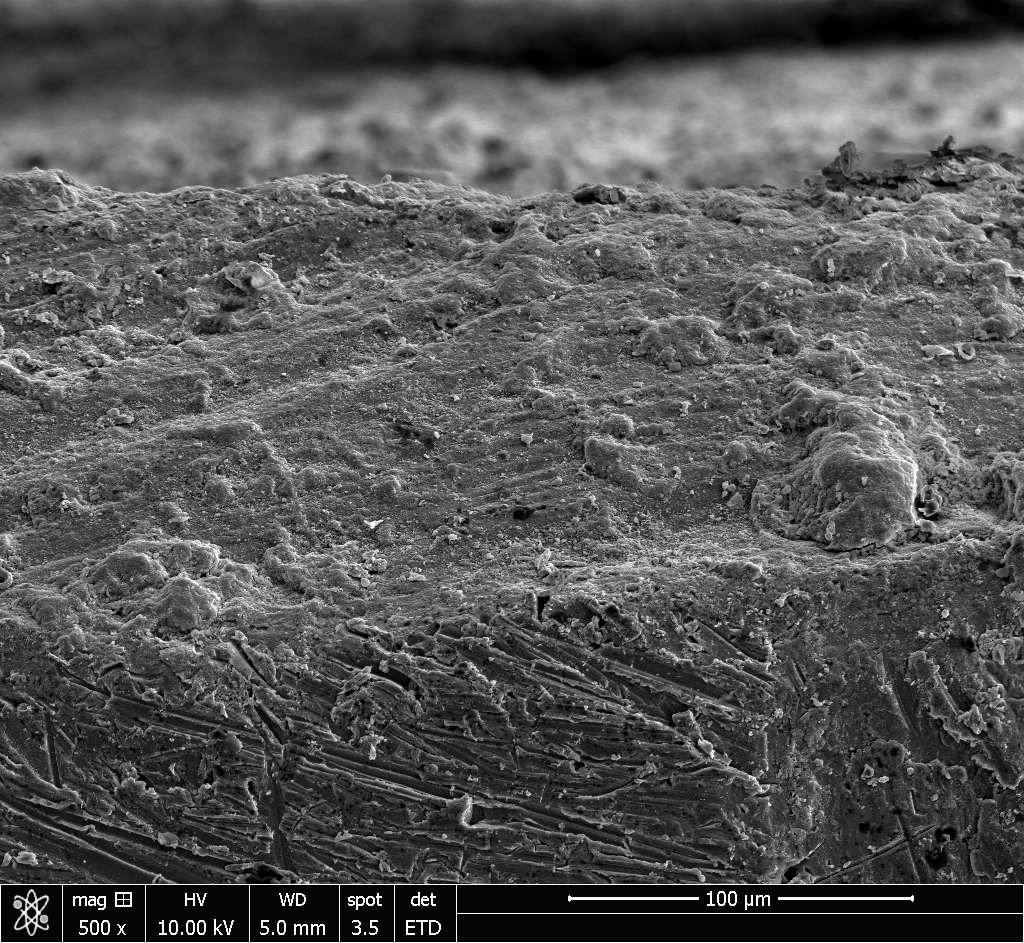

Supplement: Supplementary file 1 — Dataset for SREP-18-29489A [file 41598_2018_37123_MOESM1_ESM.zip › SupplementalDataFiles/Figure 5/Figure 5(a2).tif]

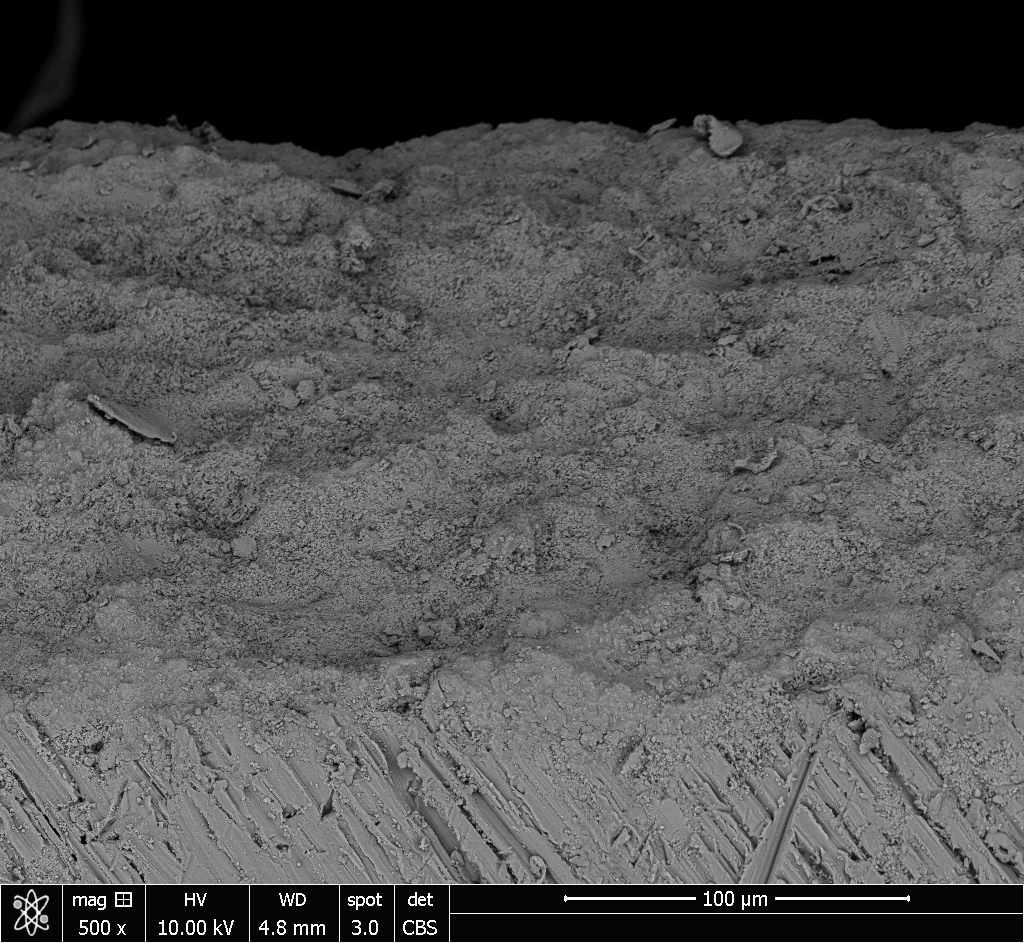

Supplement: Supplementary file 1 — Dataset for SREP-18-29489A [file 41598_2018_37123_MOESM1_ESM.zip › SupplementalDataFiles/Figure 5/Figure 5(b1).tif]

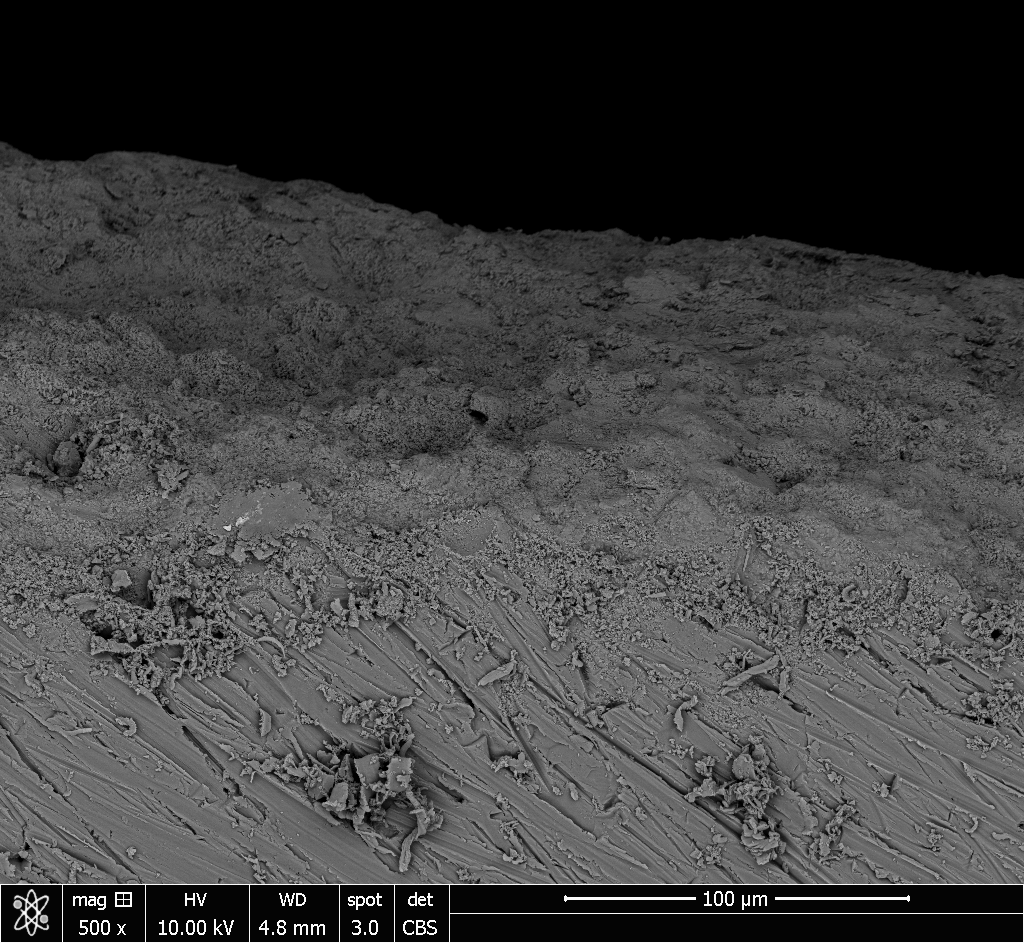

Supplement: Supplementary file 1 — Dataset for SREP-18-29489A [file 41598_2018_37123_MOESM1_ESM.zip › SupplementalDataFiles/Figure 5/Figure 5(b2).tif]

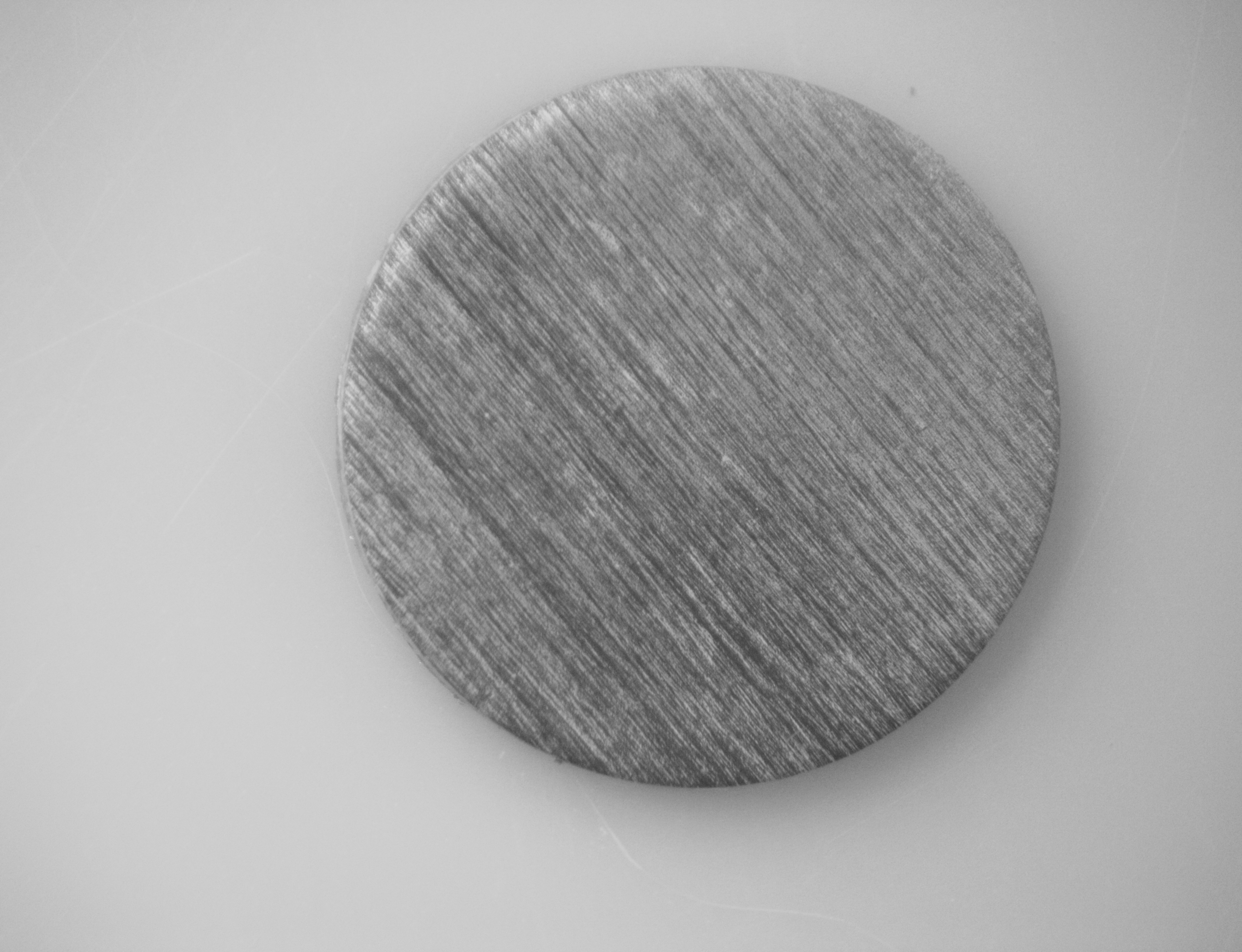

Supplement: Supplementary file 1 — Dataset for SREP-18-29489A [file 41598_2018_37123_MOESM1_ESM.zip › SupplementalDataFiles/Figure 6/0 hour/Mg.jpg]

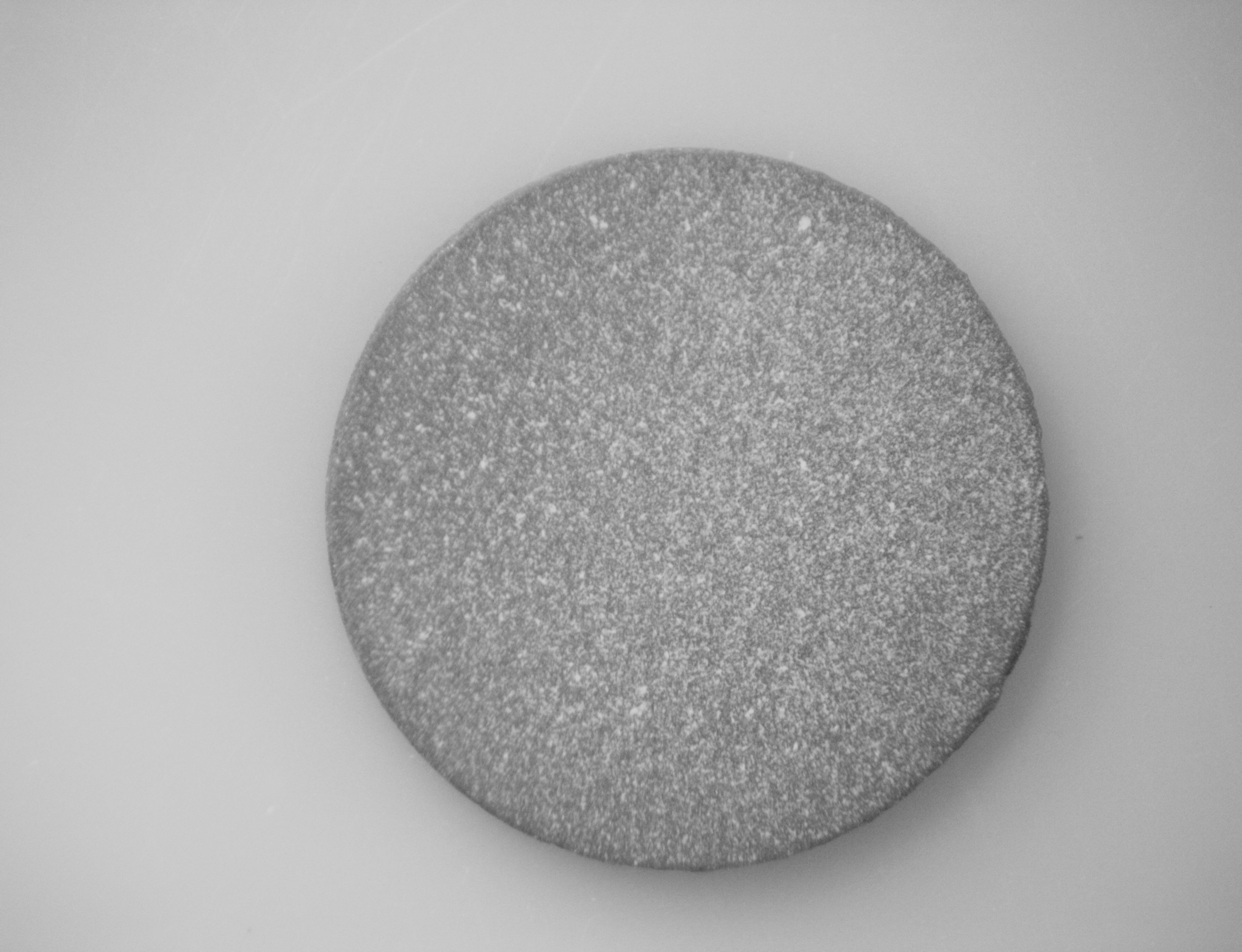

Supplement: Supplementary file 1 — Dataset for SREP-18-29489A [file 41598_2018_37123_MOESM1_ESM.zip › SupplementalDataFiles/Figure 6/0 hour/mHA_100.jpg]

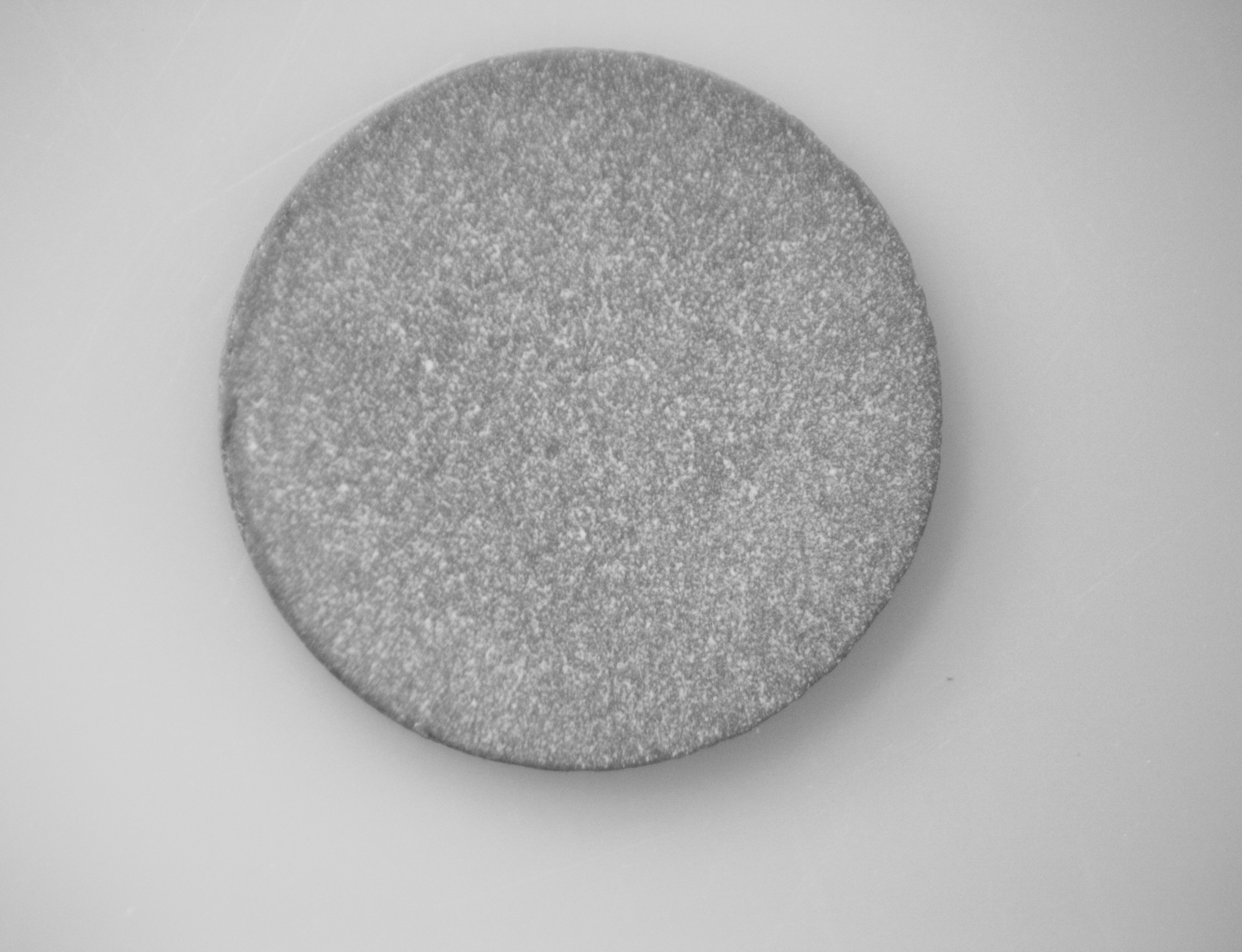

Supplement: Supplementary file 1 — Dataset for SREP-18-29489A [file 41598_2018_37123_MOESM1_ESM.zip › SupplementalDataFiles/Figure 6/0 hour/mHA_400.jpg]

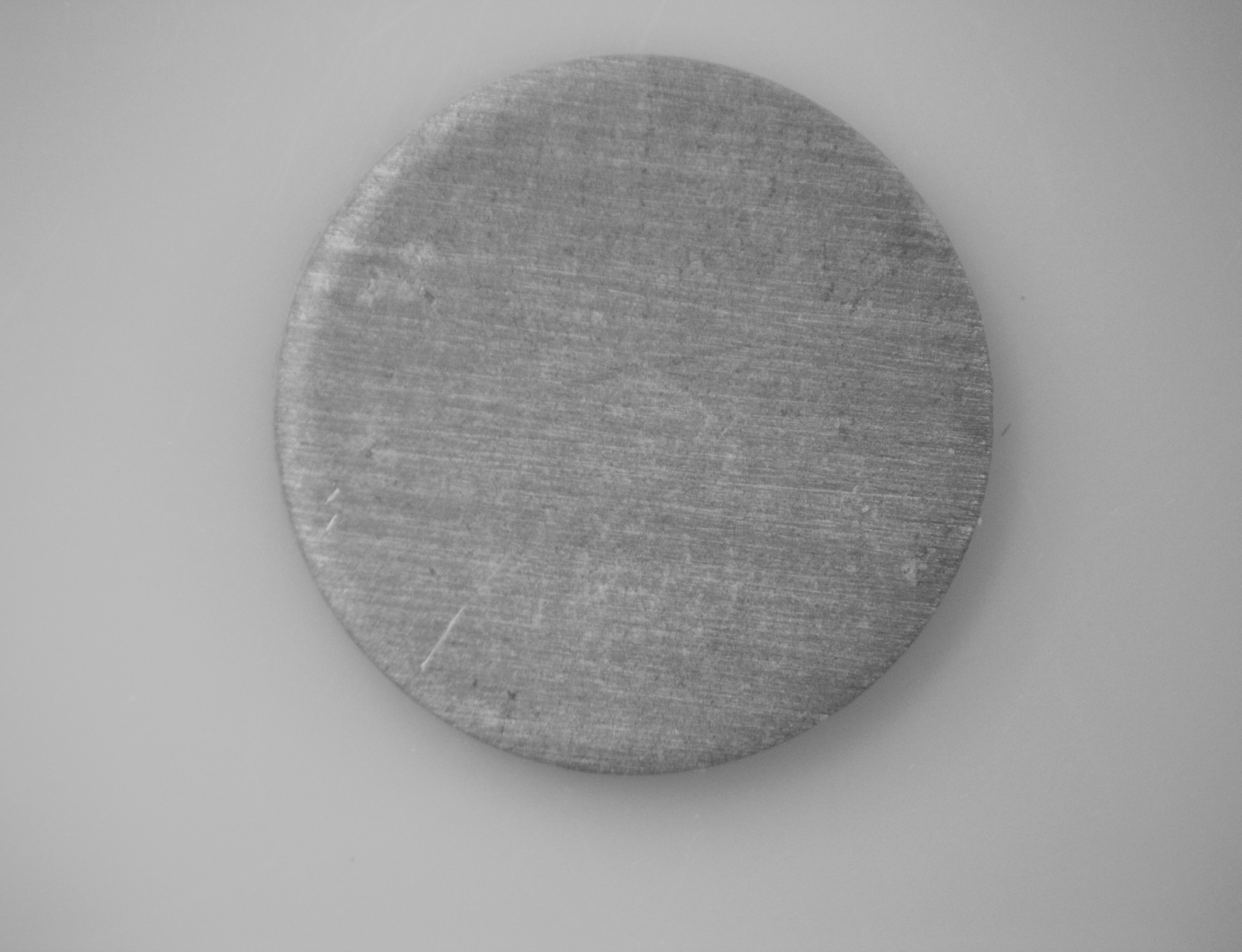

Supplement: Supplementary file 1 — Dataset for SREP-18-29489A [file 41598_2018_37123_MOESM1_ESM.zip › SupplementalDataFiles/Figure 6/0 hour/nHA_100.jpg]

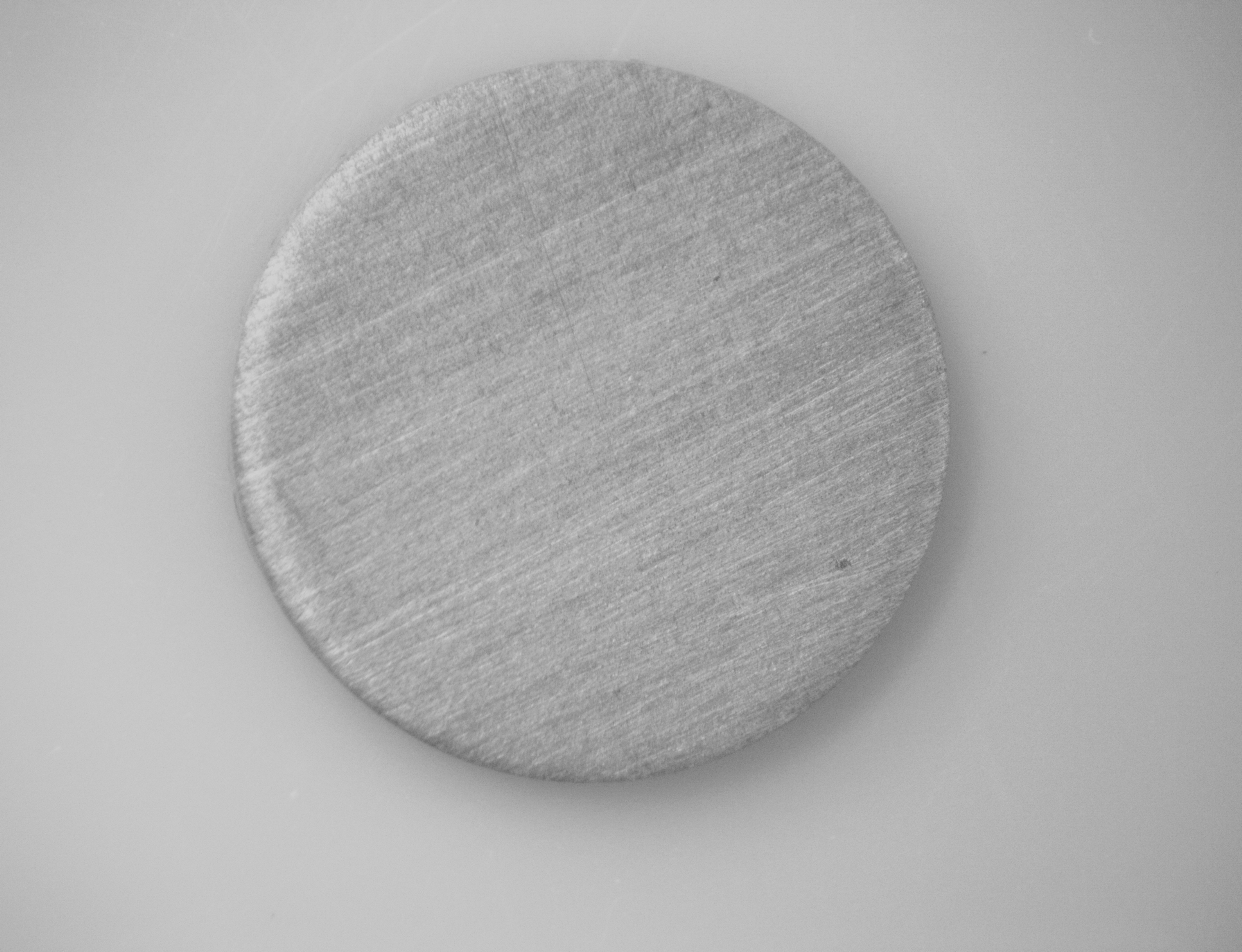

Supplement: Supplementary file 1 — Dataset for SREP-18-29489A [file 41598_2018_37123_MOESM1_ESM.zip › SupplementalDataFiles/Figure 6/0 hour/nHA_400.jpg]

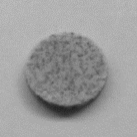

Supplement: Supplementary file 1 — Dataset for SREP-18-29489A [file 41598_2018_37123_MOESM1_ESM.zip › SupplementalDataFiles/Figure 6/1 wks/Mg.png]

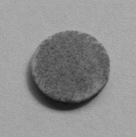

Supplement: Supplementary file 1 — Dataset for SREP-18-29489A [file 41598_2018_37123_MOESM1_ESM.zip › SupplementalDataFiles/Figure 6/1 wks/mHA_100.png]

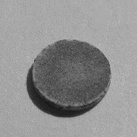

Supplement: Supplementary file 1 — Dataset for SREP-18-29489A [file 41598_2018_37123_MOESM1_ESM.zip › SupplementalDataFiles/Figure 6/1 wks/mHA_400.png]

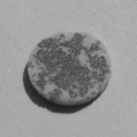

Supplement: Supplementary file 1 — Dataset for SREP-18-29489A [file 41598_2018_37123_MOESM1_ESM.zip › SupplementalDataFiles/Figure 6/1 wks/nHA_100.png]

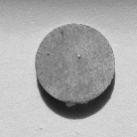

Supplement: Supplementary file 1 — Dataset for SREP-18-29489A [file 41598_2018_37123_MOESM1_ESM.zip › SupplementalDataFiles/Figure 6/12 hours/Mg.png]

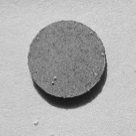

Supplement: Supplementary file 1 — Dataset for SREP-18-29489A [file 41598_2018_37123_MOESM1_ESM.zip › SupplementalDataFiles/Figure 6/12 hours/mHA_100.png]

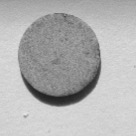

Supplement: Supplementary file 1 — Dataset for SREP-18-29489A [file 41598_2018_37123_MOESM1_ESM.zip › SupplementalDataFiles/Figure 6/12 hours/mHA_400.png]

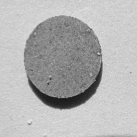

Supplement: Supplementary file 1 — Dataset for SREP-18-29489A [file 41598_2018_37123_MOESM1_ESM.zip › SupplementalDataFiles/Figure 6/12 hours/nHA_100.png]

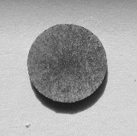

Supplement: Supplementary file 1 — Dataset for SREP-18-29489A [file 41598_2018_37123_MOESM1_ESM.zip › SupplementalDataFiles/Figure 6/12 hours/nHA_400.png]

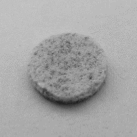

Supplement: Supplementary file 1 — Dataset for SREP-18-29489A [file 41598_2018_37123_MOESM1_ESM.zip › SupplementalDataFiles/Figure 6/2 wks/Mg.png]

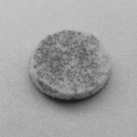

Supplement: Supplementary file 1 — Dataset for SREP-18-29489A [file 41598_2018_37123_MOESM1_ESM.zip › SupplementalDataFiles/Figure 6/2 wks/mHA_100.png]

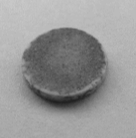

Supplement: Supplementary file 1 — Dataset for SREP-18-29489A [file 41598_2018_37123_MOESM1_ESM.zip › SupplementalDataFiles/Figure 6/2 wks/mHA_400.png]

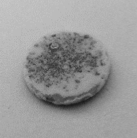

Supplement: Supplementary file 1 — Dataset for SREP-18-29489A [file 41598_2018_37123_MOESM1_ESM.zip › SupplementalDataFiles/Figure 6/2 wks/nHA_100.png]

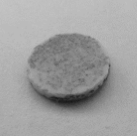

Supplement: Supplementary file 1 — Dataset for SREP-18-29489A [file 41598_2018_37123_MOESM1_ESM.zip › SupplementalDataFiles/Figure 6/2 wks/nHA_400.png]

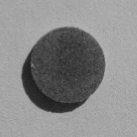

Supplement: Supplementary file 1 — Dataset for SREP-18-29489A [file 41598_2018_37123_MOESM1_ESM.zip › SupplementalDataFiles/Figure 6/24 hours/Mg.png]

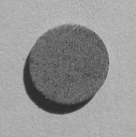

Supplement: Supplementary file 1 — Dataset for SREP-18-29489A [file 41598_2018_37123_MOESM1_ESM.zip › SupplementalDataFiles/Figure 6/24 hours/mHA_100.png]

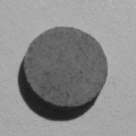

Supplement: Supplementary file 1 — Dataset for SREP-18-29489A [file 41598_2018_37123_MOESM1_ESM.zip › SupplementalDataFiles/Figure 6/24 hours/mHA_400.png]

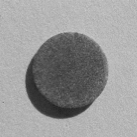

Supplement: Supplementary file 1 — Dataset for SREP-18-29489A [file 41598_2018_37123_MOESM1_ESM.zip › SupplementalDataFiles/Figure 6/24 hours/nHA_100.png]

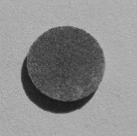

Supplement: Supplementary file 1 — Dataset for SREP-18-29489A [file 41598_2018_37123_MOESM1_ESM.zip › SupplementalDataFiles/Figure 6/24 hours/nHA_400.png]

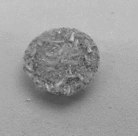

Supplement: Supplementary file 1 — Dataset for SREP-18-29489A [file 41598_2018_37123_MOESM1_ESM.zip › SupplementalDataFiles/Figure 6/4 wks/Mg.png]

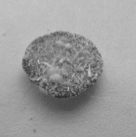

Supplement: Supplementary file 1 — Dataset for SREP-18-29489A [file 41598_2018_37123_MOESM1_ESM.zip › SupplementalDataFiles/Figure 6/4 wks/mHA_100.png]

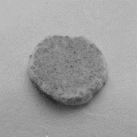

Supplement: Supplementary file 1 — Dataset for SREP-18-29489A [file 41598_2018_37123_MOESM1_ESM.zip › SupplementalDataFiles/Figure 6/4 wks/mHA_400.png]

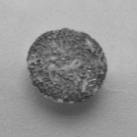

Supplement: Supplementary file 1 — Dataset for SREP-18-29489A [file 41598_2018_37123_MOESM1_ESM.zip › SupplementalDataFiles/Figure 6/4 wks/nHA_100.png]

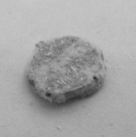

Supplement: Supplementary file 1 — Dataset for SREP-18-29489A [file 41598_2018_37123_MOESM1_ESM.zip › SupplementalDataFiles/Figure 6/4 wks/nHA_400.png]

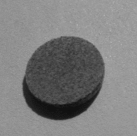

Supplement: Supplementary file 1 — Dataset for SREP-18-29489A [file 41598_2018_37123_MOESM1_ESM.zip › SupplementalDataFiles/Figure 6/48 hours/Mg.png]

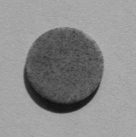

Supplement: Supplementary file 1 — Dataset for SREP-18-29489A [file 41598_2018_37123_MOESM1_ESM.zip › SupplementalDataFiles/Figure 6/48 hours/mHA_100.png]

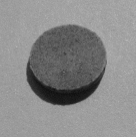

Supplement: Supplementary file 1 — Dataset for SREP-18-29489A [file 41598_2018_37123_MOESM1_ESM.zip › SupplementalDataFiles/Figure 6/48 hours/mHA_400.png]

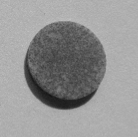

Supplement: Supplementary file 1 — Dataset for SREP-18-29489A [file 41598_2018_37123_MOESM1_ESM.zip › SupplementalDataFiles/Figure 6/48 hours/nHA_100.png]

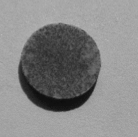

Supplement: Supplementary file 1 — Dataset for SREP-18-29489A [file 41598_2018_37123_MOESM1_ESM.zip › SupplementalDataFiles/Figure 6/48 hours/nHA_400.png]

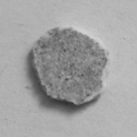

Supplement: Supplementary file 1 — Dataset for SREP-18-29489A [file 41598_2018_37123_MOESM1_ESM.zip › SupplementalDataFiles/Figure 6/6 wks/Mg.png]

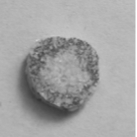

Supplement: Supplementary file 1 — Dataset for SREP-18-29489A [file 41598_2018_37123_MOESM1_ESM.zip › SupplementalDataFiles/Figure 6/6 wks/mHA_100.png]

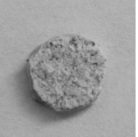

Supplement: Supplementary file 1 — Dataset for SREP-18-29489A [file 41598_2018_37123_MOESM1_ESM.zip › SupplementalDataFiles/Figure 6/6 wks/mHA_400.png]

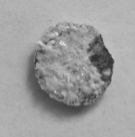

Supplement: Supplementary file 1 — Dataset for SREP-18-29489A [file 41598_2018_37123_MOESM1_ESM.zip › SupplementalDataFiles/Figure 6/6 wks/nHA_100.png]

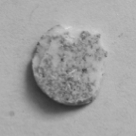

Supplement: Supplementary file 1 — Dataset for SREP-18-29489A [file 41598_2018_37123_MOESM1_ESM.zip › SupplementalDataFiles/Figure 6/6 wks/nHA_400.png]

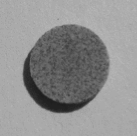

Supplement: Supplementary file 1 — Dataset for SREP-18-29489A [file 41598_2018_37123_MOESM1_ESM.zip › SupplementalDataFiles/Figure 6/72 hours/Mg.png]

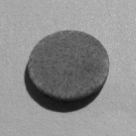

Supplement: Supplementary file 1 — Dataset for SREP-18-29489A [file 41598_2018_37123_MOESM1_ESM.zip › SupplementalDataFiles/Figure 6/72 hours/mHA_100.png]

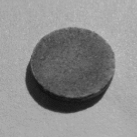

Supplement: Supplementary file 1 — Dataset for SREP-18-29489A [file 41598_2018_37123_MOESM1_ESM.zip › SupplementalDataFiles/Figure 6/72 hours/mHA_400.png]

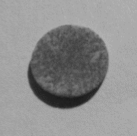

Supplement: Supplementary file 1 — Dataset for SREP-18-29489A [file 41598_2018_37123_MOESM1_ESM.zip › SupplementalDataFiles/Figure 6/72 hours/nHA_100.png]

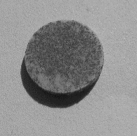

Supplement: Supplementary file 1 — Dataset for SREP-18-29489A [file 41598_2018_37123_MOESM1_ESM.zip › SupplementalDataFiles/Figure 6/72 hours/nHA_400.png]

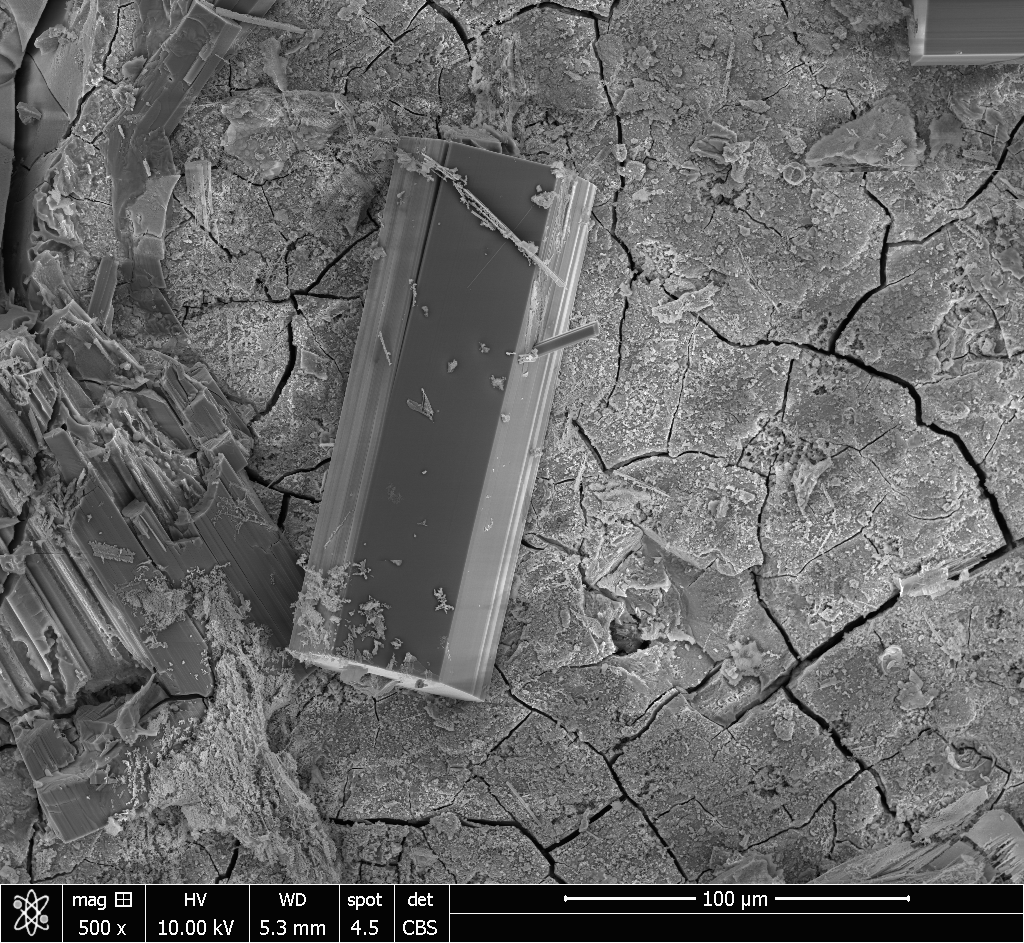

Supplement: Supplementary file 1 — Dataset for SREP-18-29489A [file 41598_2018_37123_MOESM1_ESM.zip › SupplementalDataFiles/Figure 7/Figure 7(a1).tif]

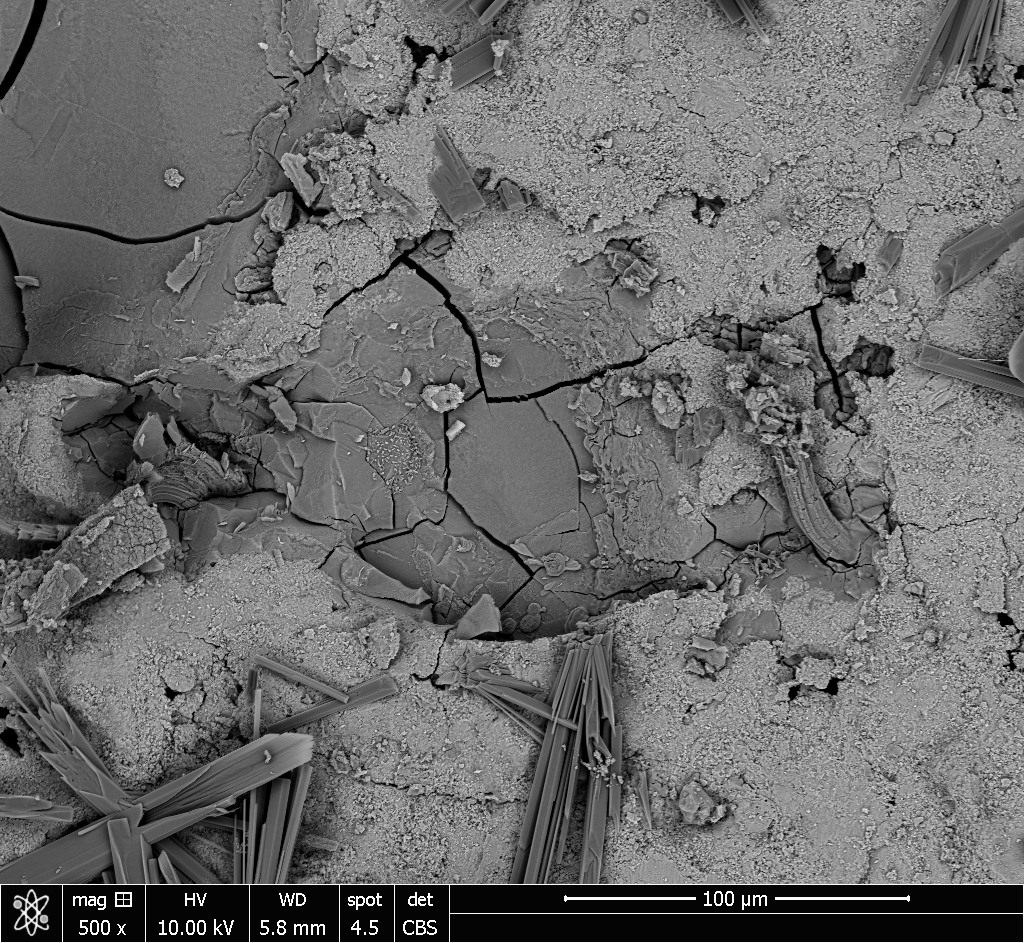

Supplement: Supplementary file 1 — Dataset for SREP-18-29489A [file 41598_2018_37123_MOESM1_ESM.zip › SupplementalDataFiles/Figure 7/Figure 7(a2).tif]

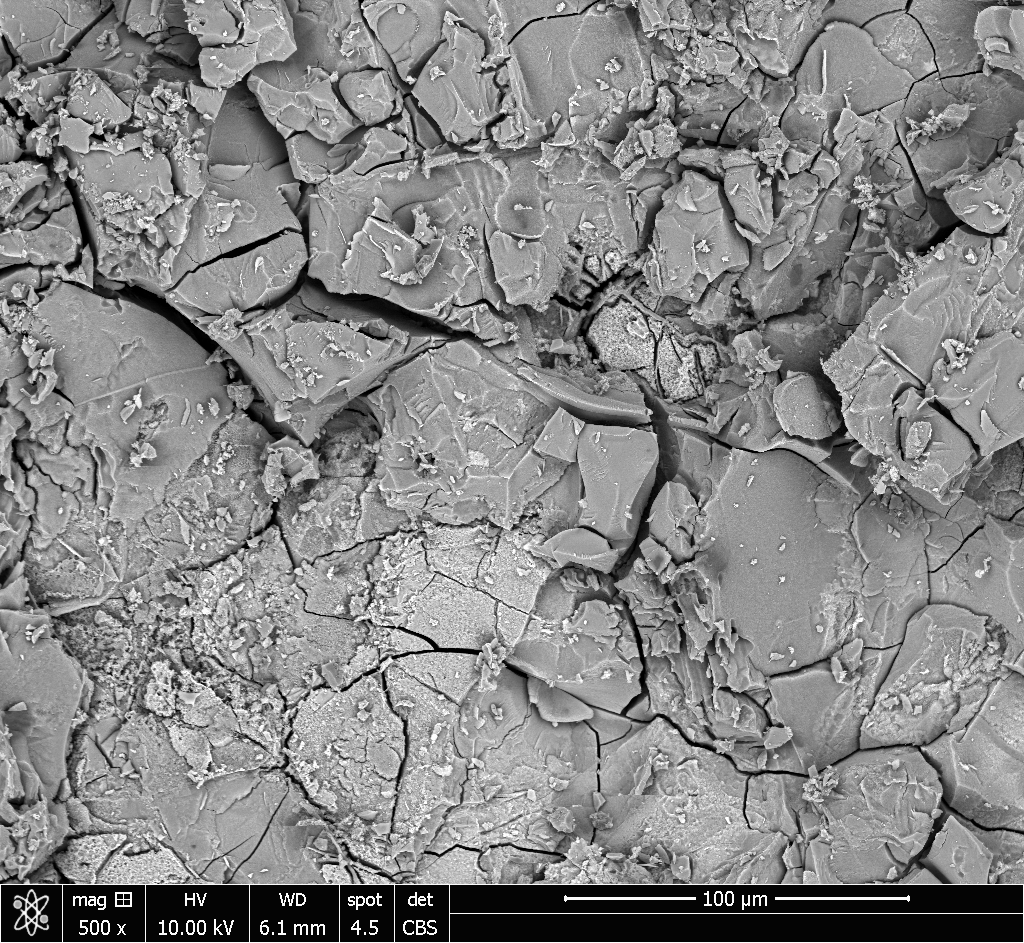

Supplement: Supplementary file 1 — Dataset for SREP-18-29489A [file 41598_2018_37123_MOESM1_ESM.zip › SupplementalDataFiles/Figure 7/Figure 7(b1).tif]

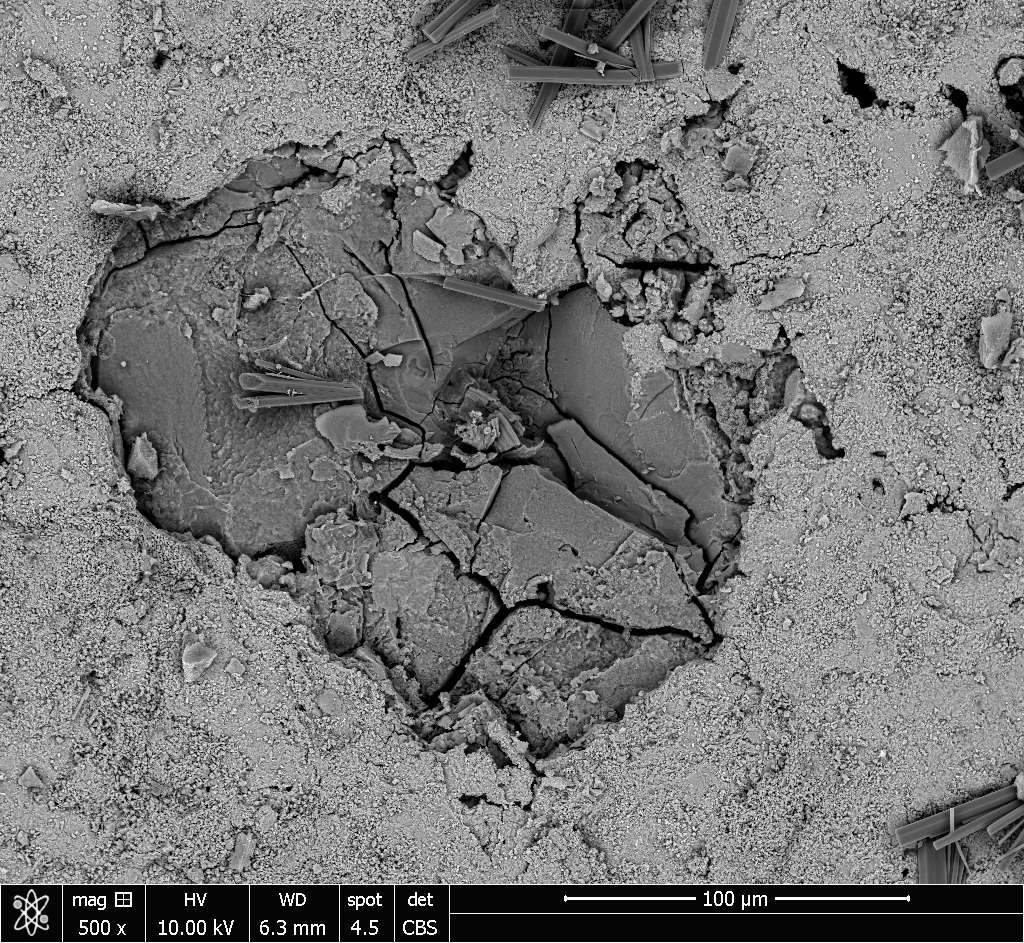

Supplement: Supplementary file 1 — Dataset for SREP-18-29489A [file 41598_2018_37123_MOESM1_ESM.zip › SupplementalDataFiles/Figure 7/Figure 7(b2).tif]

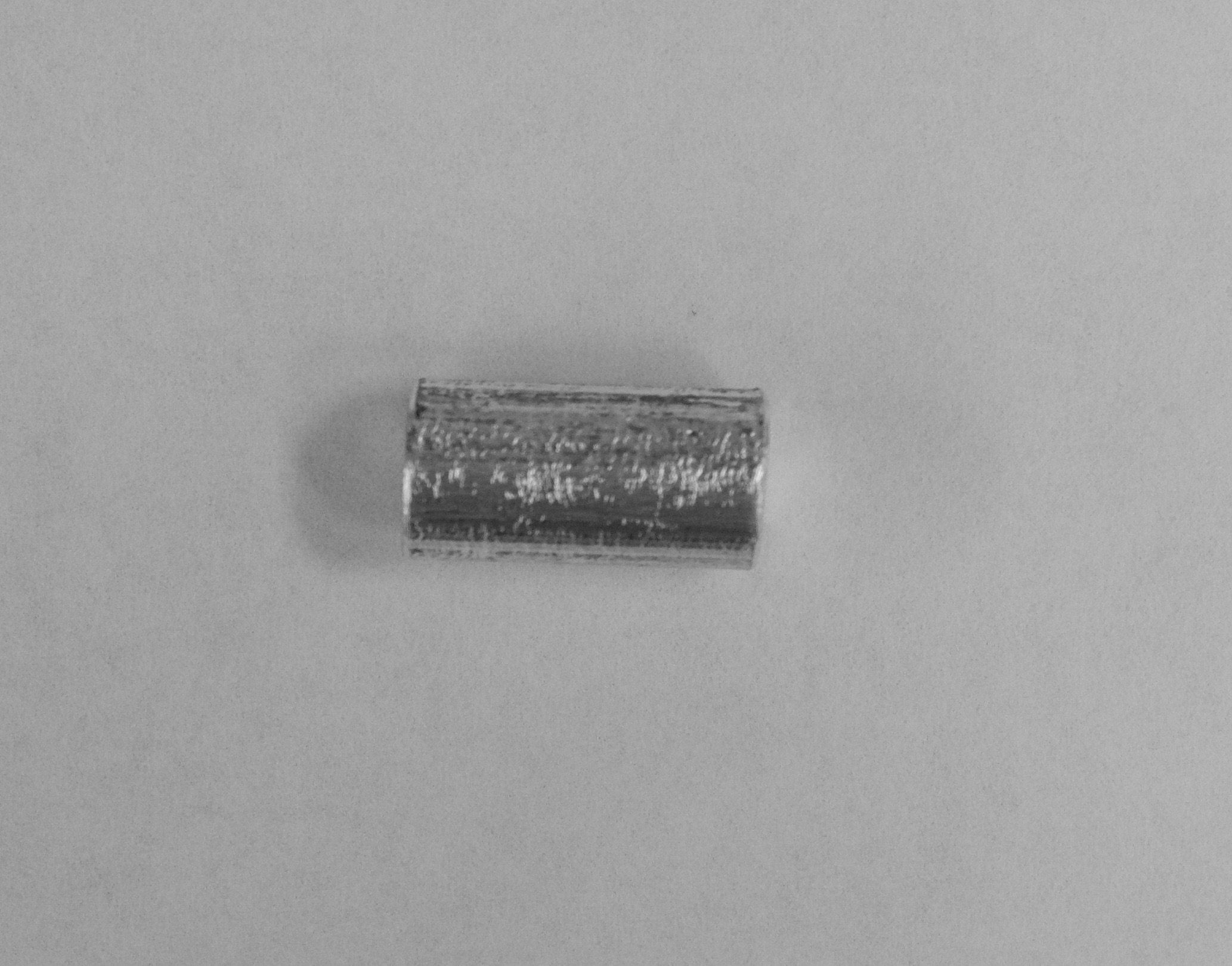

Supplement: Supplementary file 1 — Dataset for SREP-18-29489A [file 41598_2018_37123_MOESM1_ESM.zip › SupplementalDataFiles/Figure 8/0 hour/Mg rod.jpg]

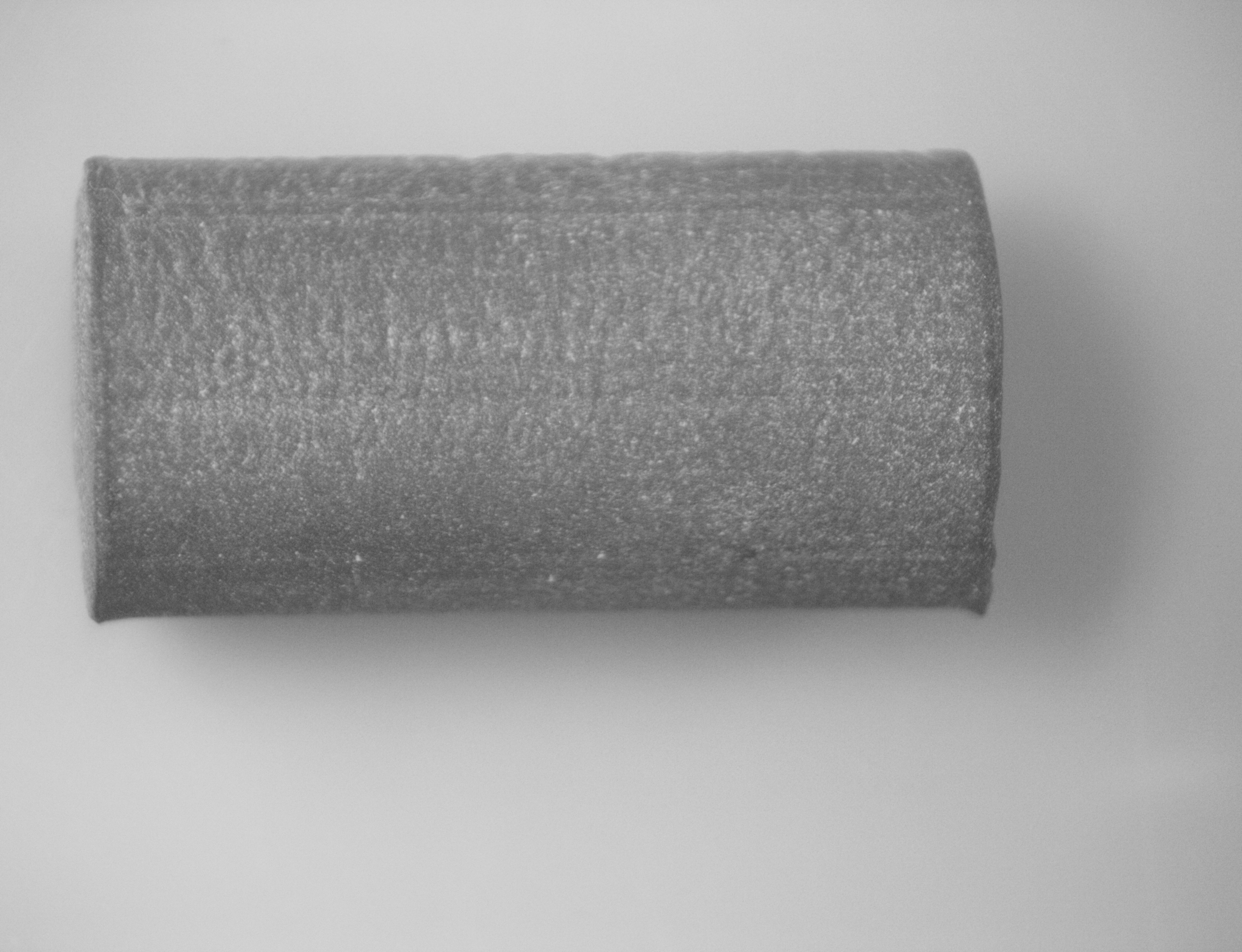

Supplement: Supplementary file 1 — Dataset for SREP-18-29489A [file 41598_2018_37123_MOESM1_ESM.zip › SupplementalDataFiles/Figure 8/0 hour/mHA_100 rod.jpg]

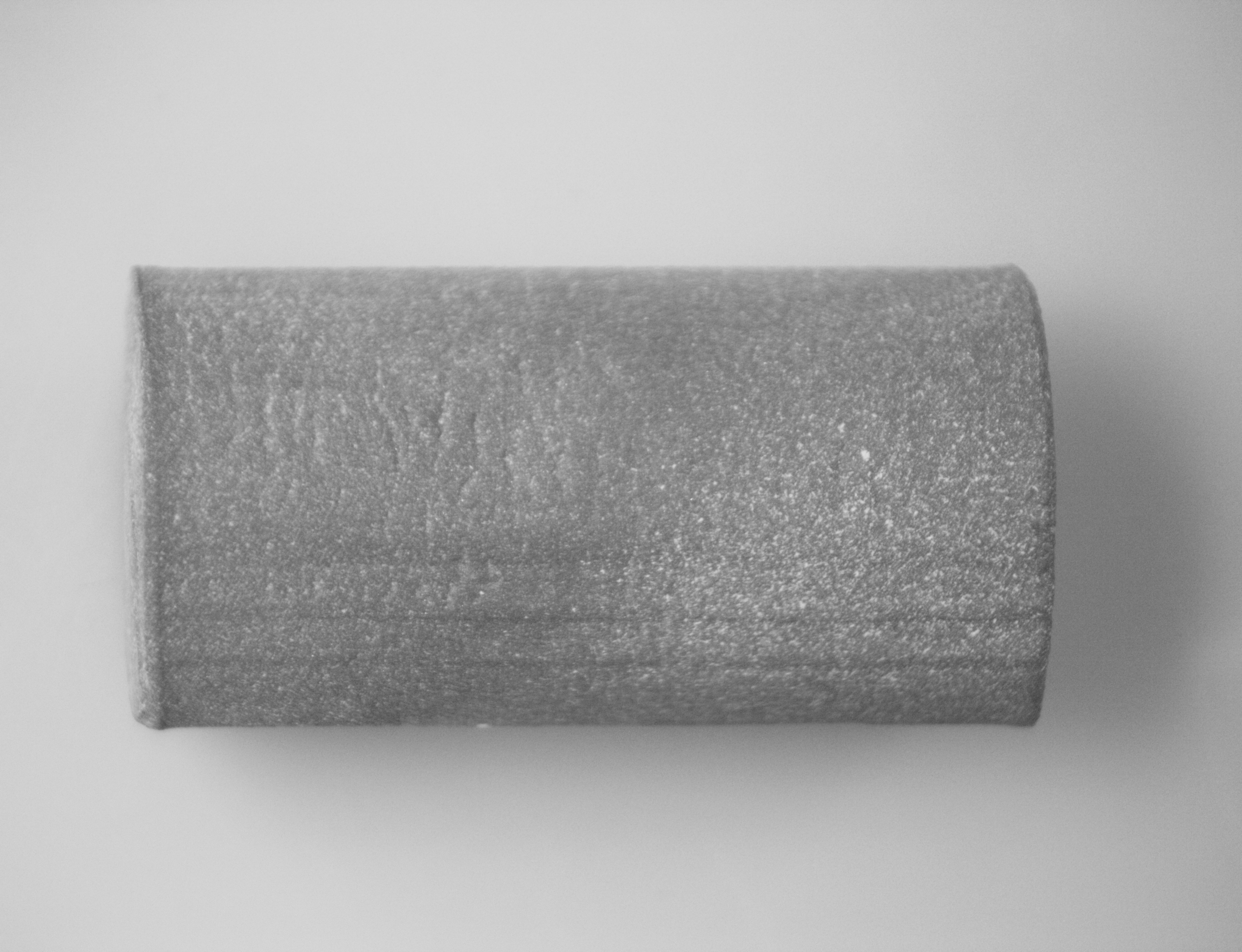

Supplement: Supplementary file 1 — Dataset for SREP-18-29489A [file 41598_2018_37123_MOESM1_ESM.zip › SupplementalDataFiles/Figure 8/0 hour/mHA_400 rod.jpg]

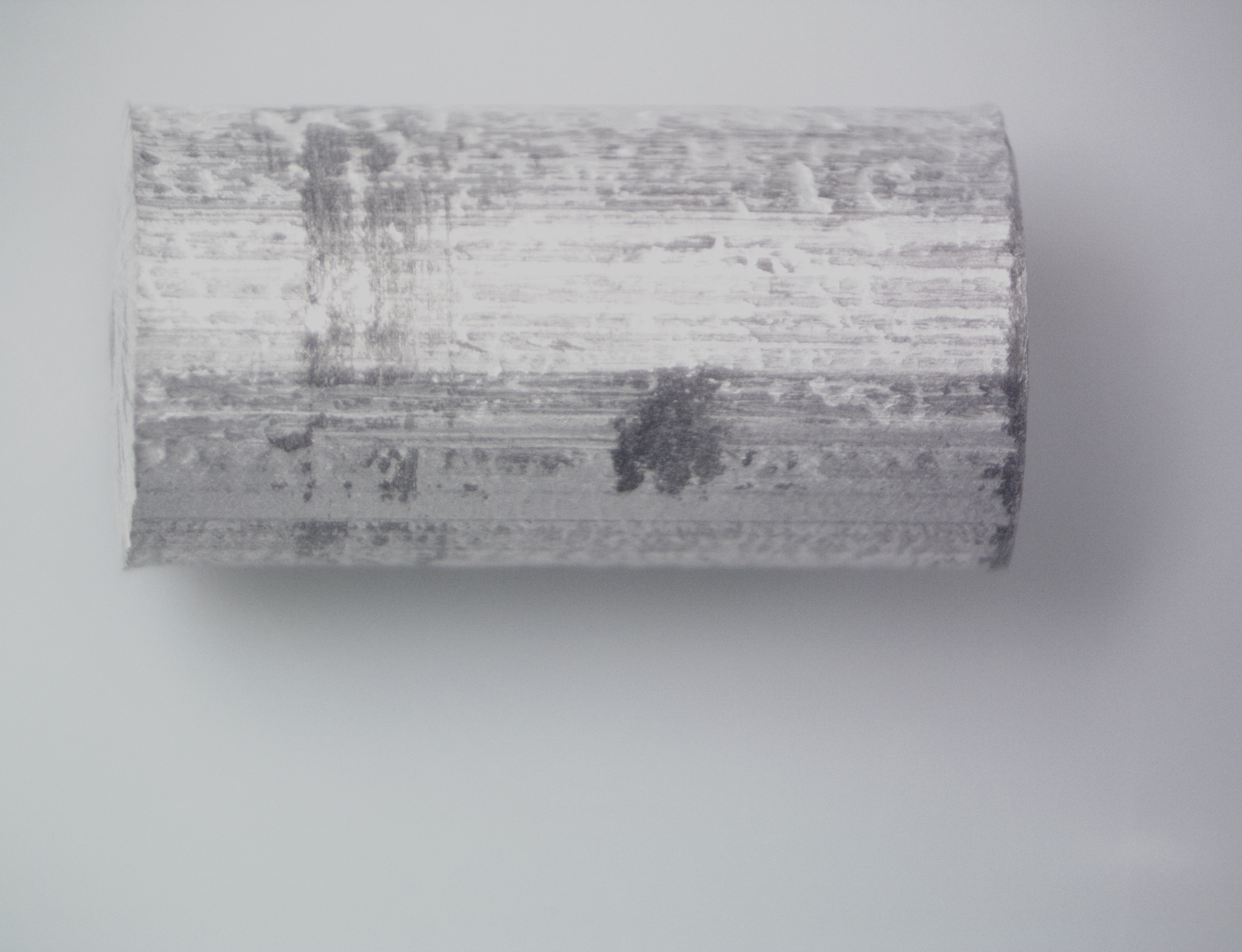

Supplement: Supplementary file 1 — Dataset for SREP-18-29489A [file 41598_2018_37123_MOESM1_ESM.zip › SupplementalDataFiles/Figure 8/0 hour/nHA_100 rod.jpg]

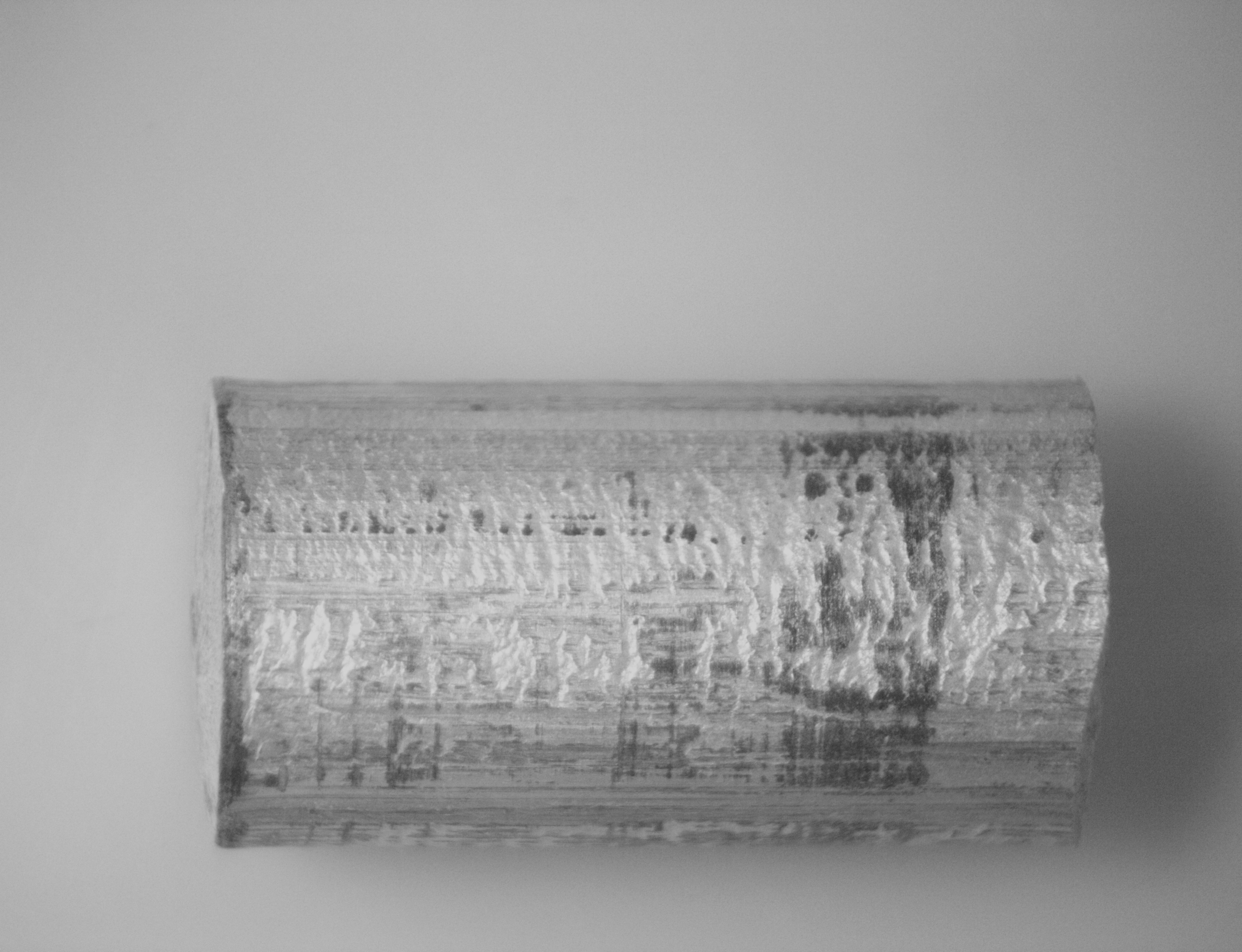

Supplement: Supplementary file 1 — Dataset for SREP-18-29489A [file 41598_2018_37123_MOESM1_ESM.zip › SupplementalDataFiles/Figure 8/0 hour/nHA_400 rod.jpg]

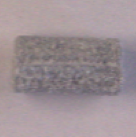

Supplement: Supplementary file 1 — Dataset for SREP-18-29489A [file 41598_2018_37123_MOESM1_ESM.zip › SupplementalDataFiles/Figure 8/1 wks/Mg.png]

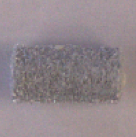

Supplement: Supplementary file 1 — Dataset for SREP-18-29489A [file 41598_2018_37123_MOESM1_ESM.zip › SupplementalDataFiles/Figure 8/1 wks/mHA_100.png]

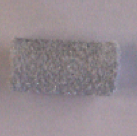

Supplement: Supplementary file 1 — Dataset for SREP-18-29489A [file 41598_2018_37123_MOESM1_ESM.zip › SupplementalDataFiles/Figure 8/1 wks/mHA_400.png]

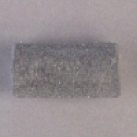

Supplement: Supplementary file 1 — Dataset for SREP-18-29489A [file 41598_2018_37123_MOESM1_ESM.zip › SupplementalDataFiles/Figure 8/1 wks/nHA_100.png]

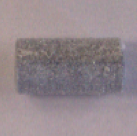

Supplement: Supplementary file 1 — Dataset for SREP-18-29489A [file 41598_2018_37123_MOESM1_ESM.zip › SupplementalDataFiles/Figure 8/1 wks/nHA_400.png]

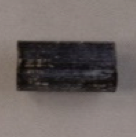

Supplement: Supplementary file 1 — Dataset for SREP-18-29489A [file 41598_2018_37123_MOESM1_ESM.zip › SupplementalDataFiles/Figure 8/12 hours/Mg.png]

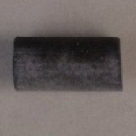

Supplement: Supplementary file 1 — Dataset for SREP-18-29489A [file 41598_2018_37123_MOESM1_ESM.zip › SupplementalDataFiles/Figure 8/12 hours/mHA_100.png]

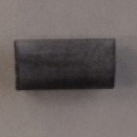

Supplement: Supplementary file 1 — Dataset for SREP-18-29489A [file 41598_2018_37123_MOESM1_ESM.zip › SupplementalDataFiles/Figure 8/12 hours/mHA_400.png]

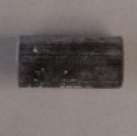

Supplement: Supplementary file 1 — Dataset for SREP-18-29489A [file 41598_2018_37123_MOESM1_ESM.zip › SupplementalDataFiles/Figure 8/12 hours/nHA_100.png]

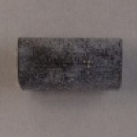

Supplement: Supplementary file 1 — Dataset for SREP-18-29489A [file 41598_2018_37123_MOESM1_ESM.zip › SupplementalDataFiles/Figure 8/12 hours/nHA_400.png]

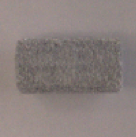

Supplement: Supplementary file 1 — Dataset for SREP-18-29489A [file 41598_2018_37123_MOESM1_ESM.zip › SupplementalDataFiles/Figure 8/2 wks/Mg.png]

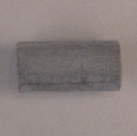

Supplement: Supplementary file 1 — Dataset for SREP-18-29489A [file 41598_2018_37123_MOESM1_ESM.zip › SupplementalDataFiles/Figure 8/2 wks/mHA_100.png]

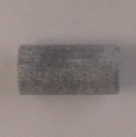

Supplement: Supplementary file 1 — Dataset for SREP-18-29489A [file 41598_2018_37123_MOESM1_ESM.zip › SupplementalDataFiles/Figure 8/2 wks/mHA_400.png]

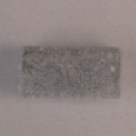

Supplement: Supplementary file 1 — Dataset for SREP-18-29489A [file 41598_2018_37123_MOESM1_ESM.zip › SupplementalDataFiles/Figure 8/2 wks/nHA_100.png]

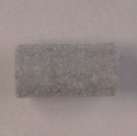

Supplement: Supplementary file 1 — Dataset for SREP-18-29489A [file 41598_2018_37123_MOESM1_ESM.zip › SupplementalDataFiles/Figure 8/2 wks/nHA_400.png]

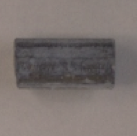

Supplement: Supplementary file 1 — Dataset for SREP-18-29489A [file 41598_2018_37123_MOESM1_ESM.zip › SupplementalDataFiles/Figure 8/24 hours/Mg.png]

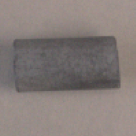

Supplement: Supplementary file 1 — Dataset for SREP-18-29489A [file 41598_2018_37123_MOESM1_ESM.zip › SupplementalDataFiles/Figure 8/24 hours/mHA_100.png]

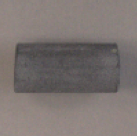

Supplement: Supplementary file 1 — Dataset for SREP-18-29489A [file 41598_2018_37123_MOESM1_ESM.zip › SupplementalDataFiles/Figure 8/24 hours/mHA_400.png]
